# Temporally resolved proteomics identifies nidogen-2 as a cotarget in pancreatic cancer that modulates fibrosis and therapy response

**DOI:** 10.1126/sciadv.adl1197

**Published:** 2024-07-03

**Authors:** Brooke A. Pereira, Shona Ritchie, Cecilia R. Chambers, Katie A. Gordon, Astrid Magenau, Kendelle J. Murphy, Max Nobis, Victoria M. Tyma, Ying Fei Liew, Morghan C. Lucas, Marjan M. Naeini, Deborah S. Barkauskas, Diego Chacon-Fajardo, Anna E. Howell, Amelia L. Parker, Sean C. Warren, Daniel A. Reed, Victoria Lee, Xanthe L. Metcalf, Young Kyung Lee, Luke P. O’Regan, Jessie Zhu, Michael Trpceski, Angela R. M. Fontaine, Janett Stoehr, Romain Rouet, Xufeng Lin, Jessica L. Chitty, Sean Porazinski, Sunny Z. Wu, Elysse C. Filipe, Antonia L. Cadell, Holly Holliday, Jessica Yang, Michael Papanicolaou, Ruth J. Lyons, Anaiis Zaratzian, Michael Tayao, Andrew Da Silva, Claire Vennin, Julia Yin, Alysha B. Dew, Paul J. McMillan, Leonard D. Goldstein, Ira W. Deveson, David R. Croucher, Michael S. Samuel, Hao-Wen Sim, Marcel Batten, Lorraine Chantrill, Sean M. Grimmond, Anthony J. Gill, Jaswinder Samra, Thomas R. Jeffry Evans, Takako Sasaki, Tri G. Phan, Alexander Swarbrick, Owen J. Sansom, Jennifer P. Morton, Australian Pancreatic Cancer Matrix Atlas (APMA), Australian Pancreatic Cancer Genome Initiative (APGI), Marina Pajic, Benjamin L. Parker, David Herrmann, Thomas R. Cox, Paul Timpson

**Affiliations:** ^1^ Cancer Ecosystems Program, Garvan Institute of Medical Research and The Kinghorn Cancer Centre, Darlinghurst, New South Wales, Australia.; ^2^ School of Clinical Medicine, Faculty of Medicine, University of New South Wales (UNSW) Sydney, Kensington, New South Wales, Australia.; ^3^ Intravital Imaging Expertise Center, VIB Center for Cancer Biology, VIB, Leuven, Belgium.; ^4^ Centre for Genomic Regulation (CRG), Barcelona Institute of Science and Technology, Barcelona, Spain.; ^5^ Universitat Pompeu Fabra (UPF), Barcelona, Spain.; ^6^ Genomics and Inherited Disease Program, Garvan Institute of Medical Research and The Kinghorn Cancer Centre, Darlinghurst, New South Wales, Australia.; ^7^ ACRF INCITe Intravital Imaging Centre, Garvan Institute of Medical Research and The Kinghorn Cancer Centre, Darlinghurst, New South Wales, Australia.; ^8^ Translational Oncology Program, Garvan Institute of Medical Research and The Kinghorn Cancer Centre, Darlinghurst, New South Wales, Australia.; ^9^ Immune Biotherapies Program, Garvan Institute of Medical Research, Darlinghurst, New South Wales, Australia.; ^10^ Data Science Platform, Garvan Institute of Medical Research, Darlinghurst, New South Wales, Australia.; ^11^ Genentech Inc., South San Francisco, CA, USA.; ^12^ Children’s Cancer Institute, Lowy Cancer Research Centre, UNSW Sydney, Kensington, New South Wales, Australia.; ^13^ Histopathology Platform, Garvan Institute of Medical Research and The Kinghorn Cancer Centre, Darlinghurst, New South Wales, Australia.; ^14^ Division of Molecular Pathology, Netherlands Cancer Institute, Antoni van Leeuwenhoek Hospital, Amsterdam, Netherlands.; ^15^ Oncode Institute, Amsterdam, Netherlands.; ^16^ Centre for Advanced Histology & Microscopy, Peter MacCallum Cancer Centre, Parkville, Victoria, Australia.; ^17^ Sir Peter MacCallum Department of Oncology, The University of Melbourne, Parkville, Victoria, Australia.; ^18^ Biological Optical Microscopy Platform, The University of Melbourne, Parkville, Victoria, Australia.; ^19^ Centre for Cancer Biology, An Alliance of SA Pathology and University of South Australia, Adelaide, South Australia, Australia.; ^20^ Basil Hetzel Institute for Translational Health Research, Queen Elizabeth Hospital, Woodville South, South Australia, Australia.; ^21^ NHMRC Clinical Trials Centre, University of Sydney, Camperdown, New South Wales, Australia.; ^22^ Department of Medical Oncology, Chris O’Brien Lifehouse, Camperdown, New South Wales, Australia.; ^23^ Department of Medical Oncology, Illawarra Shoalhaven Local Health District, Wollongong, New South Wales, Australia.; ^24^ Centre for Cancer Research and Department of Clinical Pathology, The University of Melbourne, Parkville, Victoria, Australia.; ^25^ NSW Health Pathology, Department of Anatomical Pathology, Royal North Shore Hospital, St Leonards, New South Wales, Australia.; ^26^ Sydney Medical School, University of Sydney, Camperdown, New South Wales, Australia.; ^27^ Department of Surgery, Royal North Shore Hospital, St Leonards, New South Wales, Australia.; ^28^ Cancer Research UK Beatson Institute, Glasgow, UK.; ^29^ School of Cancer Sciences, Institute of Cancer Sciences, University of Glasgow, Glasgow, UK.; ^30^ Department of Biochemistry, Faculty of Medicine, Oita University, Oita, Japan.; ^31^ Precision Immunology Program, Garvan Institute of Medical Research, Darlinghurst, New South Wales, Australia.; ^32^ Department of Anatomy and Physiology, University of Melbourne, Parkville, Victoria, Australia.

## Abstract

Pancreatic ductal adenocarcinoma (PDAC) is characterized by increasing fibrosis, which can enhance tumor progression and spread. Here, we undertook an unbiased temporal assessment of the matrisome of the highly metastatic KPC (*Pdx1-Cre*, *LSL-Kras^G12D/+^*, *LSL-Trp53^R172H/+^*) and poorly metastatic KP^fl^C (*Pdx1-Cre*, *LSL-Kras^G12D/+^*, *Trp53^fl/+^*) genetically engineered mouse models of pancreatic cancer using mass spectrometry proteomics. Our assessment at early-, mid-, and late-stage disease reveals an increased abundance of nidogen-2 (NID2) in the KPC model compared to KP^fl^C, with further validation showing that NID2 is primarily expressed by cancer-associated fibroblasts (CAFs). Using biomechanical assessments, second harmonic generation imaging, and birefringence analysis, we show that NID2 reduction by CRISPR interference (CRISPRi) in CAFs reduces stiffness and matrix remodeling in three-dimensional models, leading to impaired cancer cell invasion. Intravital imaging revealed improved vascular patency in live NID2-depleted tumors, with enhanced response to gemcitabine/Abraxane. In orthotopic models, NID2 CRISPRi tumors had less liver metastasis and increased survival, highlighting NID2 as a potential PDAC cotarget.

## Teaser

Temporal mass spectrometry proteomics, advanced microscopy, and in vivo modeling reveal nidogen-2 as a cotarget in PDAC.

## INTRODUCTION

Pancreatic ductal adenocarcinoma (PDAC) is a highly metastatic and treatment-resistant malignancy, with a 5-year survival rate of ~11% (*1*). Survival rates have remained largely unchanged over the past 40 years, with new therapeutic options desperately needed. Over 80% of patients with PDAC present with metastatic disease, where patients are not eligible for surgical resection and systemic chemotherapies are standard of care (*2*). Unfortunately, PDAC rapidly acquires resistance to chemotherapy, which only extends survival in the order of months (*2*). Evidently, new treatment approaches are urgently needed to improve outcomes in this aggressive disease.

For the majority of PDAC cases, an initiating *KRAS* mutation has been identified (>90% of cases) in combination with secondary mutations such as alterations in *TP53* (>50% of cases) (*3*). The activation of these mutations is accompanied by histopathological changes from precursor lesions known as pancreatic intraepithelial neoplasia (PanINs) to invasive PDAC. During pancreatic cancer initiation and development, complex tumor-stromal reciprocal interactions occur, leading to a fibrotic tumor microenvironment (TME) (*4*–*7*). Pancreatic tumor cells activate and reprogram stromal cells, resulting in the formation of a diverse and dynamic population of cancer-associated fibroblasts (CAFs) (*8*–*13*), which can have anti- and/or protumorigenic roles (*4*–*7*). CAFs are the principal source of the fibrotic extracellular matrix (ECM) proteins in the pancreatic TME. This fibrosis causes aberrant downstream biomechanical effects in the tumor milieu (*14*–*16*), which are linked to poor patient outcomes (*17*). Increasing fibrosis has also been shown to enhance tumor progression and block drug penetrance by increasing interstitial fluid pressure, as well as causing vascular remodeling and hypovascularization (*15*, *18*–*20*). Consequently, pancreatic fibrosis can protect the tumor from intratumoral drug efficacy and the surveying immune system (*21*–*23*).

Considering the central role of CAF-derived fibrosis, there have been many efforts to cotarget CAFs or fibrotic proteins in combination with chemotherapy. However, several notable studies have shown that widespread ablation of the stroma (*24*–*26*) or targeting stromal components with intrinsic redundancy (*27*) can enhance tumor progression, suggesting that a more fine-tuned approach to fibrosis targeting is required. We and others have shown that context-dependent cotargeting of specific fibrotic components within the pancreatic TME can improve therapeutic efficacy and impair metastasis in preclinical models (*15*, *19*, *20*, *28*–*32*). Thus, there is an imperative to find and test new protumorigenic matrix cotargets in the pancreatic TME, which could then be modulated to reduce fibrosis to potentially improve therapy response in pancreatic cancer.

Mass spectrometry (MS) proteomics offers a highly accurate and unbiased approach to studying the matrix proteins (matrisome) of solid tumors such as pancreatic cancer (*33*). Here, we adapt ISDoT (in situ decellularization of tissues) technology (*34*, *35*) to enrich for matrisomal proteins, coupled with label-free data-independent acquisition liquid chromatography–tandem MS (DIA LC-MS/MS) to dissect the fibrotic signatures of pancreatic tumors from two commonly used genetically engineered mouse models (GEMMs) of pancreatic cancer: the highly metastatic KPC (*Pdx1-Cre*, *LSL-Kras^G12D/+^*, *LSL-Trp53^R172H/+^*) and poorly metastatic KP^fl^C (*Pdx1-Cre*, *LSL-Kras^G12D/+^*, *Trp53^fl/+^*) mouse models (*36*, *37*). The KP^fl^C mouse model, in which pancreatic cancer cells have lost p53 expression, is characterized by poor metastatic spread (*37*) and lower levels of fibrosis (*28*) compared to the highly metastatic KPC mouse model, which expresses a gain-of-function p53 R172H mutation (mouse analog; equivalent to human R175H), commonly found in patient pancreatic tumors. Here, we analyze decellularized tumors from age-matched KPC, KP^fl^C, and wild-type (WT) control mice at three different stages during tumor development (early, mid, and late) to identify temporally regulated matrix drivers of metastatic disease.

Through our analysis, we identify nidogen-2 (NID2) to be enriched in the highly metastatic KPC model compared to the poorly metastatic KP^fl^C model at mid-stage disease. NID2 (also known as entactin-2) is a basement membrane (BM) glycoprotein (*38*, *39*). Nidogens are elongated molecules composed of three globular domains (G1, G2, and G3) connected by a flexible, protease-sensitive link and a rigid rod-like domain (*38*). These glycoproteins are considered classical linkers, joining laminin and collagen IV networks at BMs, and are critical in embryonic development. In cancer, high NID2 expression has previously been associated with several solid malignancies including gastric cancer (*40*), esophageal squamous cell carcinoma (*41*), ovarian cancer (*42*), melanoma (*43*), and breast cancer (*44*, *45*), although its causal role in pancreatic cancer and fibrosis progression remains unexplored.

Here, we demonstrate that NID2 is associated with poor patient outcomes in pancreatic cancer and that it is predominantly expressed by stromal cells such as CAFs. We show that NID2 reduction regulates matrix deposition and biomechanics in three-dimensional (3D) organotypic models, leading to decreased cancer cell invasion. Furthermore, we show using intravital (in vivo) imaging that NID2 reduction in CAFs causes reduced tumor fibrosis and vascular changes as well as an enhanced response to gemcitabine/Abraxane [nanoparticle albumin–bound paclitaxel (nab-paclitaxel)] chemotherapy. We reveal that reducing NID2 impaired metastasis and improved response to chemotherapy in orthotopic models of pancreatic cancer, revealing NID2 as a potential cotarget in this deadly disease.

## RESULTS

### Temporal MS proteomics of pancreatic GEMMs reveals a dynamic pancreatic cancer matrisome

**Fig. 1.**
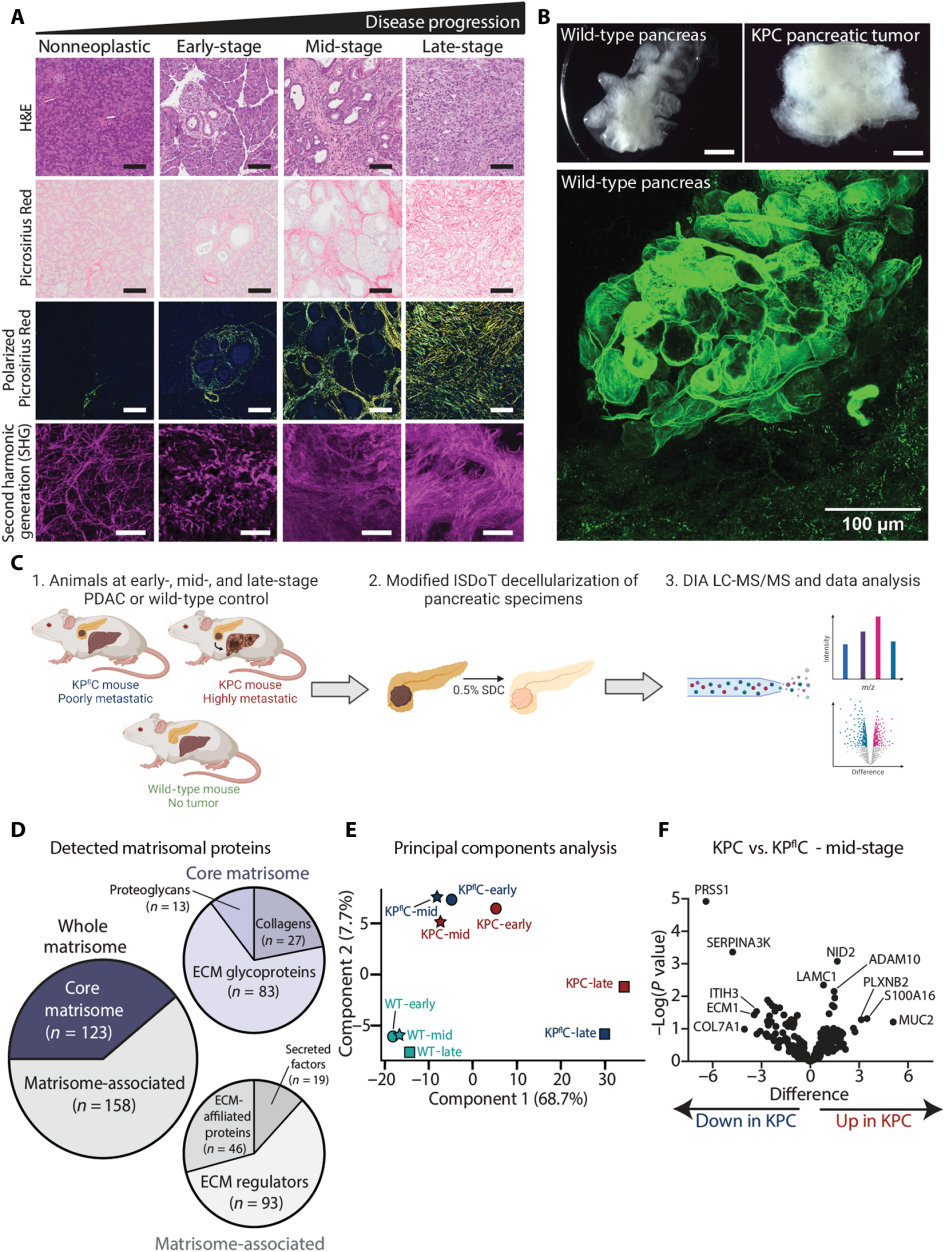
Temporal proteomics of pancreatic cancer GEMMs reveal NID2 as a potential target in aggressive disease. (**A**) Representative images of nonneoplastic pancreas, early-, mid- and late-stage KPC tumors stained for hematoxylin and eosin (H&E) (top row), Picrosirius Red imaged by bright-field (second row) and polarized light (third row; matched to Picrosirius Red images), and SHG imaging (bottom row). Scale bars, 100 μm (H&E and Picrosirius Red bright-field and polarized light) and 25 μm (SHG). (**B**) Representative dark-field images of decellularized WT pancreas (top left) and late-stage KPC specimen (top right). Representative image of collagen IV IF (green) of decellularized WT pancreas (bottom). Scale bars, 500 μm (dark-field) and 100 μm (collagen IV). (**C**) Workflow for matrisome-enriched DIA LC-MS/MS analysis of WT, KP^fl^C, and KPC tumors at early-, mid-, and late-stage time points. *m*/*z*, mass/charge ratio; 0.5% SDC, sodium deoxycholate. (**D**) Classification of matrisome categories detected (*33*). Core matrisome (*n* = 123; purple) and matrisome-associated (*n* = 158; gray). Core matrisome category (purple colors); ECM glycoproteins (*n* = 83), collagens (*n* = 27), and proteoglycans (*n* = 13). Matrisome-associated category (gray colors); ECM regulators (*n* = 93), ECM-affiliated proteins (*n* = 46), and secreted factors (*n* = 19). (**E**) Principal components analysis of WT (green), KP^fl^C (blue), and KPC (red) tumors at early- (circle), mid- (star), and late-stage (square) time points. (**F**) Volcano plot of matrisome proteins comparing KP^fl^C and KPC mid-stage tumors. Two-sample *t* test with FDR = 0.05. *y* axis = −log(*P* value) and *x* axis = difference (fold change). ITIH3, Inter-Alpha-Trypsin Inhibitor Heavy Chain 3; LAMC1, Laminin Subunit Gamma 1; ADAM10, A Disintegrin And Metalloproteinase Domain 10; PLXNB2, Plexin B2; S100A16, S100 Calcium Binding Protein A16; MUC2, Mucin 2; PRSS1, Serine Protease 1; SERPINA3K, serine (or cysteine) peptidase inhibitor, clade A, member 3K; ECM1, Extracellular Matrix Protein 1; COL7A1, Collagen Type VII Alpha 1 Chain. Schematics were created with Biorender.com.

In PDAC, increasing fibrosis accompanies disease progression, with a diverse range of matrisomal proteins deposited and remodeled over time (*4*–*7*). Supporting this, we show that fibrillar collagens via Picrosirius Red staining, coupled with polarized light imaging of the birefringence signal, are increased over disease progression in the autochthonous KPC GEMM of pancreatic cancer (Fig. 1A) (*36*). Similarly, analysis of fibrillar collagens via SHG multiphoton imaging shows that there is an increase in fibrillar collagen over disease progression in the KPC tumors (Fig. 1A). Furthermore, in line with our previous work using ISDoT (*34*, *35*), murine pancreatic tissues were decellularized to remove the cellular content while leaving the 3D ECM architecture and matrisomal components intact (*34*, *35*). Here, we reveal global perturbations in ECM organization and architecture in KPC tumors compared to WT mouse pancreas (Fig. 1B and fig. S1A for representative decellularized WT mouse pancreas and late-stage KPC tumor). Furthermore, we reveal the detailed architecture of the WT pancreas, shown via collagen IV immunofluorescence (IF) (Fig. 1B, fig. S1A, and movie S1).

We and others have shown that the cancer cell genotype can have an influence on tumor fibrosis and metastatic spread using autochthonous pancreatic cancer GEMMs (*16*, *28*, *46*). The KP^fl^C mouse model, in which pancreatic cancer cells have lost p53 expression, has lower fibrosis (*28*) and rates of metastasis (*37*) compared to the KPC mouse model, which harbors a gain-of-function R172H mutation in p53. Considering this, we aimed to characterize the fibrotic changes in pancreatic cancer over time in the highly metastatic KPC model and the poorly metastatic KP^fl^C model to reveal matrix proteins that may be associated with metastatic pancreatic cancer using a proteomics approach.

We collected pancreatic tissue from KPC, KP^fl^C, and age-matched WT control mice at early- (41 to 53 days), mid- (70 to 84 days), and late-stage (99 to 212 days) disease to capture the different disease stages in each mouse model in an unbiased manner (Fig. 1C and table S1) (*36*, *37*). These tissues were enriched for matrisomal proteins in line with our previous ISDoT decellularization work (*34*, *35*, *47*) and analyzed using DIA LC-MS/MS (Fig. 1C). Across the nine age- and genotype-dependent conditions (WT-early, WT-mid, WT-late, KP^fl^C-early, KP^fl^C-mid, KP^fl^C-late, KPC-early, KPC-mid, and KPC-late), 44 specimens were analyzed (*n* = 4 to 5 per condition) (table S1). A total of 281 matrisomal proteins were detected across the whole proteomic dataset, with 123 proteins categorized as “core matrisome” and 158 proteins categorized as “matrisome-associated” (Fig. 1D and table S2), in line with the Naba *et al.* (*33*) matrisome classification. Within the core matrisome category, we detected 83 ECM glycoproteins, 27 collagens, and 13 proteoglycans (Fig. 1D and table S2). In the matrisome-associated category, 93 ECM regulators, 46 ECM-affiliated proteins, and 19 secreted factors were detected (Fig. 1D and table S2). Encouragingly, our dataset was comparatively concordant with the Barrett *et al.* (*48*) and Tian *et al.* (*49*) mouse pancreatic cancer proteome datasets, with 91 of 281 and 229 of 281 common detected proteins, respectively (fig. S1C and table S2). Both Barrett *et al.* (*48*) and Tian *et al.* (*49*) used a fractionation methodology to enrich for matrix proteins from the tumors of pancreatic cancer GEMMs, namely, the KTC (*Ptf1a-Cre; LSL-Kras^G12D/+^; Tgfbr2^fl/+^*) mouse model (*48*) or KC (*Pdx1-Cre; LSL-K-ras^G12D/+^*) and KPC pancreatic mouse models (*49*). We detected 41 unique proteins that were not detected in the datasets of Barrett *et al.* (*48*) and Tian *et al.* (*49*) (fig. S1C and table S2), highlighting the value of our tissue decellularization approach as well as the KPC tumor versus KP^fl^C tumor comparison in detecting matrisomal tumor proteins relevant to pancreatic cancer progression and metastasis.

Principal components analysis shows that the global matrisome of the WT pancreas does not substantially change over time after 6 weeks of age, with WT-early, WT-mid, and WT-late clustering together in the bottom left corner (Fig. 1E). However, across the largest component (principal component 1; 68.7%), a considerable shift in the abundance of matrisomal proteins dependent on disease stage for both KP^fl^C and KPC genotypes was observed, with KP^fl^C/KPC-early and KP^fl^C/KPC-mid clustering together toward the left and KP^fl^C/KPC-late clustering to the right of principal component 1 (Fig. 1E). Furthermore, two-sample *t* tests [false discovery rate (FDR) = 0.05] revealed that 170 matrisomal proteins were differentially abundant in terms of age or genotype across the dataset (fig. S2 and table S3). We observed several known cancer-associated matrix proteins up-regulated in the KPC and KP^fl^C late-stage tumors compared to age-matched WT control (table S3). These include fibronectin (*50*), tenascin C (*51*), lysyl oxidase and lysyl oxidase–like 1 (LOX/LOXL1) (*32*, *52*), collagen type XII alpha 1 (COL12A1) (*47*), and periostin (*53*). Furthermore, we identified several matrix proteins such as elastin microfibril interfacer 2 (*54*) and nephronectin (*55*), which have been relatively unexplored in pancreatic cancer but have roles in other solid tumors and therefore warrant further investigation in the future (table S3). Similarly, prolyl 4-hydroxylase subunit alpha 1 (P4HA1) and procollagen C-endopeptidase enhancer (PCOLCE) were identified as up-regulated in the cancer GEMMs (table S3), which both have known roles in collagen modification and folding (*56*) and therefore could be involved in promoting desmoplasia in PDAC development. Although there were considerably more differentially abundant proteins between WT and KP^fl^C and WT and KPC across all time points (fig. S2 and table S3), we focused on matrisomal proteins that were differentially abundant in the highly metastatic KPC model compared to the poorly metastatic KP^fl^C model, to identify matrisomal proteins associated with metastatic spread. Here, we identified proteins such as protease serine 1 (PRSS1) and serine peptidase inhibitor, clade A, member 3K (SERPINA3K) to be lower in abundance in the highly metastatic KPC tumors compared to the poorly metastatic KP^fl^C tumors (Fig. 1F and table S3). Critically, we identified NID2 as more abundant in the mid-stage KPC tumors compared to KP^fl^C tumors of the same stage (Fig. 1F and table S3). NID2 is a BM protein and is considered a linking glycoprotein between laminin and collagen IV networks at the BM (*38*, *39*). High NID2 expression is associated with several malignancies (*40*–*45*), but its role in pancreatic cancer progression and metastasis had been relatively unexplored. It was also unknown whether NID2 had a role in promoting a desmoplastic pancreatic stroma, in addition to its known roles in BM biology. Considering this, we decided to further investigate the role of NID2 in pancreatic cancer.

### NID2 expression is associated with poor patient outcomes in PDAC and is enhanced in CAFs

**Fig. 2.**
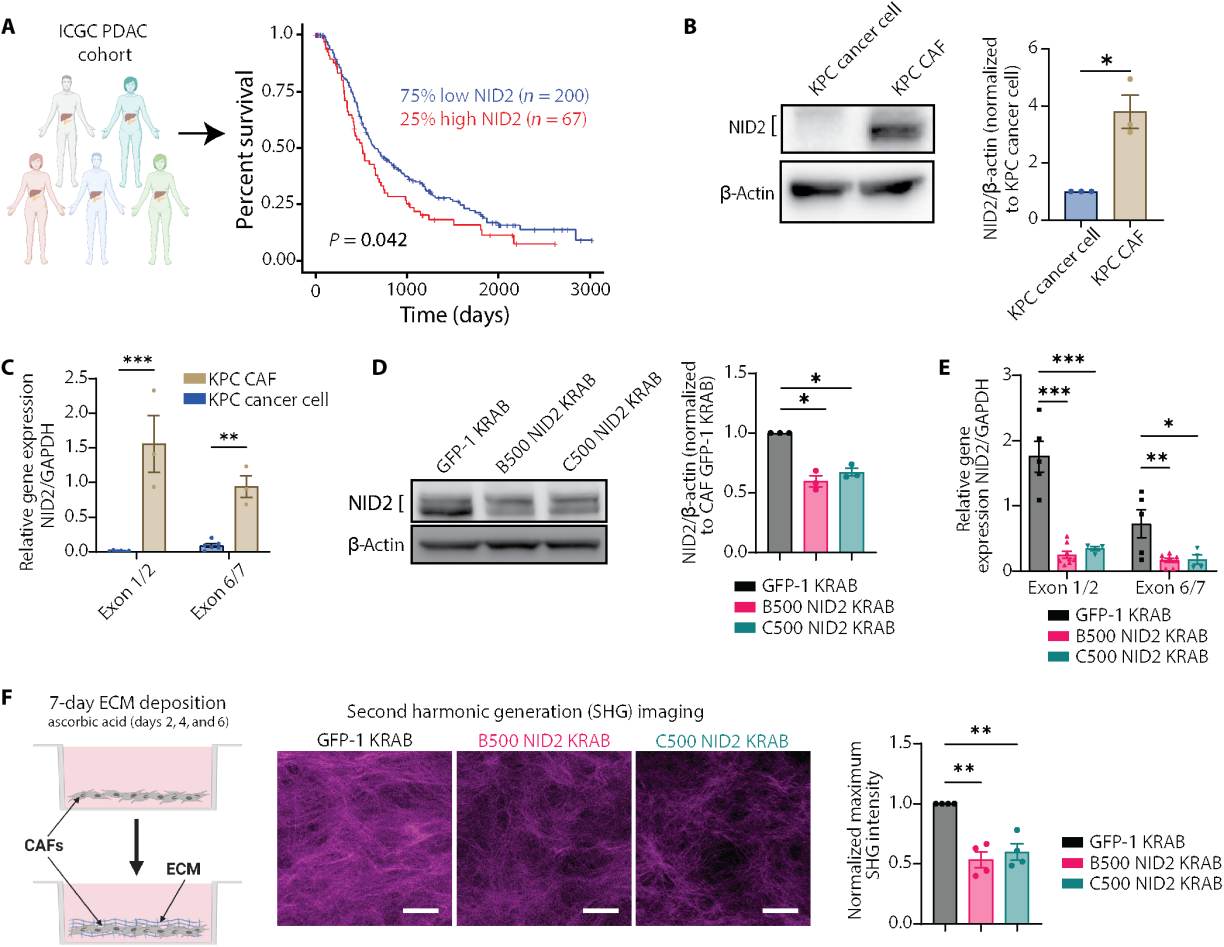
NID2 expression is highly expressed in CAFs and can be depleted via CRISPRi. (**A**) Kaplan-Meier analysis of ICGC PDAC cohort (*n* = 267) based on *NID2* mRNA. High *NID2* (red; *n* = 67) compared with low *NID2* (blue; *n* = 200). Log-rank test, *P* = 0.042. (**B**) NID2 Western blot for KPC cancer cells and KPC CAFs with quantification. One-sample *t* test, *P < 0.05. *n* = 3 independent repeats. (**C**) RT-qPCR *NID2* for KPC cancer cells and KPC CAFs, exon 1/2 and exon 6/7. Two-way analysis of variance (ANOVA) with Sidak’s test, **P < 0.01 and ***P < 0.001. *n* = 6 repeats for KPC cancer cells and *n* = 3 repeats for CAFs, in triplicate. (**D**) NID2 Western blot for GFP-1 KRAB, B500 NID2 KRAB, and C500 NID2 KRAB CAFs with quantification. One-sample *t* test, *P < 0.05. *n* = 3 independent repeats. (**E**) RT-qPCR *NID2* for GFP-1 KRAB, B500 NID2 KRAB, and C500 NID2 KRAB CAFs, exon 1/2 and exon 6/7. Two-way ANOVA with Dunnett’s test, *P < 0.05, **P < 0.01, and ***P < 0.001. *n* = 5 GFP-1 KRAB repeats, *n* = 8 B500 NID2 KRAB repeats, and *n* = 4 C500 NID2 KRAB repeats, in triplicate. (**F**) CAF cell-derived matrix (CDM) assay. Representative regions of interest (ROIs) of SHG maximum projection for GFP-1 KRAB, B500 NID2 KRAB, and C500 NID2 KRAB CAF CDMs (day 7). Scale bars, 100 μm. Quantification of normalized SHG maximum intensity. One-sample *t* test, **P < 0.01. *n* = 4 repeats all CAF lines, with 4 to 8 ROIs per well in triplicate. All data represented as means ± SEM. Schematics were created with Biorender.com.

To assess the clinical relevance of NID2 in pancreatic cancer, we initially interrogated The Cancer Genome Atlas (TCGA) PDAC cohort (*n* = 378) via OncoDB (*57*) (fig. S3A). We show that *NID2* mRNA expression was increased in PDAC specimens compared to nonneoplastic pancreatic tissue via this bulk RNA sequencing (RNA-seq) dataset (fig. S3A) (*57*). Furthermore, Kaplan-Meier analysis of *NID2* mRNA expression and patient survival in the International Cancer Genome Consortium (ICGC) PDAC cohort (*n* = 267) reveals that high *NID2* expression is associated with poorer overall survival in patients with pancreatic cancer (Fig. 2A). Further analysis using the molecular classification, previously described by Moffitt *et al.* (*58*), identified that high *NID2* expression is associated with poorer patient survival in “basal-like” tumors (*P* = 0.032), but not for “classical” PDAC tumors (fig. S3, B and C, and table S4). Patients with basal-like tumors have worse outcomes compared to classical tumors (*58*), indicating that high *NID2* is associated with the most aggressive pancreatic cancer cases.

To assess the cellular sources of NID2 in the pancreatic tumor milieu, we interrogated publicly available single-cell RNA-seq (scRNA-seq) datasets of both murine KPC tumors and human PDAC (fig. S3, D to G) (*8*, *59*). In KPC tumors, *NID2* expression was most abundant in CAFs and pericytes (fig. S3D) (*8*). Similarly, in human PDAC, stromal cells such as fibroblasts, stellate cells, and endothelial cells were the main sources of *NID2* (fig. S3E) (*59*). Furthermore, *NID2* is overexpressed in human PDAC specimens compared to nonneoplastic pancreas controls in this scRNA-seq dataset (fig. S3, F and G) (*59*), supporting the bulk OncoDB TCGA RNA-seq data (fig. S3A) (*57*). To validate the scRNA-seq data (*8*, *59*), we assessed NID2 staining via IF in the stromal regions of KPC tumors at early-, mid-, and late-stage diseases, with colocalization with perivascular stromal cells (CD146) (*60*) and endothelial cells (CD31) evident (fig. S4).

Considering the clinical data linking high *NID2* expression with poor pancreatic cancer patient outcomes, we aimed to assess the functional role of NID2 using cancer cells and CAFs derived from the KPC mouse model (*28*, *36*). We found that NID2 was highly expressed in the KPC CAFs compared to the KPC cancer cells via Western blot (Fig. 2B) and reverse transcription quantitative polymerase chain reaction (RT-qPCR) (Fig. 2C), in line with the scRNA-seq (fig. S3, D to G). From here, we generated two NID2 knockdown CAF lines using two individual CRISPR interference (CRISPRi) guide RNA Krüppel-associated box (KRAB) constructs, B500 NID2 KRAB, and C500 NID2 KRAB. For a nontargeting control, we used a green fluorescent protein–1 (GFP-1) KRAB CRISPRi construct as the *Mus musculus* genome does not endogenously contain GFP-1. We observed a partial knockdown of NID2 in the CAF B500 NID2 KRAB and CAF C500 NID2 KRAB lines compared to CAF GFP-1 KRAB control via Western blot and RT-qPCR (Fig. 2, D and E).

This knockdown was further confirmed by RNA-seq of the three CRISPRi CAF lines, where *NID2* expression was down-regulated in the B500 NID2 KRAB and C500 NID2 KRAB lines compared to GFP-1 KRAB control (fig. S5 and table S5). Considering that NID2 is a BM matrix protein (*38*), we also assessed whether reducing NID2 expression affected the expression of other ECM genes by comparing our RNA-seq dataset against genes that constitute the matrisome (fig. S6, A and B, and table S6) (*33*) and BM (fig. S6C and table S6) (*45*). For the matrisomal genes, *K*-means clustering revealed three distinct clusters of genes (fig. S6A). Cluster 1 contains the most down-regulated genes in the NID2 knockdown setting, whereas clusters 2 and 3 showed little or no change (fig. S6A). Differential expression analysis revealed 13 genes that were down-regulated alongside NID2 such as *MFAP5*, *FGF5*, *COL27A*, and *MUC16*, while *HGF* was up-regulated with NID2 loss (fig. S6B and table S6). For the BM genes, *K*-means clustering revealed three distinct clusters of genes (fig. S6C). Clusters 1 and 3 showed little or no change in response to NID2 depletion (fig. S6C). Meanwhile, cluster 2 contains down-regulated genes in the response to NID2 knockdown (including *NID2* and key BM proteins *COL4A1*, *COL4A2*, and *LAMA5*) (fig. S6C).

Considering these data, we then assessed the effect of lowered NID2 expression on ECM deposition using cell-derived matrices (CDMs) (Fig. 2F) (*61*, *62*). Multiphoton SHG imaging demonstrated a reduction in SHG peak signal intensity (Fig. 2F); however, no differences in collagen production or *COL1A1* mRNA were observed (fig. S7, A and B). We also assessed the GFP-1 KRAB, B500 NID2 KRAB, and C500 NID2 KRAB CAF lines for stress fibers via F-actin staining (fig. S8) as well as phosphorylated myosin light chain 2 S19 (pMLC2) and phosphorylated myosin phosphatase target subunit 1 T696 (pMYPT1) via IF and Western blot; however, no differences between the lines were observed (fig. S9, A to E). Overall, considering the RNA-seq analysis as well as phenotypic characterization and functional in vitro CDM assays, we show that NID2 manipulation in CAFs may perturb some aspects of ECM production and matrix remodeling.

### NID2 reduction modulates matrix organization and biomechanics while reducing cancer cell invasion in 3D organotypic matrices

**Fig. 3.**
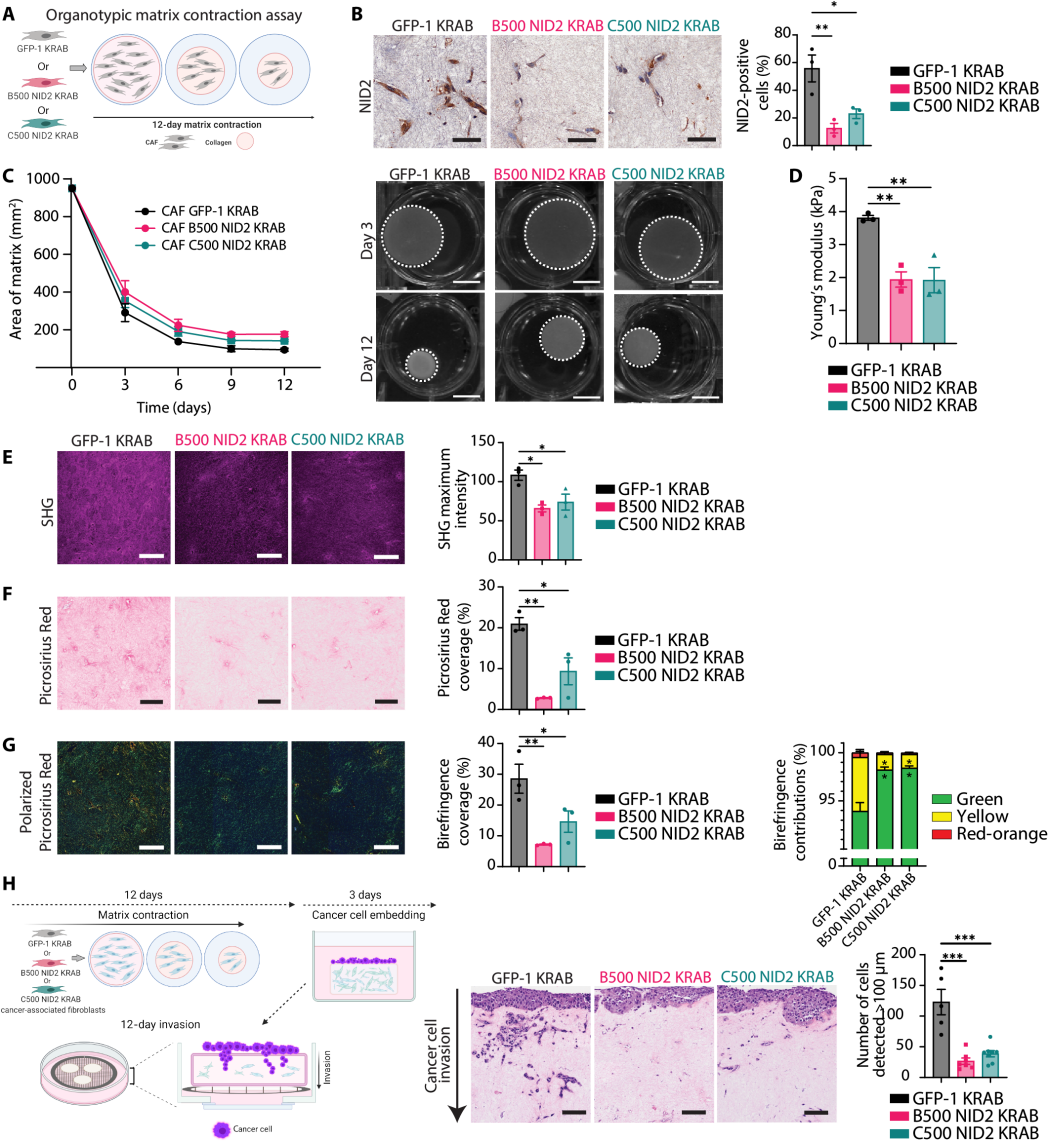
NID2 reduction in CAFs reduces biomechanical properties in organotypic matrices, leading to altered cancer cell invasion. (**A**) 3D organotypic contraction assay with CRISPRi CAFs. (**B**) Representative ROIs of NID2 IHC with NID2-positive cell (%) quantification. One-way ANOVA with Dunnett’s test, *P < 0.05 and **P < 0.01. *n* = 3 repeats, in triplicate. Scale bars, 50 μm. (**C**) Quantification at days 3, 6, 9, and 12 and representative images of matrix contraction (in square millimeter) at days 3 and 12. Scale bars, 1 cm. (**D**) Young’s modulus (in kilopascals) of matrices on day 12. One-way ANOVA with Dunnett’s test, **P < 0.01. *n* = 3 repeats, in triplicate. (**E**) Representative SHG maximum intensity with quantification. One-way ANOVA with Dunnett’s test, *P < 0.05. *n* = 3 repeats, in triplicate. Scale bars, 100 μm. (**F**) Representative ROIs of Picrosirius Red with coverage (%) quantification. One-way ANOVA with Dunnett’s test, *P < 0.05 and **P < 0.01. *n* = 3 repeats, in triplicate. Scale bars, 100 μm. (**G**) Representative ROIs of polarized light Picrosirius Red (matched to Picrosirius Red ROIs) with coverage (%) quantification. One-way ANOVA with Dunnett’s test; green, yellow, and red-orange birefringence proportion quantification. Two-way ANOVA with Dunnett’s test, *P < 0.05 and **P < 0.01. *n* = 3 repeats, in triplicate. Scale bars, 100 μm. (**H**) Organotypic invasion assay. Representative H&E ROIs of cancer cell invasion with quantification. One-way ANOVA with Dunnett’s test, ***P < 0.001. *n* = 5 GFP-1 KRAB, *n* = 7 B500 NID2 KRAB, and *n* = 8 C500 NID2 KRAB repeats, in triplicate. Scale bars, 100 μm. All data represented as means ± SEM. Schematics were created with Biorender.com.

Considering that NID2 knockdown in CAFs reduces SHG signal in CDMs (Fig. 2F) and alters the expression of several matrisomal genes (fig. S6), we next assessed the 3D contractile properties of CAFs with NID2 reduction in organotypic matrices (Fig. 3A) (*15*, *28*, *29*, *63*, *64*). Here, CAFs are seeded in 3D fibrillar collagen matrices and cultured for 12 days. Over this time, the CAFs contract the matrix and induce remodeling and cross-linking of the 3D collagen substrate. We first confirmed that NID2 expression was reduced in the CAF B500 NID2 KRAB and CAF C500 NID2 KRAB matrices compared to CAF GFP-1 KRAB via immunohistochemistry (IHC) in this 3D setting (Fig. 3B). This reduction in NID2 did not result in changes in NID1 expression nor did it result in changes in proliferation (Ki-67) and apoptosis [cleaved caspase 3 (CC3)], shown via IHC (fig. S10, A to C). We found that NID2 reduction in the 3D organotypic matrices did not cause considerable changes in organotypic matrix contraction compared to control (Fig. 3C and fig. S10D), in line with our pMLC2 and pMYPT1 IF and Western blot data (fig. S9). However, the NID2-depleted matrices were softer than control, as shown by biomechanical compression analysis (Fig. 3D). Furthermore, analysis of the organotypic matrix using scanning electron microscopy confirmed a looser matrix network under the NID2-depleted conditions (fig. S10E). To understand this further, we performed MS proteomics on the GFP-1 KRAB, B500 NID2 KRAB, and C500 NID2 KRAB CAF lines (fig. S11 and table S7). We found that in the NID2 depleted setting, there are several proteomic changes including less LOX abundance (fig. S11 and table S7), a collagen cross-linking enzyme (*56*). These data collectively indicate that NID2 may be associated with tissue stiffening and matrix organization, which occurs during pancreatic tumor development (*4*–*7*).

In support of the CDM and MS proteomic data, SHG multiphoton imaging revealed that NID2 knockdown causes a reduction in fibrillar collagen signal in both NID2 knockdown lines (Fig. 3E). Moreover, Picrosirius Red coverage was reduced in the NID2 knockdown matrices compared to control (Fig. 3F). Using polarized light imaging of the Picrosirius Red signal, we observed a reduction in birefringence coverage in the CAF B500 NID2 KRAB and CAF C500 NID2 KRAB matrices compared to control (Fig. 3G). When assessing the contribution of high (red-orange), intermediate (yellow), and low (green) birefringence fibers to this signal, we also observed an increase in the proportion of low birefringent collagen fibers (green) in the CAF B500 NID2 KRAB and CAF C500 NID2 KRAB organotypic matrices compared to control (Fig. 3G) (*15*, *28*, *29*, *32*, *64*). There was also a corresponding reduction in the proportion of intermediate birefringent collagen (yellow) under the NID2-depleted conditions compared to control (Fig. 3G). Given these effects, we then assessed whether reducing NID2 in CAFs was sufficient to alter tumor cell invasion in this highly aggressive disease. To do this, we seeded the KPC cancer cells on top of CAF-contracted organotypic matrices (GFP-1 KRAB, B500 NID2 KRAB, or C500 NID2 KRAB) and allowed them to attach and proliferate for 3 days, followed by a 12-day period of invasion via an air-liquid interface and chemotactic gradient (Fig. 3H), as previously achieved (*15*, *28*, *29*, *63*, *64*). These 3D organotypic matrix invasion assays revealed a reduction in the ability of the KPC cancer cells to invade into NID2 knockdown organotypic matrices compared to control (Fig. 3H). In the GFP-1 KRAB setting, more KPC cancer cells were able to invade to a greater depth within the 3D matrix (>100 μm), whereas in the B500 NID2 KRAB and C500 NID2 KRAB organotypic matrices, cancer cell invasion was reduced, with less cells able to invade beyond 100 μm (Fig. 3H). Given that our NID2-reduced matrices were softer than control (Fig. 3D), these invasion data are in line with previous work demonstrating that a softer microenvironment can result in a less invasive phenotype in cancer cells (*15*, *29*, *65*–*67*). Overall, these data demonstrate that reducing NID2 in the stromal compartment can alter matrix organization, resulting in an impairment in pancreatic cancer invasion in 3D organotypic settings.

### NID2 targeting improves response to gemcitabine/Abraxane chemotherapy in vivo

**Fig. 4.**
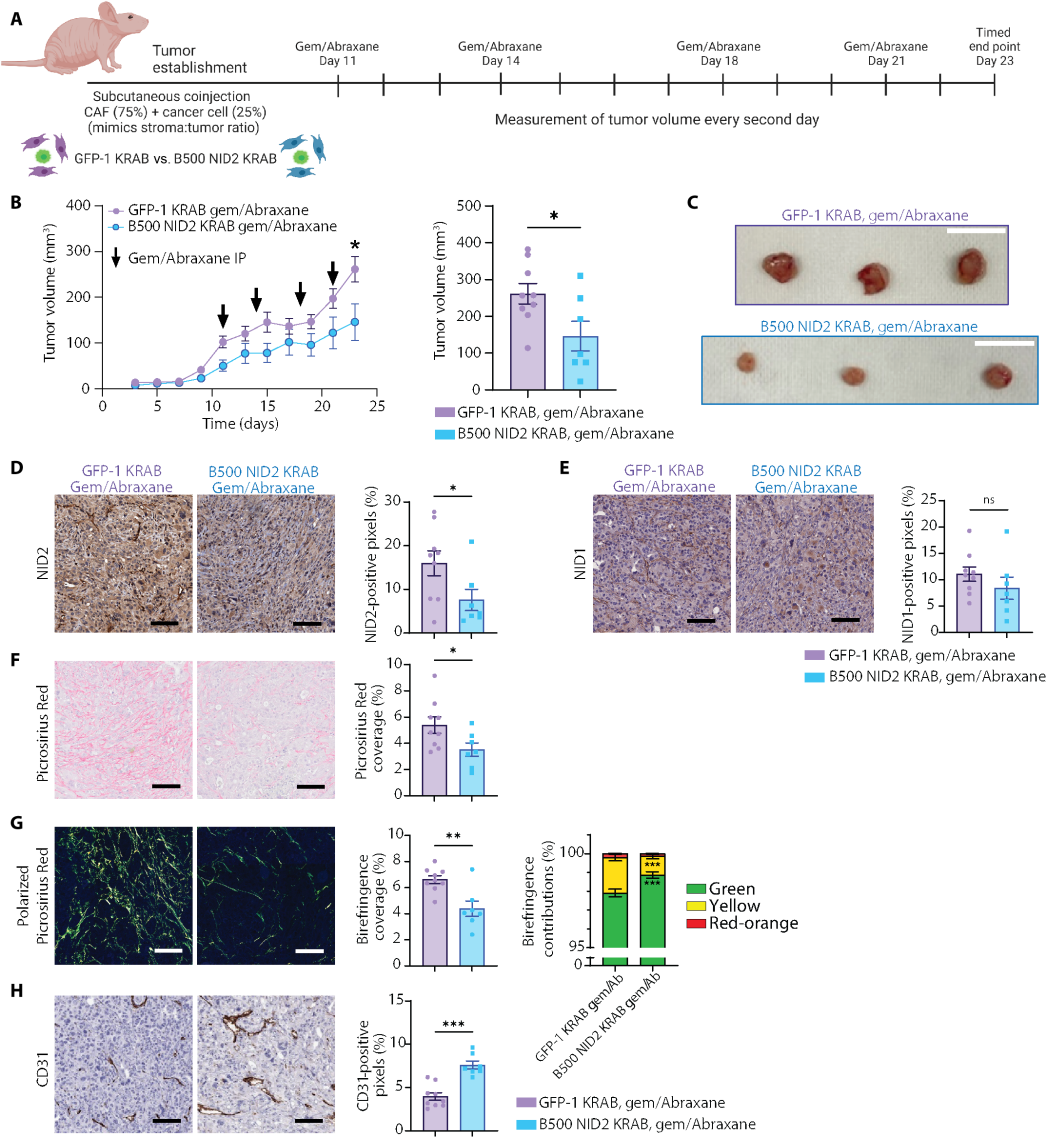
NID2 reduction in CAFs leads to reduced tumor growth and fibrosis in subcutaneous model under gemcitabine/Abraxane treatment. (**A**) Subcutaneous coinjection experiment using GFP-1 KRAB or B500 NID2 KRAB CAFs (75%) with cancer cells (25%). Mice were treated twice weekly with gemcitabine/Abraxane, beginning day 11. (**B**) Quantification of GFP-1 KRAB (purple) and B500 NID2 KRAB (blue) tumor growth (in cubic millimeters) over time and at day 23. Welch’s *t* test, *P < 0.05. *n* = 9 GFP-1 KRAB mice and *n* = 7 B500 NID2 KRAB mice. IP, intraperitoneal injection. (**C**) Representative images of day 23 tumors. Scale bars, 2 cm. (**D**) Representative ROIs of NID2 IHC with quantification of positive pixels (%). Mann-Whitney test, *P < 0.05. Scale bars, 100 μm. (**E**) Representative ROIs of NID1 IHC with quantification of positive pixels (%). Welch’s *t* test, ns: *P* > 0.05. Scale bars, 100 μm. (**F**) Representative ROIs of Picrosirius Red with quantification of coverage (%). Welch’s *t* test, *P < 0.05. Scale bars, 100 μm. (**G**) Representative ROIs of polarized light Picrosirius Red with quantification of coverage (%). Welch’s *t* test, **P < 0.01. Quantification of proportions of green, yellow, and red-orange birefringence. Two-way ANOVA with Sidak’s test, ***P < 0.001 for green and yellow birefringence. Scale bars, 100 μm. (**H**) Representative ROIs of CD31 IHC with quantification of positive pixels (%). Welch’s *t* test, ***P < 0.001. Scale bars, 100 μm. All data represented as means ± SEM. Schematics were created with Biorender.com.

Guided by our organotypic data, we next assessed the effects of CAF-derived NID2 on tumor growth in vivo in the context of standard-of-care gemcitabine/Abraxane (nab-paclitaxel) chemotherapy (*2*). As the B500 NID2 KRAB and C500 NID2 KRAB lines had comparable effects on matrix remodeling (Figs. 2 and 3), we performed subsequent in vivo assessments herein with B500 NID2 KRAB CAFs. We coinjected KPC cancer cells with CAFs (GFP KRAB or B500 NID2 KRAB) into the flank of nude BALB/c-Fox1nuAusb mice subcutaneously (Fig. 4A and fig. S12A), as previously achieved (*28*). We used a 1:3 ratio of KPC cancer cells to CAFs to recapitulate the highly desmoplastic stromal tissue and low cancer cellularity typically observed in pancreatic tumors (*28*). For this experiment, all mice were culled in the vehicle or chemotherapy-treated arms when the first mouse within that arm reached a study end point (day 19 for vehicle arms and day 23 for chemotherapy arms; fig. S12A and Fig. 4A, respectively).

In the vehicle arms of the subcutaneous experiment, we observed no differences in tumor volume between CAF GFP-1 KRAB compared to CAF B500 NID2 KRAB (fig. S12B). However, we observed a decrease in tumor volumes in the context of gemcitabine/Abraxane treatment (Fig. 4, B and C). We confirmed that NID2 expression was decreased in the CAF B500 NID2 tumors compared to control, as shown via IHC (Fig. 4D and fig. S12C). For both the vehicle and chemotherapy-treated arms, NID1 levels were unchanged between GFP-1 KRAB and B500 NID2 KRAB tumors (Fig. 4E and fig. S12D). In support of our organotypic in vitro studies, we observed a reduction in Picrosirius Red coverage in the B500 NID2 KRAB tumors in both the vehicle and gemcitabine/Abraxane arms of the study compared to control (Fig. 4F and fig. S12E). For birefringence coverage, there was also a decrease in the birefringence signal in the NID2-depleted tumors compared to control in the chemotherapy arm of the study (Fig. 4G), but not the vehicle arm (fig. S12F). Moreover, the proportion of green birefringence collagen fibers was higher in the CAF B500 NID2 KRAB tumors, with a subsequent decrease in yellow intermediate collagen fibers, as shown via polarized light imaging of the Picrosirius Red–stained sections (Fig. 4G). Considering that NID2 is a BM stabilizing protein (*38*, *39*), we also assessed whether collagen IV and laminin expressions (major components of the BM) were perturbed via IF; however, no changes in signal intensity were observed (fig. S12, G and H). In addition, blood vessel coverage (examined via CD31 IHC) was increased in the CAF B500 NID2 KRAB tumors compared to control in both the vehicle and chemotherapy-treated setting (Fig. 4H and fig. S12I). This indicates that NID2 reduction may indirectly affect tumor vascularization (*19*, *20*), due to the reduction in tumor fibrosis observed via reduced Picrosirius Red intensity and changes in birefringence proportions observed with polarized light imaging (*15*, *28*, *29*, *32*, *64*). This change in vasculature could also be regulated by various soluble factors. To assess this further, we cross-referenced our RNA-seq dataset with VerSeDa, a database for secreted factors by both classical and nonclassical mechanisms (*68*). Here, we found that there were 10 genes that were both differentially expressed and predicted to be secreted (*68*), which could influence vessel biology (table S5). This could, in part, explain why gemcitabine/Abraxane was more effective at reducing tumor burden in the NID2-depleted tumors in this subcutaneous in vivo model.

**Fig. 5.**
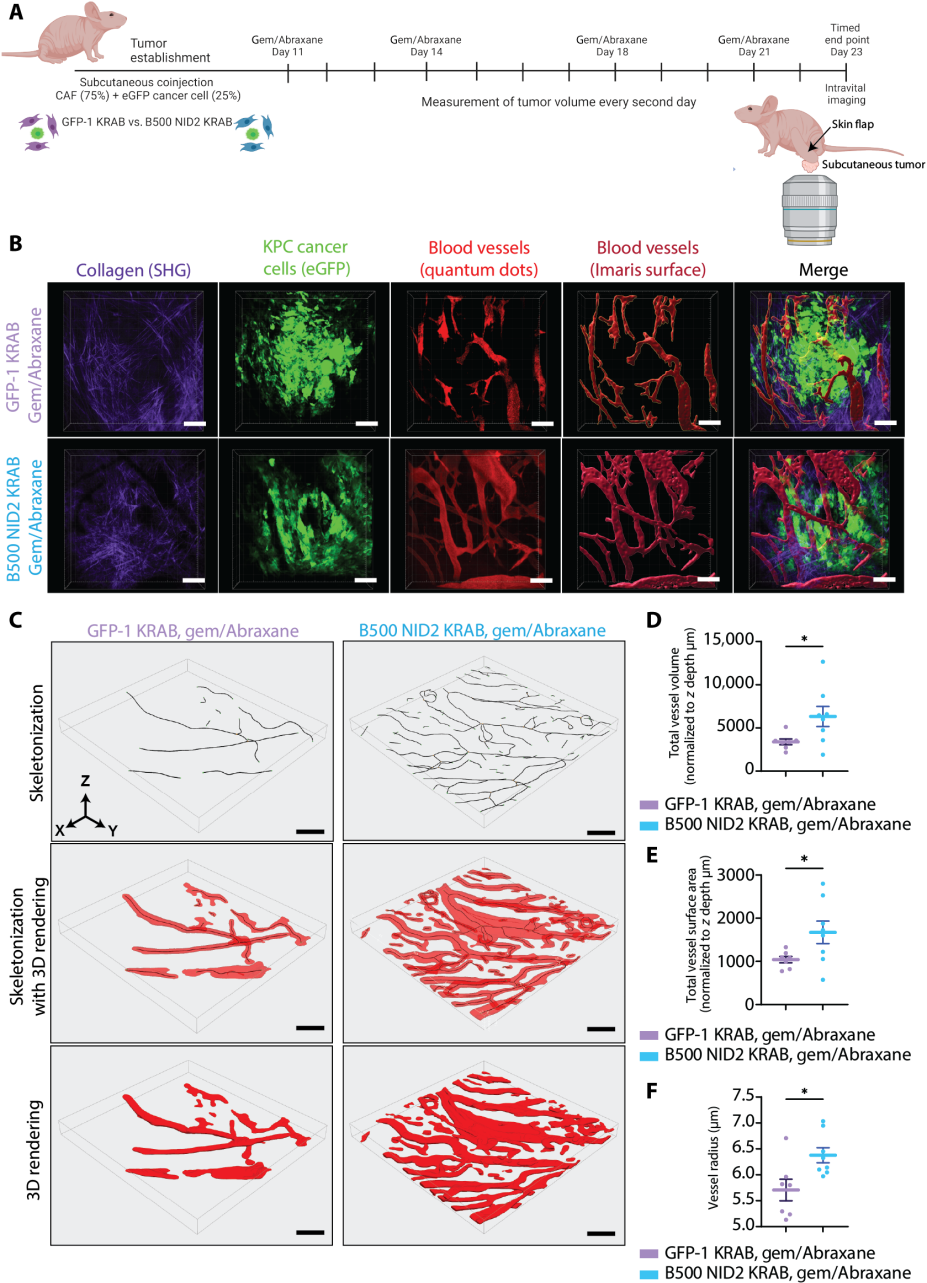
NID2 reduction in CAFs leads to vascular changes in subcutaneous model under gemcitabine/Abraxane treatment shown via intravital imaging and quantum dots. (**A**) Subcutaneous coinjection intravital imaging experiment using GFP-1 KRAB or B500 NID2 KRAB CAFs (75%) with eGFP-tagged cancer cells (25%). Quantum dots injected via tail vein. Mice were treated twice weekly with gemcitabine/Abraxane, beginning day 11. *n* = 7 GFP-1 KRAB mice and *n* = 8 B500 NID2 KRAB mice. (**B**) Representative images for GFP-1 KRAB and B500 NID2 KRAB tumors imaged live via multiphoton intravital microscopy. SHG of fibrillar collagen (purple), eGFP cancer cells (green), vasculature (quantum dots; red), Imaris surface of blood vessels (red), and merged image. Scale bars, 100 μm. (**C**) Representative images of the vasculature of GFP-1 KRAB and B500 NID2 KRAB analyzed using VesselVio. Vessel skeletonization (black skeleton; top), skeletonization with 3D vessel rendering (black skeleton with red rendering; middle), and 3D vessel rendering alone (red rendering; bottom). Scale bars, 100 μm. (**D**) Quantification of total vessel volume (normalized to *z* depth of image in micrometers). Welch’s *t* test, *P < 0.05. (**E**) Quantification of total vessel surface area (normalized to *z* depth of image in micrometers). Welch’s *t* test, *P < 0.05. (**F**) Quantification of mean vessel radius (in micrometers) for GFP-1 KRAB (purple) and B500 NID2 KRAB (blue) live tumors. Welch’s *t* test, *P < 0.05. All data represented as means ± SEM. Schematics were created with Biorender.com.

On the basis of these in vivo findings, we then aimed to further examine the live vasculature characteristics of tumors in the context of a NID2-depleted TME using intravital imaging (*15*, *69*–*72*). As before, we performed a subcutaneous coinjection of enhanced GFP (eGFP)–expressing KPC cancer cells with CAFs (GFP KRAB or B500 NID2 KRAB) in a 1:3 ratio into the flank of nude BALB/c-Fox1nuAusb mice (Fig. 5A). Mice were treated with gemcitabine/Abraxane (Fig. 5A), mirroring our previous subcutaneous study (Fig. 4). We confirmed a reduction in tumor volume at day 23 end point (fig. S13A), in line with our previous subcutaneous gemcitabine/Abraxane study in Fig. 4. On day 23, we intravenously injected the mice with quantum dots (Qtracker 655 Vascular Label) and exposed the live tumor via a “skin flap” surgical technique, followed by intravital imaging of these tumors, as previously achieved (see movies S2 and S3 for representative live blood flow time-lapse images and movie S4 for a representative z-stack showing the detailed architecture of the live tumor including eGFP-tagged cancer cells in green, quantum dot–filled vessels in red, and fibrillar collagens via SHG in magenta) (*15*, *69*–*72*). 3D rendered z-stacks of live pancreatic tumors perfused with quantum dots were used to assess vessel network architecture (Fig. 5, B and C, and fig. S13, B and C). We extracted 3D vessel networks of the live tumor images, which could then be skeletonized, segmented, and analyzed for vessel architecture parameters using VesselVio (see fig. S13C for workflow) (*73*). Using this vessel analysis workflow, we observed an increase in total vessel volume, total vessel surface area, and mean vessel radius in the B500 NID2 tumors compared to GFP-1 KRAB control (Fig. 5, B and C, quantified in D to F). Collectively, these intravital imaging data confirmed increased vascular patency in the NID2-depleted setting, which could, in part, explain the reduction in tumor volume upon gemcitabine/Abraxane treatment for the B500 NID2 KRAB tumors, where chemotherapy may reach tumors more efficiently via a more patent 3D blood vessel network.

### NID2 targeting reduces primary tumor growth and metastasis in combination with gemcitabine/Abraxane in orthotopic models of PDAC

**Fig. 6.**
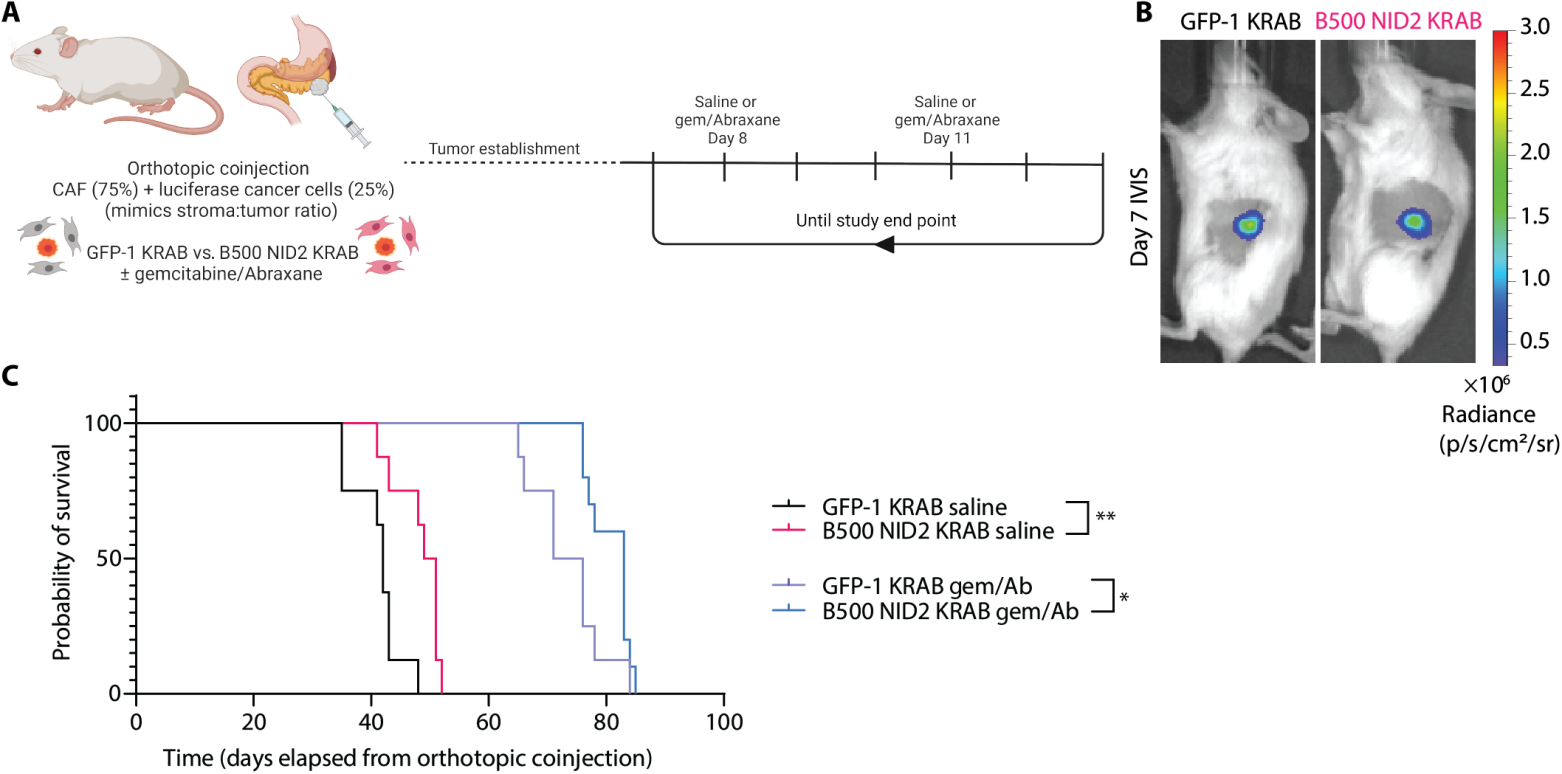
NID2 reduction in CAFs leads to increased median survival in an orthotopic coinjection model. (**A**) Orthotopic coinjection survival experiment using GFP-1 KRAB or B500 NID2 KRAB CAFs (75%) with luciferase cancer cells (25%). Mice were treated twice weekly with gemcitabine/Abraxane upon detectable IVIS signal. (**B**) Representative IVIS signal image on day 7 of mouse bearing GFP-1 KRAB tumor and B500 NID2 KRAB tumor. IVIS signal with a Fstop1, exp30s. Color scale is radiance. (**C**) Kaplan-Meier survival curves of mice bearing orthotopic tumors with GFP-1 KRAB or B500 NID2 KRAB treated with vehicle (saline) or gemcitabine/Abraxane. *n* = 8 GFP-1 KRAB saline mice (median survival, 42 days; black), *n* = 8 B500 NID2 KRAB saline mice (median survival, 50 days; pink), *n* = 8 GFP-1 KRAB gemcitabine/Abraxane mice (median survival, 73.5 days; purple), and *n* = 10 B500 NID2 KRAB gemcitabine/Abraxane mice (median survival, 83 days; blue). Kaplan-Meier curves (GFP-1 KRAB saline compared to B500 NID2 KRAB saline and GFP-1 KRAB gemcitabine/Abraxane compared to B500 NID2 KRAB gemcitabine/Abraxane) were compared with a log-rank Mantel-Cox test. *P < 0.05 and **P < 0.01. Schematics were created with Biorender.com.

Considering our promising results in the subcutaneous study, we then explored the role of CAF-derived NID2 in an orthotopic (intrapancreatic) model. To do this, we coinjected a 1:3 ratio of luciferase-expressing KPC cancer cells and CAFs (GFP KRAB or B500 NID2 KRAB) into the pancreas of NOD.Cg-Prkdc^scid^IL2rg^tm1Wjl^/SzAusb (NSG) mice, as previously achieved (Fig. 6A) (*15*, *28*, *29*, *64*). Once orthotopic tumors were established and confirmed via whole body bioluminescence IVIS imaging (day 7) (Fig. 6B), the mice were enrolled into treatment with gemcitabine/Abraxane or saline vehicle control twice weekly until study end point (Fig. 6A). Encouragingly, we observed an improvement in median survival for the mice bearing CAF B500 NID2 KRAB tumors in both the vehicle and chemotherapy-treated arms compared to GFP-1 KRAB mice (Fig. 6C). This survival study shows that NID2 targeting improves median survival and chemotherapy response in an orthotopic PDAC model.

**Fig. 7.**
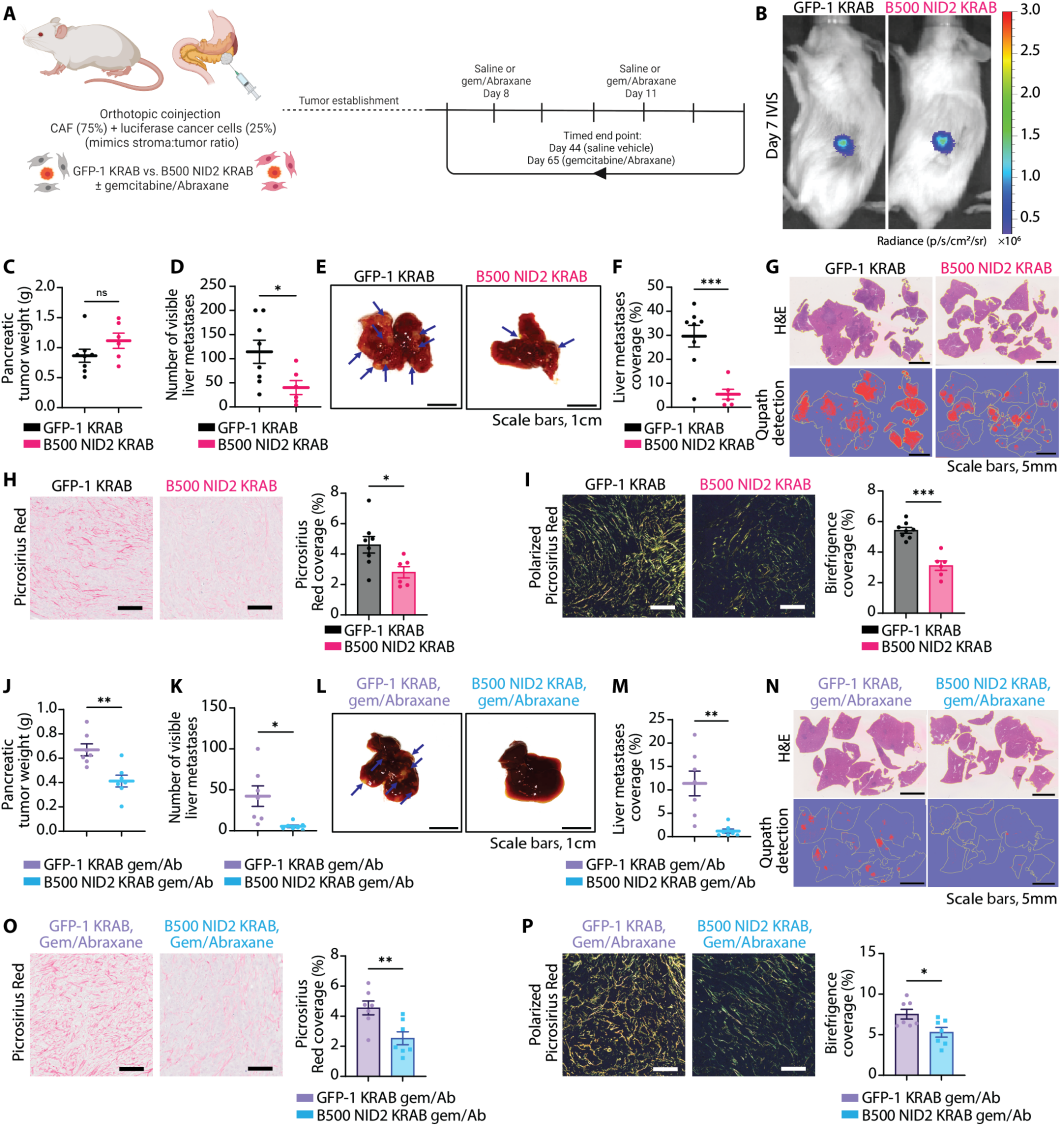
NID2 reduction in CAFs leads to reduced tumor fibrosis and liver metastasis in an orthotopic model. (**A**) Orthotopic coinjection experiment using GFP-1 KRAB or B500 NID2 KRAB CAFs (75%) with luciferase cancer cells (25%). Vehicle mice culled day 44 (*n* = 8 GFP-1 KRAB and *n* = 6 B500 NID2 KRAB). Chemotherapy mice culled day 65 (*n* = 7 GFP-1 KRAB and *n* = 7 B500 NID2 KRAB). (**B**) IVIS signal (day 7) for GFP-1 KRAB and B500 NID2 KRAB mice with Fstop1, exp30s. Color scale is radiance. (**C**) Pancreatic tumor weight (in grams). Welch’s *t* test, ns: *P* > 0.05. (**D**) Number of visible liver metastases. Welch’s *t* test, *P < 0.05. (**E**) Representative livers at end point. Scale bars, 1 cm. (**F**) Liver metastasis coverage. Welch’s *t* test, ***P < 0.001. (**G**) H&E liver sections (top) and QuPath detection (bottom). Scale bars, 5 mm. (**H**) Representative ROIs of Picrosirius Red with coverage (%) quantification. Welch’s *t* test, *P < 0.05. Scale bars, 100 μm. (**I**) Representative ROIs of polarized light Picrosirius Red with coverage (%) quantification. Welch’s *t* test, ***P < 0.001. Scale bars, 100 μm. (**J**) Pancreatic tumor weight (in grams). Welch’s *t* test, **P < 0.01. (**K**) Number of visible liver metastases. Welch’s *t* test, *P < 0.05. (**L**) Representative livers at end point. Scale bars, 1 cm. (**M**) Liver metastasis coverage. Mann-Whitney test, **P < 0.01. (**N**) H&E liver sections (top) and QuPath detection (bottom). Scale bars, 5 mm. (**O**) Representative ROIs of Picrosirius Red with coverage (%) quantification. Welch’s *t* test, **P < 0.01. Scale bars, 100 μm. (**P**) Representative ROIs of polarized light Picrosirius Red with coverage (%) quantification. Welch’s *t* test, *P < 0.05. Scale bars, 100 μm. Schematics were created with Biorender.com.

Next, we repeated this orthotopic experiment and culled the vehicle and chemotherapy arms when the first mouse within those arms reached the study end point (day 44 for vehicle arms and day 65 for gemcitabine/Abraxane arms) (Fig. 7A). As before, once orthotopic tumors were established and confirmed via bioluminescence IVIS imaging (day 7) (Fig. 7B), the mice were treated with gemcitabine/Abraxane or saline vehicle control twice weekly. For both arms, we confirmed that the B500 NID2 KRAB pancreatic tumors expressed less NID2 than the GFP-1 KRAB tumors via IHC (fig. S14, A and B), with this being independent of changes in NID1 (fig. S14, C and D). For the vehicle arm, we observed no difference in tumor weight in the B500 NID2 tumors compared to GFP-1 KRAB control (Fig. 7C); however, there was a reduction in the number of visible liver metastases (Fig. 7, D and E). Furthermore, we observed a reduction in liver metastatic burden when hematoxylin and eosin (H&E) liver sections were analyzed via QuPath (Fig. 7, F and G, and fig. S15, A and B). In addition, we also observed a reduction in Picrosirius Red staining in the B500 NID2 KRAB primary pancreatic tumors (Fig. 7H), as well as a reduction in total birefringence via polarized light imaging (Fig. 7I).

In the chemotherapy arm of the experiment, we observed a reduction in the B500 NID2 KRAB pancreatic tumor weight compared to GFP-1 KRAB tumors (Fig. 7J). In line with the vehicle arms, we also observed a reduction in the number of liver metastases (Fig. 7, K and L). Moreover, analysis of H&E liver sections further confirmed a reduction in liver metastases (Fig. 7, M and N, and fig. S15, C and D) in the mice bearing B500 NID2 KRAB tumors compared to GFP-1 KRAB control. Again, we observed a reduction in Picrosirius Red staining in the B500 NID2 KRAB primary pancreatic tumors (Fig. 7O), as well as a proportional increase in green collagen fibers and decrease in yellow collagen fibers via polarized light imaging (Fig. 7P). We also observed an increase in CD31 coverage in the B500 NID2 KRAB primary tumors compared to GFP-1 KRAB control (fig. S15E), mirroring the subcutaneous study results. Overall, these orthotopic experiments demonstrate the role NID2 plays in facilitating pancreatic cancer metastasis.

Collectively, using tissue decellularization coupled with temporally resolved MS proteomics, we reveal NID2 as a promising cotarget with a potential dual role in pancreatic cancer. That is, (i) an antimetastatic effect alone and, (ii) in the context of chemotherapy, NID2 effects on tumor vasculature likely also improve response to chemotherapy in this deadly disease.

## DISCUSSION

Fibrosis is an important driver of pancreatic tumor progression, invasion, and metastasis as well as therapy resistance (*4*–*7*). Consequently, stromal cotargeting to improve therapeutic efficacy has become an active area of pancreatic cancer research. To provide a better understanding of the complex and time-dependent characteristics of pancreatic cancer fibrosis, this study has used matrisome-enriched MS proteomic profiling to assess the matrisomal proteins of GEMMs that closely recapitulate human PDAC disease (*36*, *37*), relative to normal age-matched WT pancreas. This approach allowed us to dissect the fibrotic signatures of pancreatic tumors to find matrisomal cotargets such as NID2 in PDAC.

Tumor matrix mechanics, deposition, and remodeling are dynamic processes, which we and others have shown can be linked to cancer cell genotype and mutational status (*16*, *28*, *46*). Here, we analyzed tumors from the highly metastatic KPC model and compared them to the poorly metastatic KP^fl^C model at three different time points to capture the temporally regulated tumor matrisome specifically associated with metastatic disease (*36*, *37*). Understanding how individual matrisomal proteins are regulated at clinically relevant time points such as PanIN development (early stage), widespread PanIN formation (mid-stage), and fully developed PDAC with metastases (late stage) is critical to elucidating which proteins are the key regulators of tumor invasion and metastasis. By dissecting protein expression according to disease stage, we were able to identify NID2, which was increased in mid-stage KPC tumors compared with KP^fl^C tumors. Through organotypic models, advanced intravital imaging, and coinjection in vivo experiments using NID2-depleted CAFs, we have shown that NID2 contributes to tumor fibrosis and metastasis, while in the context of gemcitabine/Abraxane chemotherapy, NID2 can alter tumor vasculature and improve response the therapy.

Matrix proteins have a relatively long half-life, which renders proteomics a useful approach to accurately characterizing the matrisome of low cellularity tumors such as PDAC. Thus far, several studies have aimed to characterize the fibrotic matrisome of pancreatic cancer such as Barrett *et al.* (*48*) and Tian *et al.* (*49*). Encouragingly, our dataset was largely congruent with these previously published pancreatic cancer matrix datasets, despite differences in GEMMs and matrix enrichment methodologies (*48*, *49*). We also identified matrix proteins that were not represented in the datasets of Barrett *et al.* (*48*) and Tian *et al.* (*49*), highlighting the added value of our tissue decellularization approach and the additional comparison of KP^fl^C versus KPC GEMMs in this study. In addition to the two cancer models, we also analyzed the normal murine pancreas at the three time points (early-, mid-, and late-stage disease) to generate age-matched controls. Future work could include interrogating the datasets for KPC and KP^fl^C versus WT pancreas at different stages of tumor development to assess matrix proteins commonly up-regulated in both genotypes compared to control to potentially reveal stage-dependent targets. Hence, we envision that our dataset will serve as a valuable resource for others studying pancreatic cancer fibrosis to further assess matrix targets.

In this study, we focused on the functional role of NID2, which was more abundant in the highly metastatic KPC model compared to the poorly metastatic KP^fl^C model. Hence, we investigated how NID2 can promote pancreatic cancer invasion and metastasis. The nidogens comprise a family of related glycoproteins, consisting of NID1 and NID2 (*39*). Both are BM proteins, with NID1 more abundantly expressed, whereas NID2 displays distinct expression patterns throughout development and in adulthood (*39*). In particular, NID2 is predominantly associated with endothelial BMs, whereas NID1 is more ubiquitously expressed (*74*). Although there is some evidence of NID1/NID2 redundancy mechanisms in BM formation and integrity (*74*, *75*), we show both in vitro and in vivo that NID1 levels remained unchanged during NID2 targeting. In addition, it is not well understood which signaling pathways regulate increased NID2 expression in the context of pancreatic cancer. It is possible that transforming growth factor–β (TGF-β) signaling may, in part, be responsible for this, as others have shown that NID2 expression is increased with TGF-β stimulation in other stromal cells such as vascular smooth muscle cells (*76*). TGF-β is also critical for regulating CAF heterogeneity in the PDAC TME and metastatic spread (*9*, *77*). Future work could include investigating the reciprocal signaling between pancreatic cancer cells and fibroblasts, which occurs to drive increased stromal NID2 expression.

Before this work, NID2 has been identified as a BM protein associated with poor outcomes in solid tumors. For example, a breast and ovarian cancer BM study by Reuten *et al.* (*45*) identified that NID2 was the third most strongly associated BM protein with poor survival in breast cancer, behind well-known cancer-associated matrix proteins laminin α5 (LAMA5) and fibronectin. Furthermore, in a scRNA-seq study of mammary specific polyomavirus middle T antigen overexpression mouse model (MMTV-PyMT) breast tumors by Bartoschek and colleagues (*44*), NID2 was identified as a marker of vascular CAFs, which may be linked to the expression of NID2 at vascular BMs (*74*). Although vascular CAFs have not been explicitly identified as a separate CAF subpopulation in pancreatic cancer, Garcia *et al.* (*13*) recently identified that perivascular Gli1^+^-resident fibroblasts are one of the main origins of CAFs in murine pancreatic tumors using lineage-tracing models. In this study, we stained KPC tumor tissues for NID2, CD31 (endothelial cells), and CD146 (perivascular stromal cells) (*60*). We observed high correlations between NID2 and the perivascular niche, similar to that observed by Bartoschek *et al.* (*44*). These data across different studies warrant future investigation into the perivascular stromal cells of pancreatic tumors and their possible role in providing NID2-rich CAFs, which then could consequently orchestrate further tumor development and progression.

Thus far, several studies have highlighted the utility of NID2 as a molecular or serum biomarker for several malignancies including gastric cancer (*40*), esophageal squamous cell carcinoma (*41*), ovarian cancer (*42*), melanoma (*43*), and breast cancer (*44*). In pancreatic cancer, we now show that high NID2 expression is associated with poor patient survival, particularly for basal-like tumors, via analysis of the ICGC PDAC cohort. By interrogating publicly available scRNA-seq data from specimens of both the KPC mouse model (*8*) and patient with PDAC (*59*), we confirm that *NID2* is largely expressed by stromal cells, including fibroblasts and stellate cells, which are the main contributors to matrix deposition and remodeling in pancreatic cancer (*4*, *6*, *49*, *67*). Using CRISPRi technology, we knocked down the expression of NID2 in CAFs to assess its functional role in pancreatic cancer. We found that reducing NID2 expression in CAFs reduced SHG signal in 3D organotypic matrices, which is a surrogate readout of fibrillar collagens. Furthermore, NID2 knockdown organotypic matrices had lower Picrosirius Red signal, while birefringence analysis of these samples showed that collagen birefringence proportions were changed, with a higher proportion of green fibers in NID2-depleted settings compared to control. Notably, the NID2-depleted organotypic matrices were softer than their control counterparts, as shown by biomechanical compression analysis. Collectively, we show that depleting NID2 results in biochemical and biomechanical alterations, which also impairs subsequent cancer cell invasion in 3D organotypic models. This aligns with previous studies, where alterations in tissue stiffness and biomechanics have been shown to affect cellular processes such as cancer cell migration and invasion (*15*, *29*, *65*–*67*).

Intravital imaging is a powerful technology that can be used to quantify changes in live tissues in an intact environment (*15*, *69*–*72*). Here, we used intravital imaging to assess tumor characteristics in live subcutaneous tumors consisting of either control CAFs or NID2-depleted CAFs coinjected with eGFP-tagged cancer cells. Using quantum dots in conjunction with VesselVio analysis (*73*), we were able to capture several biologically relevant parameters including increased vessel volume and surface area, as well as increased mean vessel radius. These live data support our CD31 IHC analysis, which shows an increase in CD31 coverage in NID2-depleted tumors collected at end point. Together, we show that NID2 targeting alters the vasculature patency of tumors in these models (*15*, *19*, *20*). We posit that this could be why tumor growth was impeded, as there may have been improved delivery of gemcitabine/Abraxane chemotherapy to the tumor site, as previously observed in stromal targeting studies (*15*, *19*, *20*).

In the clinic, patients with PDAC with resectable or borderline-resectable PDAC (~20%) are increasingly receiving neoadjuvant treatments such as chemotherapy or chemoradiotherapy to debulk their tumor mass before resection and subsequent adjuvant chemotherapy (*2*). Recent clinical trials have shown that this can be beneficial to patient outcomes, particularly those who are borderline resectable (*2*). Radiation and chemotherapy agents can cause additional fibrosis in the pancreatic TME (*29*, *32*, *78*), in part due to the reactive response of the stroma including CAFs. Recent studies have shown that therapy-induced fibrosis can accelerate tumor progression and alter the immune signatures of tumors (*78*). Now that we have established our approach to identifying matrisomal changes over disease progression using tissue decellularization and MS proteomics, this technology could be repurposed to assess the matrix signatures of radiation or chemotherapy-treated animals bearing pancreatic tumors to further assess therapy-induced fibrosis.

Unfortunately, most patients with PDAC (~80%) present with metastatic disease, making surgical intervention unfeasible. For these patients, systemic chemotherapy is standard of care (*2*). In our in vivo work, we show that depleting NID2 in the TME enhances the efficacy of standard-of-care gemcitabine/Abraxane chemotherapy, reducing both primary tumor growth and liver metastasis. Furthermore, we show that NID2-depleted tumors have reduced fibrosis compared to control. Using intravital imaging, we show that NID2 targeting increases vascular coverage and promotes the formation of more patent vessel networks in live pancreatic tumors under chemotherapy treatment. Metastasis is a major cause of mortality in patients with pancreatic cancer, with a 5-year survival rate of ~3% in “distant” metastatic cases (*1*). Our orthotopic coinjection experiments with control or NID2 knockdown CAFs with cancer cells reveal that NID2 targeting can reduce primary tumor growth (with chemotherapy treatment) and reduce metastatic burden in the liver, leading to improvements in median survival. NID2 has been previously shown to play a role in the outgrowth of neurons (*79*). Considering the known aberrations in axonal guidance genes within metastatic pancreatic cancer (*80*), future work could involve an assessment in the NID2-depleted setting for these pathways. On the basis of our promising data, future work could also involve developing an anti-NID2 therapy to promote normalization of the stroma and decreased tumor stiffness. A putative anti-NID2 approach could then be combined with standard-of-care chemotherapy regimens, allowing for increased chemotherapy response. Comparable strategies have been used with other matrix-associated targets with promising results such as with the LOX family (*32*, *52*), focal adhesion kinase (*29*), rho-associated protein kinase (*15*), integrin subunit alpha 5 (*31*), and prolyl isomerase (*81*), among others (*4*, *7*).

Our exploration of NID2 biology in pancreatic cancer was conducted using standard-of-care gemcitabine/Abraxane chemotherapy with immunocompromised mouse models. However, future work could include investigating other chemotherapy combinations such as gemcitabine/capecitabine, FOLFIRINOX (irinotecan, fluorouracil, leucovorin, and oxaliplatin), or its variations (*2*) to assess whether NID2 targeting could also improve response with these therapies. Similarly, immunotherapy resistance has been linked to a fibrotic TME in many solid tumor types, including pancreatic cancer (*11*, *23*, *30*). High NID2 has also been associated with promoting an immunosuppressive microenvironment in melanoma, leading to poor response to checkpoint inhibitors (*43*). In the future, it would be interesting to investigate the role of anti-NID2 in the context of syngeneic mouse models of PDAC via immunotherapy or an antitumor vaccine approach, as others have done with matrix targets such as prolyl isomerase (*81*), microfibril-associated protein 5 (MFAP5) (*82*), CXCL12/fibroblast activation protein (*83*), and focal adhesion kinase (*22*, *84*). Our data show that the future development of an NID2-targeting approach offers an attractive option to not only reduce primary tumor growth but also impede liver metastasis in this highly aggressive and metastatic disease.

In summary, our data show that matrisomal characterization of pancreatic tumors using a tissue decellularization and temporal MS proteomics approach is a powerful means to find stromal cotargets at key stages of tumor progression, invasion, and metastasis. Our data reinforce the emerging body of work currently underway to develop new stroma modulating strategies in pancreatic cancer to ultimately improve patient outcomes in this deadly disease.

## MATERIALS AND METHODS

### Study design

This study assesses the matrix signatures of the KPC, KP^fl^C, and WT mouse pancreatic tissue using an adapted tissue decellularization (ISDoT) protocol and MS proteomics. DIA LC-MS/MS was performed on five independent biological replicates (apart from the mid-stage KPC tumor condition, which had four biological replicates) in the same MS run. This study also investigates the modulation of the pancreatic TME via NID2 reduction in combination with gemcitabine/Abraxane chemotherapy in PDAC. *NID2* expression was assessed via the analysis of mRNA expression from the TCGA [OncoDB (*57*)], ICGC, and also two publicly available scRNA-seq datasets (*8*, *59*). Transcriptional and protein changes were assessed in NID2-depleted and control CAF cell lines by RNA-seq and DIA LC-MS/MS, with three biological repeats per cell line performed. For Western blotting, three biological repeats were performed per condition. For RT-qPCR, CDMs, and 3D organotypic matrix studies, at least three biological repeats were performed with at least three technical replicates per repeat per condition. In vitro IHC, SHG, IF, Picrosirius Red, and birefringence analysis were performed on at least three representative regions of interest (ROIs) per technical replicate per experimental repeat per condition. Mouse numbers used in in vivo experiments are outlined in the corresponding figure legends. In vivo IHC, SHG, Picrosirius Red, and birefringence analysis were performed on eight representative ROIs for subcutaneous and orthotopic tumors per condition. For intravital studies, imaging was performed on at least four representative ROIs per mouse. For tissue IF, imaging was performed on at least eight representative ROIs per section.

### Statistical analysis

For proteomic analysis, Perseus (v1.6.7.0) was used for two-sample *t* tests, with an FDR of 0.05 and a S_0_ value of 0.1. For RNA-seq, *P* values were adjusted using the Benjamini-Hochberg procedure, with adjusted *P* < 0.05 and a log_2_ fold change of >1.5 considered significant. For mRNA expression analysis of the ICGC cohort, statistical tests were performed using R (v4.0.2), survival (v3.1-12), survminer (v0.4.8), and ggplot2 (v3.3.2). All other statistical analysis was performed using GraphPad Prism v9 (GraphPad Software Inc., CA). All data were subjected to a Shapiro-Wilk normality test, unless otherwise stated. For parametric two sample data, an unpaired Welch’s *t* test was used, unless otherwise stated. For parametric data with greater than two samples, a one-way analysis of variance (ANOVA) with Dunnett’s test was used, unless otherwise stated. For nonparametric two sample data, a Mann-Whitney test was used, unless otherwise stated. For nonparametric data with greater than two samples, a Kruskal-Wallis test with Dunn’s test was used, unless otherwise stated. For RT-qPCR analysis with two samples, a parametric two-way ANOVA with Sidak’s test was used, and for experiments with more than two samples, a parametric two-way ANOVA with Dunnett’s test was used, unless otherwise stated. For data normalized to 1, a one-sample *t* test was performed. Kaplan-Meier curves were compared with a log-rank Mantel-Cox test. For scRNA-seq from Peng *et al.* (*59*), a two-sided *t* test was used. Statistical significance was described as nonsignificant (ns) *P* > 0.05, *P < 0.05, **P < 0.01, and ***P < 0.001.

### Animals

All animal work was performed in compliance with the Australian Code of Practice for the Care and Use of Animals for Scientific Purposes (National Health and Medical Research Council). All protocols and study end points were approved by the St. Vincent’s Precinct and Garvan Institute Animal Ethics Committee (ARA 16/13, 19/06, 19/08, 19/10, 19/13, 22/04, 22/08, 22/09, and 22/10). The number of mice used in each experiment is detailed in corresponding figure legends. KPC, KP^fl^C, and WT mouse pancreatic tissue were harvested at early-stage (41 to 53 days), mid-stage (70 to 84 days), and late-stage (99 to 212 days) time points (table S1). In the tumor setting, early-stage disease corresponds to the development of PanINs, mid-stage disease corresponds to later-stage widespread PanINs in the KPC model, and late-stage corresponds to PDAC with liver metastases in the KPC model (*28*, *36*, *37*).

### ISDoT tissue decellularization

To obtain decellularized pancreatic tissues for subsequent IF, ISDoT was performed as previously described (*34*, *35*). Briefly, the mice were euthanized via CO_2_ inhalation. Fur was removed via shaving, and the skin was disinfected with 70% ethanol. The mouse was pinned to a fixed surface, and an incision was made at the midline from the lower abdomen to the submandibular region. From here, the thorax was accessed by bilateral sectioning of the pectoralis and intercostal muscles through the sixth intercostal space. This was followed by perpendicularly sectioning the sternum along the midline axis. The thoracic walls were then elevated and secured with pins. An incision was then made 1.5 cm above the diaphragm. Using a 26-gauge catheter, the aorta was catheterized, with the needle inserted until the tip was just above the diaphragm. The lower peritoneal wall was then opened using a middle incision and discharge incisions. From here, the intestines, spleen, pancreas, stomach, and liver to the right (of the mouse) were elevated to reveal the left kidney and its vessels. From here, a section was made in the diaphragm above the liver to reveal the aorta, the emergence of the coeliac trunk, and the superior mesenteric artery running to the right. The 26-gauge catheter was then pushed to the emergence of the coeliac trunk. The catheter was then fixed in place with 2-3 sutures (6-0 vicryl). The superior mesenteric artery was ligated. After this, the permeability of the coeliac trunk and superior mesenteric artery was tested by delicately injecting 1 ml of phosphate-buffered saline (PBS; Gibco) via the catheter. From here, the catheter was connected to a peristaltic pump with a flow output of between 200 and 400 μl/min. The tissues were then perfused with Milli-Q H_2_O for 2 hours, followed by 0.5% sodium deoxycholate (SDC) overnight and then Milli-Q H_2_O again for 3 hours. From here, the decellularized pancreatic tissues were dissected for antibody immunostaining or dark-field microscopy.

For immunostaining, pancreatic tissues were twice washed with Milli-Q H_2_O for 30 min. The tissues were blocked with 6% goat serum in PBS for 1 hour at room temperature. From here, the tissues were twice washed with 0.05% Tween 20 in Milli-Q H_2_O for 1 hour. The tissues were then incubated in anti–collagen IV (1:100 in PBS; rabbit polyclonal antibody, ab6586, Abcam) for 2 hours. After this, tissues were then washed three times in 0.02% Tween 20 in Milli-Q H_2_O for 30 min. Then, tissues were incubated in Alexa Fluor 488–conjugated secondary antibody against rabbit (1:500 in PBS) for 2 hours. Stained tissues were then washed in 0.02% Tween 20 in Milli-Q H_2_O. All immunostaining and washing steps were performed on a shaker at 10 rpm. The stained decellularized tissue was then imaged using a Leica SP8 microscope (Garvan ACRF INCITe Centre) equipped with 40× objective oil immersion lens (HC PL APO CS2 40×/numerical aperture, 1.30 oil). Alexa Fluor 488 fluorescence was excited with a 488-nm diode laser. Emission was detected on an internal photomultiplier tube with a variable band-pass filter tuned to 492 to 514 nm. Representative ROIs of 1024 pixels by 1024 pixels (Zoom 0.75) were imaged over a 3D z-stack (1-μm step size).

For dark-field microscopy, samples were suspended in PBS and before imaging transferred to a 35-mm glass-bottom dish (Corning). During imaging, PBS was added to the sample to prevent drying out. Dark-field imaging was performed using a Leica DM IL light-emitting diode microscope equipped with a dark-field condenser and a 10× objective. A white light-emitting diode light source was used to provide oblique illumination. The camera settings, including exposure time and gain, were optimized to ensure high-quality images but kept consistent between conditions.

### MS mouse cohort

KPC, KP^fl^C, and WT mice were aged to early-stage (41 to 53 days), mid-stage (70 to 84 days), and late-stage (99 to 212 days) diseases with details found in table S1. Mice were euthanized via cervical dislocation, and the pancreatic tissue was excised. Pieces of the pancreatic tissue were then collected for DIA LC-MS/MS proteomics or fixed in 10% buffered formalin or optimal cutting temperature (OCT) compound-embedded (Scigen).

### MS proteomics sample preparation

Pancreatic tissues from KPC, KP^fl^C, and WT mice (*n* = 44) were incubated for 16 hours at room temperature on a shaker in 0.5% SDC to enrich for matrix proteins (*34*, *35*). The samples were then centrifuged at 1000*g* for 2 min, with the supernatant discarded. The remaining pellets were then washed briefly with 0.5% SDC and centrifuged at 1000*g* for 2 min, with the supernatant discarded. The decellularized proteins were then resuspended in 1% SDS in 100 mM tris (pH 8.5) and solubilized with 2× 20-s tip-probe sonication. Protein was quantified using a BCA Protein Assay kit and normalized to 20 μg/100 μl in 1% SDS in 100 mM tris (pH 8.5). Samples were then reduced and alkylated with a final concentration of 10 mM tris(2-carboxyethyl)phosphine and 40 mM 2-chloroacetamide for 5 min at 45°C. From here, peptides were prepared using a modified single-pot, solid phase–enhanced sample preparation protocol (*85*). First, the samples were diluted in 50% ethanol and incubated with hydrophilic:hydrophobic (1:1 mixture) Sera-Mag SpeedBead carboxyl magnetic beads for 8 min at room temperature. After this, the supernatant was removed, with the magnetic beads washed three times with 80% ethanol. The beads were then resuspended in 10% trifluoroethanol in 100 mM tris-HCl (pH 7.5). The beads were then added to 0.4 μg of sequencing-grade trypsin and 0.4 μg of sequencing-grade LysC on a shaker at 37°C overnight. To halt the trypsin digestion, 1% trifluoroacetic acid (TFA) was added. The samples were then purified using styrene divinylbenzene–reverse phase sulfonate microcolumns. The columns were rinsed with 99% isopropanol containing 1% TFA, followed by 5% acetonitrile containing 0.2% TFA. The peptides were then eluted using 80% acetonitrile containing 1% ammonium hydroxide. The eluted peptides were dried using vacuum centrifugation and resuspended in 2% acetonitrile containing 0.1% TFA. Peptides were stored at −20°C before running.

### MS proteomics data analysis

Peptide samples were analyzed using a Dionex 3500RS nano–ultrahigh-performance LC coupled to an Orbitrap Fusion mass spectrometer in positive mode. A 20-cm by 100-μm column with an integrated emitter and packed with 1.9-μm C18AQ particles (built in-house by Dr. Maisch, Germany) was used to separate the peptides. One microgram of peptide was injected and eluted by a linear gradient (3 to 25%) buffer system of buffer B (80% acetonitrile plus 0.1% formic acid) and buffer A (0.1% formic acid) over 1 hour with a flow rate of 800 nl/min at 60°C. The mass spectrometer was operated in DIA mode with the same settings and variable-sized isolation windows as previously reported (*86*). Library-free searching was used to analyze the DIA data in SpectronautPulsar X. Peptide quantification was carried out at MS2 level using three to six fragment ions, with automatic interference fragment ion removal as previously described (*86*). The MS1 mass tolerance was set to 20 parts per million (ppm), while the mass tolerance for MS/MS fragments was set to 0.02 Da. Dynamic mass MS1 and MS2 mass tolerance was enabled, and retention time calibration was accomplished using local (nonlinear) regression. A dynamic extracted ion chromatogram window size was performed. The minimum peptide length was set to seven amino acids with specific trypsin cleavage, and search criteria included oxidation of methionine and protein N-terminal acetylation set as variable modifications and carbamidomethylation set as a fixed modification. Data were searched and filtered against the *M. musculus* (mouse) UniProt database (2018; UP000000589) and filtered to an FDR of 0.01 at the peptide and protein level (*Q* value cutoff < 0.01). Peptide quantification was performed using three to six fragment ions, and protein quantification was performed with weighted peptide median values. Perseus (MaxQuant) software was used for statistical analysis. Raw data were normalized using log_2_(*x*) transformation and median subtraction. The data were then annotated for matrisome proteins based on the Naba *et al.* (*33*) classification, with nonmatrisome proteins filtered out. A filter was then applied to only include proteins that were detected at least five times across the whole dataset. Each sample was then assigned a condition based on the genotype and age of the mouse (WT-early, WT-mid, WT-late, KP^fl^C-early, KP^fl^C-mid, KP^fl^C-late, KPC-early, KPC-mid, and KPC-late). From here, missing values were imputed from the normal distribution for conditions missing less than two biological replicates. To assess for differentially abundant proteins between conditions, two sample *t* test volcano plot analyses were performed using an FDR of 0.05 and a S0 value of 0.1. Principal components analysis was generated using the average log_2_(*x*) transformation and median subtracted value of each condition.

### scRNA-seq analysis

The expression of *NID2* in scRNA-seq datasets from human (*59*) and mouse PDAC tumors (*8*), generated on the Chromium platform (10× Genomics), were assessed. Raw or normalized count matrices were pulled from each respective study and processed using Seurat v4.1.1 using default parameters recommended by the developers. Mouse PDAC scRNA-seq data (*8*) were annotated using the SingleR package using the mouse ImmGen reference. Statistical significance of *NID2* expression in stromal cells from normal human pancreas and PDAC groups in Peng *et al.* (*59*) was determined with a two-sided *t* test using log-normalized expression values.

### NID2 expression in OncoDB and ICGC

The OncoDB database (www.oncodb.org) was assessed for *NID2* expression in patients with PDAC (*57*). RNA-seq data of pancreatic tissue obtained from patients with PDAC (*n* = 178) and controls (*n* = 200) were analyzed for *NID2* transcripts per million and subjected to nonparametric Mann-Whitney test. Patient survival and the association with *NID2* expression were assessed via the analysis of mRNA expression from the ICGC PACA-AU (Pancreatic Cancer - Australia) microarray dataset (*n* = 267). Patients were segregated into low (bottom, 75%; *n* = 200) and high (top, 25%; *n* = 67) NID2-expressing groups, and Kaplan-Meier curves were generated with the log-rank test. In addition, patients were further categorized according to molecular subtype, namely, 204 PDAC tumors were identified as basal-like (*n* = 89) or classical (*n* = 115), according to the molecular classification previously described by Moffitt *et al.* (*58*), and further studied by de Santiago *et al.* (*87*). Kaplan-Meier curves were generated for each subtype using the median of NID2 expression to define “high” and “low” groups, with *P* values indicating statistical significance, and whether mRNA expression was associated with length of survival. Statistical tests were performed using R (v4.0.2), survival (v3.1-12), survminer (v0.4.8), and ggplot2 (v3.3.2).

### Cell culture

KPC cancer cells (TB32043) (*36*) and KPC CAFs (mt-CAF) (*28*) were cultured in Dulbecco’s modified Eagle’s medium (DMEM; high glucose, high pyruvate; Gibco) supplemented with 10% fetal bovine serum (FBS; HyClone) and 1% penicillin/streptomycin (Thermo Fisher Scientific; referred to as complete DMEM henceforth) at 37°C with 20% O_2_ and 5% CO_2_. KPC cancer cells were engineered to express eGFP or luciferase using a third-generation lentiviral packaging system, followed by selection via fluorescence-activated cell sorting on an FACSAria III Cell Sorter (BD Biosciences, USA), as previously described (*15*, *28*, *29*). All cell lines were routinely tested for mycoplasma (all negative results).

### Generation of stable CRISPRi cell lines

For CRISPRi, SpyCas9 was rendered catalytically inactive by introducing D10A and H840A mutations and cloned in frame with a KRAB domain at its N terminus into the third-generation lentiviral vector as previously described (*28*). Guide RNAs were designed to bind the following genomic DNA protospacer adjacent motifs (PAM) sequences: “B500 NID2 KRAB,” 5′-AGCAGCGATAGCGGTGATGG(CGG)-3′; “C500 NID2 KRAB,” 5′-TGCGGACCAGAGGTCCAAGT(TGG)-3′, located in the exon 1 and 5′ untranslated region of mouse NID2 gene. As control “GFP-1 KRAB”, a guide RNA targeting the eGFP-1 sequence [5′-GACCAGGATGGGCACCACCC(CGG)-3′] was used. Lentiviral particles were harvested from transfected human embryonic kidney 293T culture medium, filtered (0.45 μm), and used to transduce KPC CAFs. After 48 hours, CAFs were selected for puromycin resistance (40 μg/ml) in the presence of polybrene (8 μg/ml; Millipore). After puromycin selection, NID2 expression was assessed by RT-qPCR (exon 1/2 and exon 6/7) and Western blotting to confirm repressed expression.

### RNA-seq of CAF cell lines

RNA was extracted from GFP-1 KRAB, B500 NID2 KRAB, and C500 NID2 KRAB CAFs using the QIAGEN RNeasy Mini Kit (catalog no. 74104) according to the manufacturer’s instructions for animal cells (*n* = 3 biological replicates per cell line). RNA concentration and integrity were assessed using the Agilent 4200 TapeStation system, which confirmed sample RNA integrity numbers of >9. Library preparation was performed using the TruSeq Stranded mRNA Kit according to the manufacturer’s protocol (Roche), and paired-end sequencing was performed using the Illumina NovaSeq 6000. Sequence reads were aligned using STAR (version 2.7.3a) to the *M. musculus* (mm10) assembly with the gene, transcript, and exon features of Genecode release M10 gene model. Expression counts were estimated using RSEM (RNA-Seq by Expectation-Maximization; version 1.2.26). Library sizes were normalized by calculating the counts per million mapped reads for each sample. For differential expression analysis, effective library sizes were estimated using the trimmed mean of *M* values method via the edgeR package, followed by the application of the voom function from the limma-voom R package. Differential expression analysis was conducted by comparing various cell lines against the GFP-1 KRAB control using a design matrix specified as “~ 0 + groups.” *P* values were adjusted using the Benjamini-Hochberg procedure to control the FDR.

### CAF cell line MS proteomics sample preparation

GFP-1 KRAB, B500 NID2 KRAB, and C500 NID2 KRAB CAF cell lines were cultured in complete DMEM at 37°C with 20% O_2_ and 5% CO_2_ in 10-cm petri dishes (Corning) for 48 hours. Three biological repeats for each CAF line were obtained from different passages (*n* = 9 samples total). To collect samples, cells were washed twice with sterile cold PBS and then scrapped from the dish into Eppendorfs. Samples were then centrifuged at 10,000 rpm at 4°C, after which the supernatant was removed. The resulting cell pellets were then snap-frozen on dry ice and stored at −80°C until MS sample preparation.

Samples were lysed with a bead beater in 100 μl of 4% SDC and 50 mM ammonium bicarbonate for 2 min at 50 Hz and stored overnight at −30°C in trichloroacetic acid (TCA)/acetone (400 μl). The samples were centrifuged at 9000*g* for 20 min, and the supernatant was removed. The cell pellet was denatured in urea/thiourea (100 μl), reduced in 100 mM dithiothreitol (10 μl) for 1 hour at room temperature, and alkylated in 250 mM 2-iodoacetamide (10 μl) in the dark for 30 min. Protein concentration was determined using qubit (Protein Assay Kit, Life Technologies). Protein (5 μg) was removed and digested with trypsin (1 μg/μl; Promega) overnight. Peptides were concentrated and desalted using C18 Zip-Tips (Millipore, Bedford, MA) as per the manufacturer’s instructions. Peptides were resuspended in 10 μl of 3% (v/v) of acetonitrile/0.1% (v/v) of formic acid and briefly sonicated.

### CAF cell line MS proteomics data analysis

Samples were separated by nano-LC using an Ultimate 3000 nano-LC and autosampler system (Thermo Fisher Scientific, Scoresby) coupled to an in-house fritless nano 75-μm by 40-cm column packed with ReproSil Pur 120 C18 stationary phase (1.9 μm; Dr. Maisch GmbH, Germany). LC mobile phase buffers were composed of A—0.1% (v/v) of formic acid—and B—80% (v/v) of acetonitrile/0.1% (v/v) of formic acid. Peptides were eluted using a linear gradient of 5% B to 30% B over 110 min and then 95% B wash over 7 min at a flow rate of 300 nl/min. The LC was coupled to an HFX Q-Exactive Orbitrap mass spectrometer (Thermo Fisher Scientific, Scoresby). Column voltage was 2300 V, and the heated capillary was set to 275°C. Positive ions were generated by electrospray and the Orbitrap operated in data-dependent acquisition mode. A survey scan of 350 to 1650 mass/charge ratio (*m*/*z*) was acquired (resolution = 60,000, with an accumulation target value of 3,000,000 ions), and target value of 100,000 ions was collected. Ions selected for MS/MS were dynamically excluded for 15 s. The data were analyzed using Proteome Discoverer vr 2.5 (Thermo Fisher Scientific) and Mascot vr 2.8 (Matrix Science, London). The search parameters included the following variable modifications: oxidized methionine and carbamidomethyl cysteine. The enzyme was set to trypsin, and precursor mass tolerance was 10 ppm, while the fragment tolerance was 0.05 Da. Data were searched and filtered against the *M. musculus* (mouse) UniProt database (2023; UP000000589) and filtered to an FDR of 0.01 at the peptide and protein level (*Q* value cutoff < 0.01). Peptide quantification was performed using three to six fragment ions, and protein quantification was performed with weighted peptide median values. Perseus (MaxQuant) software was used for statistical analysis. Raw data were normalized using log_2_(*x*) transformation and median subtraction. A filter was then applied to only include proteins that were detected at least once across the whole dataset. Each sample was then assigned a condition (CAF GFP-1 KRAB was assigned “control,” and CAF B500 NID2 KRAB and CAF C500 NID2 KRAB were assigned “NID2 knockdown”). To assess for differentially abundant proteins between conditions, two-sample *t* test volcano plot analyses were performed using an FDR of 0.05 and a S_0_ value of 0.1.

### Cell-derived matrices

CDMs were generated as previously described (*61*, *62*). Briefly, for SHG imaging, KPC CAFs were seeded in a glass-bottom 24-well plate (Corning) at 100,000 cells per well (day 0) in complete DMEM at 37°C with 20% O_2_ and 5% CO_2_. For the Sircol soluble collagen concentration assay, 12-well cell culture plates (Costar, Corning) were first coated with 2% gelatin (G1393, Sigma-Aldrich) in PBS and incubated for 1 hour at 37°C. After this, the wells were washed twice with PBS and then fixed with 10% buffered formalin for 30 min at room temperature. After washing the wells twice with PBS, the wells were quenched with 1 M sterile glycine (Sigma-Aldrich) in PBS for 20 min at room temperature. Wells were then washed twice with PBS. Wells were then incubated with a 1:1 solution of FBS and PBS for 5 min at room temperature. Wells were then incubated in complete DMEM for 30 min at 37°C. After washing wells with PBS twice, KPC CAFs were seeded in the gelatin-coated 12-well cell culture plates at 200,000 cells per well (day 0). For both SHG and Sircol CDMs, cells were treated with ascorbic acid (50 μg/ml) on days 2, 4, and 6 to stimulate matrix production. On day 7, CDMs were imaged using SHG imaging or analyzed for collagen concentration via the Sircol soluble collagen concentration assay.

### Sircol soluble collagen concentration assay

On day 7, complete DMEM was aspirated, and cells were treated with cold acetic acid (0.5 M) and pepsin (0.1 mg/ml) overnight at 4°C to induce an acid-pepsin extraction of collagen (Sircol soluble collagen assay kit, Biocolor Life Science Assays). On day 8, all supernatants were harvested, and an additional collagen isolation and concentration step was performed before the addition of colorimetric dye reagent, as per the manufacturer’s instructions (Sircol soluble collagen assay kit, Biocolor Life Science Assays). Collagen concentration was determined by absorbance at 556 nm and plotted on a standard curve of concentrations, according to kit standard concentrations.

### Collagen I extraction

Collagen I was extracted from rat tails as previously described (*15*, *28*, *29*, *64*). Briefly, tendons were removed from dissected tails using forceps. From here, tendons were solubilized in 0.5 M acetic acid at 4°C for 48 hours while stirring. After this, this mixture was filtered to remove any tail tissue and insolubilized tendons. The filtered mixture was then precipitated with 10% (w/v) of sodium chloride at 4°C for 6 to 8 hours with stirring. This mixture was then centrifuged for 30 min at 4°C (10,000 rpm). The supernatant was then discarded, with the remaining precipitate added to 0.25 M acetic acid overnight at 4°C with stirring. After this, the mixture was then dialyzed in 17.4 mM acetic acid over 72 hours. The remaining collagen was then diluted with 17.4 mM acetic acid and stored at 4°C.

### Organotypic contraction assay

3D organotypic matrices were generated as previously described (*15*, *28*, *29*, *63*, *64*). Briefly, 200,000 KPC CAFs (GFP-1 KRAB, B500 NID2 KRAB, or C500 NID2 KRAB) were embedded in 2.5 ml of rat tail collagen (~2.5 mg/ml) in a 6-well culture dish (Corning). Matrices were allowed to polymerize at 37°C before being detached from the well using a sterile glass pipette. 3D matrices were then cultured in complete DMEM with medium changes performed at days 3, 6, and 9. On day 12, contracted organotypic matrices were then either subjected to biomechanical testing, imaged using SHG imaging, fixed in 10% buffered formalin, and paraffin-embedded for downstream IHC analyses or used in cancer cell invasion assays. Matrices were imaged using a flatbed scanner at days 3, 6, 9, and 12, with matrix area measured using ImageJ [FIJI, National Institutes of Health (NIH)].

### Organotypic cancer cell invasion assay

Organotypic invasion assays were performed as previously described (*15*, *28*, *29*, *63*, *64*). Briefly, after 12 days of organotypic matrix contraction, 40,000 KPC cancer cells were seeded on top of the matrices in a 24-well culture dish (Corning). After allowing for cancer cell attachment and growth for 3 days, matrices were moved to a metal grid with complete DMEM added below, providing an air-liquid interface and chemotactic gradient for cancer cell invasion. The KPC cancer cells were then allowed to invade into the 3D organotypic matrices with medium changes with complete DMEM performed at days 3, 6, and 9. On day 12, matrices were fixed in 10% buffered formalin for downstream H&E analysis. To analyze cancer cell invasion, 600-μm by 500-μm ROIs were obtained from the H&E sections (four ROIs per organotypic matrix and three matrices per condition). Cancer cell invasion was assessed by counting the number of cells invaded into the collagen matrix of >100 μm from the top of the matrix for each ROI using the QuPath (v4.3) cell detection function.

### Unconfined compression analysis

To measure the Young’s modulus (stiffness) of organotypic matrices and orthotopic tumors, we used the Discovery Hybrid Rheometer-3 (TA Instruments) with TRIOS software, as previously achieved (*29*, *47*). For the organotypic experiments, day 12 CAF-contacted matrices were biopsy punched to form a circular sample (8 mm in diameter). These samples were then subjected to a constant linear compression of 10 μm/s, generating a stress/strain curve. The Young’s modulus for the organotypic matrices was determined by calculating the slope of the linear region of the stress/strain curve, accounting for the volume of the sample.

### Scanning electron microscopy of organotypic matrices

Organotypic matrices were fixed in 2% paraformaldehyde and 2.5% glutaraldehyde in 0.1 M sodium cacodylate buffer (pH 7.4), washed with 0.1 M sodium cacodylate buffer, and postfixed in 1% osmium tetroxide and 1.5% potassium ferrocyanide. Following fixation, the matrixes were dehydrated through a graded series of ethanol and then placed in a critical point dryer (Leica CPD 300). Postdrying samples were attached to stubs and sputter-coated in gold. Images were taken using a Jeol JCM-6000 Neoscope scanning electron microscope.

### Subcutaneous tumor model

Subcutaneous tumor models were performed as previously achieved (*15*, *28*, *29*, *64*). A total of 500,000 cells (25% KPC cancer cells and 75% CAF GFP-1 KRAB or CAF B500 NID2 KRAB) were resuspended as single cells in 100 μl of Hanks’ balanced salt solution (HBSS) and maintained on ice before being subcutaneously injected at the left flank of female BALB/c-Fox1nuAusb nude mice under anesthesia [3 liters of isoflurane and O_2_ (1 liter/min); vacuum was used constantly to remove excess of isoflurane; day 0]. Tumor volume was measured every other day using calipers starting on day 3. Tumor volume was calculated by the following formula: (short length × short length × long length)/2. On day 11, mice from the GFP-1 KRAB and B500 NID2 KRAB groups were randomly allocated into two further groups and treated with Abraxane (nab-paclitaxel; 30 mg/kg; Specialised Therapeutics) and gemcitabine (70 mg/kg; MedChemExpress) twice weekly or saline vehicle control via intraperitoneal injection, creating two arms of the study: (i) vehicle and (ii) chemotherapy. When the first mouse in each arm of the study (vehicle or chemotherapy) reached a tumor volume size greater than 350 mm^3^, all mice in that arm were euthanized via cervical dislocation. The subcutaneous tumors were excised and fixed in 10% buffered formalin for downstream IHC analyses.

### Intravital model and imaging

Similar to the subcutaneous model, 500,000 cells (25% KPC eGFP cancer cells and 75% CAF GFP-1 KRAB or CAF B500 NID2 KRAB) were resuspended as single cells in 100 μl of HBSS and maintained on ice before being subcutaneously injected into the left flank of female BALB/c-Fox1nuAusb nude mice under anesthesia [3 liters of isoflurane and O_2_ (1 liter/min); vacuum was used constantly to remove excess of isoflurane; day 0]. Tumor volume was measured every other day using calipers starting on day 3. Tumor volume was calculated by the following formula: (short length × short length × long length)/2. On day 11, mice from the GFP-1 KRAB and B500 NID2 KRAB groups were treated with Abraxane (nab-paclitaxel; 30 mg/kg; Specialised Therapeutics) and gemcitabine (70 mg/kg; MedChemExpress). Mice were treated with gemcitabine and Abraxane chemotherapy on days 11, 14, 18, and 21. On day 23, mice were injected with 100 μl of Qtracker 655 Vascular Labels (1:10 dilution in saline; Thermo Fisher Scientific) via intravenous injection 20 min before imaging, as previously achieved (*15*, *69*–*71*). Mice were terminally anaesthetized using a mix of ketamine (75 mg/kg) and medetomidine (0.75 mg/kg) via intraperitoneal injection, with additional maintenance of anesthesia using 3% isoflurane and O_2_ (1 liter/min), with continuous vacuum to remove excess isoflurane. The eyes were moistened with Lacri-Lube. From here, subcutaneous tumors were surgically exposed using small incisions around the tumor, avoiding the main blood vessels supplying the tumor. Further incisions and blunt dissection were then performed to separate the tumor from the peritoneal and epidermal tissues, creating a skin flap.

Once the subcutaneous tumors were adequately exposed, the mouse was placed on a heated stage at 37°C and maintained under anesthesia for up to 1 hour using 3% isoflurane and O_2_ (1 liter/min), with continuous vacuum to remove excess isoflurane. Images were acquired using a commercially available Leica STELLARIS 8 FALCON DIVE Multiphoton inverted microscope (Garvan ACRF INCITe Centre) with a Leica HC FLUOTAR L 25×/0.95 W VISIR powered by Spectra Physics Insight X3 single and MaiTai eHP Deep See and DMI8 inverted microscope’s external Leica 4Tune spectral Hybrid detectors (Leica Microsystems). SHG signal, GFP, and quantum dot signal were excited at 920 nm, with signal collected at 441 to 481 nm, 560 to 600 nm, and 625 to 665 nm, respectively. For each mouse, at least four representative ROIs of 512 pixels by 512 pixels were imaged over a 3D z-stack (2.52-μm step size). For representative movies of blood flow, ROIs of 512 pixels by 512 pixels were captured with a frame length of 1 s and 279 ms.

### Blood vessel analysis

3D Quantitative analysis of cell morphology was performed using Imaris (Bitplane AG, Switzerland), ImageJ (NIH), and VesselVio (*73*) (fig. S13). First, a threshold was applied to the quantum dot channel and reconstructed as a 3D surface object on Imaris. From here, a mask was applied to the generated quantum dot 3D surface object and exported as a Tagged Image File (TIF) z-stack. The TIF z-stack was then binarized on ImageJ (FIJI, NIH). The binary z-stack was then subjected to the fill holes and median subtraction (radius, 10 μm) filters on ImageJ. From here, the binarized and filtered TIF z-stacks were analyzed using VesselVio (*73*) using the following settings in Table 1.

**Table 1.** Analysis settings used on VesselVio software (*73*).

Parameter	Setting
Unit	μm
Anisotropic image resolution (*xyz*)	1.21 μm × 1.21 μm × 2.52 μm
Analysis dimensions	3D
Filter isolated segments shorter than	15 μm
Prune end-point segments shorter than	40 μm

From here, the total volume, total surface area, and mean segment radius were obtained for each TIF z-stack. The volume and surface area values were normalized to the *z* depth of the individual TIF z-stack in micrometers. Using the “Visualize” tool on the VesselVio software (*73*), representative images of the skeletonization and segmentation of blood vessels were prepared using the same settings as above (Table 1 and fig. S13) (*73*).

### Orthotopic tumor models

Orthotopic tumor models were performed as previously achieved (*15*, *28*, *29*, *64*). Briefly, 200 cells (25% KPC luciferase cancer cells and 75% CAF GFP-1 KRAB or CAF B500 NID2 KRAB) were resuspended as single cells in a 50-μl 1:1 mixture of Matrigel and HBSS, which was maintained on ice before being orthotopically injected into the pancreas of female NSG mice (day 0). To perform the surgery, the fur was removed, and the mouse was anesthetized [3 liters of isoflurane and O_2_ (1 liter/min); vacuum was used constantly to remove excess of isoflurane) and skin-sterilized. Once anesthetized, the mouse was placed in the right lateral decubitus position, and the eyes were moistened with Lacri-Lube. From here, a subcostal ~8-mm incision was made in the skin at the left flank. After blunt dissection, a 5-mm incision was made in the peritoneal wall to expose the pancreas. Light tension was applied to the pancreatic tissue by holding the splenorenal ligament with forceps. The 50-μl cell Matrigel:HBSS mixture was then injected into the pancreas using a 0.3-ml insulin syringe, forming a bubble. The pancreas was then placed back into the body cavity, with the peritoneal wall sutured (5-0 vicryl) to close the incision. Topical bupivacaine (8 mg/kg) was applied to the suture site, and then the skin was closed with wound clips. Mice were administered buprenorphine (0.075 mg/kg) subcutaneously for analgesia and monitored daily for 7 days and until presurgery weight was achieved. After this, wound clips were removed from all mice. Mice were then assessed for an IVIS signal. To do this, mice were administered with luciferin (150 mg/kg; Gold Biotechnology) via intraperitoneal injection, and signal was acquired on an IVIS Spectrum with Living Image software (PerkinElmer, USA) with open filters and small binning. Mice with a detectable IVIS signal from the GFP-1 KRAB and B500 NID2 KRAB groups were randomly allocated into two further groups and treated with Abraxane (nab-paclitaxel; 30 mg/kg; Specialised Therapeutics) and gemcitabine (70 mg/kg; MedChemExpress) or saline vehicle control twice weekly, creating two arms of the study: (i) vehicle and (ii) chemotherapy. For the survival orthotopic study (Fig. 6), all mice were treated with chemotherapy or vehicle control until study end point, after which the mouse was euthanized via cervical dislocation. Study end point was determined by development of gross ascites, a weight loss of >20% from top weight, a weight loss of >10% overnight, determination of a body condition score of ≤2, prolonged hunching, prolonged subdued behavior, prolonged reduction of movement, or signs of systemic illness. For the timed end-point orthotopic study (Fig. 7), when the first mouse in each arm of the study (vehicle or chemotherapy) reached study end point, all mice in that arm were euthanized via cervical dislocation. The orthotopic tumors and livers were excised and fixed in 10% buffered formalin for downstream IHC analyses. Liver metastases in the orthotopic timed end-point experiment were analyzed using H&E sections of formalin-fixed, paraffin-embedded (FFPE) liver lobes using the QuPath (v4.3) classifier function (fig. S15).

### Western blotting

Cells were lysed in radioimmunoprecipitation assay (RIPA) buffer (50 mM Hepes, 1% Triton X-100, 0.5% SDC, 0.1% SDS, 0.5 mM EDTA, and 50 mM NaF) supplemented with phosphatase inhibitor cocktail tablet (PhosSTOP, Roche) and protease inhibitor cocktail tablet (cOmplete Ultra, Roche). For the NID2 and β-actin Western blots, 18.75 μg of protein per lane was separated by 10% acrylamide bis-tris gel electrophoresis and then transferred onto a polyvinylidene difluoride membrane (Immobilon-P, Millipore). Membranes were blocked with 5% bovine serum albumin (BSA; Sigma-Aldrich) diluted in tris-buffered saline supplemented with 0.1% Tween 20 (TBST). From here, membranes were incubated in primary antibodies (1:1000 anti-mouse NID2 rabbit antisera, AB_2801613, supplied by author T.S.; 1:10,000 β-actin, mouse monoclonal antibody, A5441, Sigma-Aldrich) overnight at 4°C with shaking. From here, membranes were incubated with horseradish peroxidase–conjugated secondary antibodies [rabbit (NID2) and mouse (β-actin); both 1:10,000 diluted in 1% BSA in TBST; GE Healthcare] for 1 hour at room temperature with shaking. Signal was detected using ultra–enhanced chemiluminescence (uECL; NID2) or ECL (β-actin) reagent and imaged (Fusion FX, Vilber). For the pMYPT1, pMLC2, and glyceraldehyde-3-phosphate dehydrogenase (GAPDH) Western blots, 20 μg of protein per lane was separated by 4 to 12% acrylamide bis-tris gel electrophoresis and then transferred onto a polyvinylidene difluoride membrane. Membranes were blocked with 5% BSA diluted in TBST. From here, membranes were incubated in primary antibodies (1:500 anti-mouse pMYPT1 T696; rabbit polyclonal, ABS45, Merck Millipore; 1:1000 anti-mouse pMLC2 S19; rabbit polyclonal, 3671, Cell Signaling Technology; 1:10,000 GAPDH; mouse monoclonal antibody, ACR001P, Sigma-Aldrich) overnight at 4°C with shaking. From here, membranes were incubated with horseradish peroxidase–conjugated secondary antibodies (rabbit, pMPYT1, pMLC2; 1:5000; mouse, GAPDH; 1:10,000) and diluted in 1% BSA in TBST for 1 hour at room temperature with shaking. Signal was detected using uECL (pMYPT1 and pMLC2) or ECL (GAPDH) reagent and imaged (Fusion FX, Vilber). Densitometry of detected bands was calculated using ImageJ (FIJI, NIH).

### Reverse transcriptase quantitative polymerase chain reaction

RNA was isolated using the QIAGEN RNeasy Mini Kit as per the manufacturer’s instructions. cDNA synthesis was performed using the Roche Transcriptor First Strand cDNA Synthesis Kit from 1 μg of total RNA as per the manufacturer’s instructions and was diluted 1:5 with nuclease-free H_2_O (QIAGEN). RT-qPCR experiments were performed using the Roche Universal Probe Library (UPL) System on the Roche LightCycler 480 (Roche). *NID2* (exon 1/2) expression was detected with the following primers (forward, GTGAAGCTGGCAATACCCCT; reverse, GTGTCGATGTCAGCCAGGAA; with UPL probe #105). *NID2* (exon 6/7) expression was detected with the following primers (forward, GAATGGCTTCAGCCTCACAG; reverse, CGGCAGTTTGAGTGATACGA; with UPL probe #55). The expression of *GAPDH* was detected with the following primers (forward, GGGTTCCTATAAATACGGACTGC; reverse, CCATT-TTGTCTACGGGACGA; with UPL probe #52). *COL1A1* experiments were performed on QuantStudio 7 (Thermo Fisher Scientific) using the following probe sets from Applied Biosciences: *COL1A1*, Mm00801666_g1; *GAPDH*, Mm99999915_g1. Relative mRNA expression for each transcript of interest was normalized to *GAPDH* mRNA expression and then quantified using double Δ*C*_t_ comparisons for each biological replicate.

### Immunofluorescence

Twelve-millimeter glass coverslips were first coated with 2% gelatin in PBS and incubated for 1 hour at 37°C. Coverslips were washed twice with PBS and fixed with 10% buffered formalin for 30 min at room temperature. After washing the coverslips twice with PBS, the coverslips were quenched with 1 M sterile glycine in PBS for 20 min at room temperature. Coverslips were then incubated with a 1:1 solution of FBS and PBS for 5 min at room temperature. Coverslips were then incubated in complete DMEM for 30 min at 37°C. After washing coverslips with PBS twice, KPC CAFs were seeded onto the gelatin-coated glass coverslips in 24-well cell culture plate (Costar, Corning) at 20,000 cells per well in complete DMEM at 37°C with 20% O_2_ and 5% CO_2_ for 24 hours. Specimens were fixed with 4% paraformaldehyde (ProSciTech) for 15 min at room temperature. Specimens were washed three times with PBS and permeabilized with 0.1% Triton X-100 in PBS for 10 min. Specimens were washed three times and blocked with 1% BSA + 0.02% glycine in PBS for 1 hour at room temperature. After washing three times with PBS, specimens were incubated with 1:100 anti-mouse pMYPT1 T696 (rabbit polyclonal, ABS45, Merck Millipore) or 1:100 anti-mouse pMLC2 S19 (rabbit polyclonal, 3671, Cell Signaling Technology) in PBS overnight at 4°C in a humidified chamber. After primary antibody incubation, specimens were washed three times with PBS and incubated in 1:500 anti-rabbit Cy3 (711-165-152, Jackson ImmunoResearch), 1:300 488 phalloidin (Invitrogen), and 4,6-diamidino-2-phenylindole (DAPI; 0.2 μg/ml; Invitrogen) for 1 hour at room temperature away from direct light. After washing three times in PBS, specimens were mounted onto glass slides using ProLong Diamond (Thermo Fisher Scientific). Mounted specimens were stored in the dark at 4°C until imaging. Specimens were imaged using a Leica DMI 6000 SP8 microscope equipped with a 40× oil immersion lens (40×/1.30 oil; Garvan ACRF INCITe Centre). DAPI, Alexa Fluor 488, and Cy3 fluorescence were excited with a 405-nm diode laser, 488-nm optically pumped semiconductor laser (OPSL) laser, and 552-nm OPSL laser, respectively. Emission was detected on an internal photomultiplier tube with a variable band-pass filter tuned to 594 to 649 nm. At least six representative ROIs were acquired. All ROIs were 512 pixels by 512 pixels in size.

### IF quantification

The Cellpose plugin for ImageJ (FIJI, NIH) was used to segment the cells using the F-actin channel. The nucleus region was segmented using the DAPI channel and Huang2 thresholding within the cells. The cytoplasmic region was segmented by creating an XOR operation of the cell segment with the nucleus segment. The cell mean intensity for pMLC2 and pMYPT1 was calculated by averaging the nuclear and cytoplasmic compartments. The cell mean intensity of F-actin was calculated by calculating the cell segment of the F-actin channel. The F-actin data were generated by using the specimens stained for DAPI, pMLC2, and F-actin.

### Histology and IHC

Histology and IHC FFPE organotypic matrices and tumor tissues were processed on a Leica Peloris following standard tissue processing protocols. FFPE specimens were sectioned at 4 μm using a Leica RM2235 microtome. Sections were placed on a plain glass slide for H&E staining or a positively charged slide for IHC, which was allowed to incubate for 2 hours in a 60°C oven for maximum adhesion. Sections were deparaffinized and stained following standard H&E procedures on the Leica ST5010 Autostainer XL with hematoxylin [Hematoxylin Harris nontoxic (acidified), Australian Biostain] and eosin (Eosin Phloxine alcoholic 1%, Australian Biostain). 

#### BOND RX autostainer

For IHC, all staining was performed on the Leica BOND RX autostainer. Sections were first dewaxed using BOND Dewax Solution (AR2992, Leica). Epitope retrieval was performed by heating slides to 93°C for organotypic matrices and 100°C for tumor sections using epitope retrieval solution 2 (pH 9; AR9640, Leica). The details for epitope retrieval time, primary antibody dilution, and incubation time are provided in Table 2. For NID2 IHC, the Leica BOND Polymer Refine Detection (reference: DS9800) protocol was used. Slides were counterstained with hematoxylin and coverslipped on a Leica Coverslipper (CV5030). H&E and IHC slides were scanned using an Aperio slide scanner (Leica), NanoZoomer S60 Digital slide scanner (Hamamatsu), or Slideview VS200 slide scanner (Olympus). The 3,3′-diaminobenzidine coverage in terms of positive pixels (NID1, NID2, and CD31) or positive cells (Ki-67 and CC3) was analyzed using the QuPath (v4.3) positive pixel and positive cell detection function, respectively.

**Table 2.** IHC primary antibody details, stained using the Leica BOND RX Autostainer.

Antibody	Company	Catalog number	Epitope retrieval (min)	Dilution	Incubation time (min)
NID1	Invitrogen	MA1-06501	30	1:100 (organotypic matrix)	60
				1:200 (tumor tissue)	
NID2	Takako Sasaki (author)	AB_2801613	30	1:2000 (organotypic matrix)	60
				1:1000 (tumor tissue)	
CD31	Dianova	DIA-310	40	1:100 (tumor tissue)	60
Ki-67	Epredia	RM9106S1	30	1:500 (organotypic matrix)	60
Cleaved caspase 3 (CC3)	Cell Signaling Technology	9661	20	1:200 (organotypic matrix)	60

### Picrosirius Red staining and polarized light imaging

For Picrosirius Red staining of formalin-fixed organotypic matrices and tumor tissues, 4-μm sections were cut and adhered to Superfrost Plus slides. Slides were dewaxed using the Leica ST5010 Autostainer XL and then stained manually with 0.02% phosphomolybdic acid and 0.1% Picrosirius Red (Australian Biostain) for fibrillar collagens. After rinsing in acidified water and dehydration in graded ethanol, slides were coverslipped using a Leica Coverslipper (CV5030). Picrosirius Red slides were scanned using a Slideview VS200 slide scanner (Olympus) using bright-field and polarized (birefringence) mode. Analysis of Picrosirius Red and total birefringence coverage was analyzed using ImageJ, as previously achieved (*15*, *28*, *29*, *64*). For the red-orange, yellow, and green birefringence coverages, images were analyzed using ImageJ, as previously achieved (*15*, *28*, *29*). Briefly, hue-saturation balance thresholding was applied (high birefringence/red-orange 0 > H < 27 | 0 > S < 255 | 70 > B < 255, medium birefringence/yellow 28 > H < 47 | 0 > S < 255 | 70 > B < 255, and low birefringence/green 48 > H < 140 | 0 > S < 255 | 70 > B < 255).

### SHG imaging of CDMs and 3D organotypic matrices

SHG imaging was performed as previously described (*15*, *28*, *29*, *64*). Briefly, CDMs were imaged unfixed on day 7, and organotypic matrices were imaged unfixed on day 12. SHG signal was detected using a 25× water immersion objective (HCX IRAPO L 25×/0.95 water) on an inverted Leica DMI 6000 SP8 confocal microscope (Garvan ACRF INCITe Centre). Excitation was achieved using a Ti:sapphire femtosecond laser (Coherent Chameleon Ultra II) at 80 MHz and tuned to a wavelength of 880 nm. The intensity was recorded with RLD HyD detectors at 440/20 nm. For CDMs, four to eight z-stack representative ROIs were acquired per well per condition, and for organotypic matrices, eight z-stack ROIs were acquired per matrix per condition. All ROIs were 512 pixels by 512 pixels in size with a *z*-step size of 1.51 μm for CDMs and 2.52 μm for organotypic matrices. The intensity of the SHG signal was quantified using MATLAB (MathWorks, USA). Representative images of maximum intensity projections are shown.

### Tissue IF and colocalization analysis

Multiplex IF staining of NID2, CD31, and CD146 was performed on a subset of OCT-embedded (Scigen) KPC tumors from the MS cohort (table S1). Collagen IV and laminin IF staining was performed on OCT-embedded (Scigen) GFP-1 KRAB and B500 NID2 KRAB subcutaneous tumors from the vehicle arm of the study (fig. S12). Seven-micrometer OCT-embedded sections were cut and adhered to Superfrost Plus slides for IF staining. Tumors sections were thawed at room temperature for 20 min and then fixed in 4% paraformaldehyde in PBS for 15 min. Sections were washed three times in PBS and permeabilized with 0.05% Triton X-100 for 10 min. The M.O.M. (Mouse on Mouse) Immunodetection Kit, Basic (Vector Laboratories), was used from this point onward. Sections were blocked in 150 μl of M.O.M. blocking buffer with 5% normal donkey serum (Jackson ImmunoResearch) for 1 hour in a humidified chamber. Sections were washed twice in PBS and incubated in M.O.M. diluent for 5 min. For the KPC tumors, a primary antibody cocktail diluted in M.O.M. diluent (see Table 3) was prepared, with tissues incubated at room temperature overnight using 60 μl per tumor section. For the subcutaneous tumors, tissues were incubated in 1:200 collagen IV (rabbit polyclonal, ab6586, Abcam) or 1:200 laminin (rabbit polyclonal, ab2034, Merck Millipore) antibody diluted in M.O.M. diluent at room temperature overnight using 80 μl per tumor section. Sections were washed twice in PBS. For the KPC tumors, a fluorescently labeled secondary antibody cocktail (see Table 3) diluted in M.O.M. diluent was applied to each tumor and incubated for 1 hour at room temperature in a humidified chamber protected from light. For the subcutaneous tumors, tissues were incubated in 1:500 goat anti-rabbit immunoglobulin G (IgG) (H + L) cross-adsorbed secondary antibody Alexa Fluor 647 (Invitrogen, Life Technologies) diluted in M.O.M. diluent for 1 hour at room temperature in a humidified chamber protected from light. Sections were washed twice in PBS. For the subcutaneous tumors, tissues were incubated in DAPI (1 μg/ml; D5942, Sigma-Aldrich) diluted in M.O.M. diluent for 5 min. Tissue sections were then coverslipped using ProLong Diamond mounting agent (Thermo Fisher Scientific). KPC tumor sections were imaged using a Leica DMI 6000 SP8 microscope equipped with a 20× water immersion lens (20×/0.70 immersion lens; Garvan ACRF INCITe Centre), while subcutaneous tumors were imaged with a 40× oil immersion lens (40×/1.30 oil; Garvan ACRF INCITe Centre). For the KPC tumors, Alexa Fluor 488, Alexa Fluor 594, and Alexa Fluor 647 fluorescences were excited with a 488-nm OPSL laser, 552-nm OPSL laser, and 638-nm diode laser, respectively. For the subcutaneous tumors, DAPI and Alexa Fluor 647 were excited with a 405-nm diode laser and a 638-nm diode laser, respectively. Emission was detected on an internal photo multiplier tube with a variable band-pass filter tuned to 594 to 649 nm. For both the KPC and subcutaneous tumor sections, at least eight representative ROIs were acquired. All ROIs were 512 pixels by 512 pixels in size. For the KPC tumors, colocalization of CD31 + NID2 and CD146 + NID2 was identified and quantified using the confined displacement algorithm ImageJ plugin. Briefly, areas with signals from both channels (CD31 + NID2 and CD146 + NID2, respectively) are identified and a threshold applied according to Costes autothreshold determination. In a second step, the correlation between colocalized channels is calculated. Colocalization was measured using the Pearson’s correlation coefficient.

**Table 3.** Primary and secondary antibodies used for tissue IF staining.

Antibody	Primary or secondary antibody	Company	Catalog number	Host	Reactivity	Dilution
NID2	Primary	Takako Sasaki (author)	AB_2801613	Rabbit polyclonal IgG	Anti-mouse	1:200
CD31	Primary	Dianova	DIA-310	Rat monoclonal IgG2a	Anti-mouse	1:100
CD146	Primary	R&D Systems	AF6106	Polyclonal sheep IgG	Anti-mouse	1:200
Collagen IV	Primary	Abcam	Ab6586	Rabbit polyclonal IgG	Anti-mouse	1:200
Laminin	Primary	Merck Millipore	Ab2034	Rabbit polyclonal IgG	Anti-mouse	1:200
Alexa Fluor 647	Secondary	Thermo Fisher Scientific	A-21244	Goat IgG	Anti-rabbit	1:500
Alexa Fluor 488	Secondary	Thermo Fisher Scientific	A-11006	Goat IgG	Anti-rat	1:500
Alexa Fluor 594	Secondary	Thermo Fisher Scientific	A-11016	Donkey IgG	Anti-sheep	1:500

## Supplementary Materials

### This PDF file includes:

Figs. S1 to S15 Legends for tables S1 to S7 Legends for movies S1 to S4 Consortium members of the Australian Pancreatic Cancer Genome Initiative (APGI) Consortium members of the Avner Australian Pancreatic Cancer Matrix Atlas (APMA)

## References

[1] R. L. Siegel, K. D. Miller, N. S. Wagle, A. Jemal, Cancer statistics, 2023. CA Cancer J. Clin. 73, 17–48 (2023). 36633525 10.3322/caac.21763

[2] Z. I. Hu, E. M. O’Reilly, Therapeutic developments in pancreatic cancer. Nat. Rev. Gastroenterol. Hepatol. 21, 7–24 (2024). 37798442 10.1038/s41575-023-00840-w

[3] Cancer Genome Atlas Research Network, Integrated genomic characterization of pancreatic ductal adenocarcinoma. Cancer Cell 32, 185–203.e13 (2017). 28810144 10.1016/j.ccell.2017.07.007 PMC5964983

[4] B. A. Pereira, C. Vennin, M. Papanicolaou, C. R. Chambers, D. Herrmann, J. P. Morton, T. R. Cox, P. Timpson, CAF subpopulations: A new reservoir of stromal targets in pancreatic cancer. Trends Cancer 5, 724–741 (2019). 31735290 10.1016/j.trecan.2019.09.010

[5] E. Sahai, I. Astsaturov, E. Cukierman, D. G. DeNardo, M. Egeblad, R. M. Evans, D. Fearon, F. R. Greten, S. R. Hingorani, T. Hunter, R. O. Hynes, R. K. Jain, T. Janowitz, C. Jorgensen, A. C. Kimmelman, M. G. Kolonin, R. G. Maki, R. S. Powers, E. Puré, D. C. Ramirez, R. Scherz-Shouval, M. H. Sherman, S. Stewart, T. D. Tlsty, D. A. Tuveson, F. M. Watt, V. Weaver, A. T. Weeraratna, Z. Werb, A framework for advancing our understanding of cancer-associated fibroblasts. Nat. Rev. Cancer 20, 174–186 (2020). 31980749 10.1038/s41568-019-0238-1 PMC7046529

[6] E. Helms, M. K. Onate, M. H. Sherman, Fibroblast heterogeneity in the pancreatic tumor microenvironment. Cancer Discov. 10, 648–656 (2020). 32014869 10.1158/2159-8290.CD-19-1353 PMC8261791

[7] C. Vennin, K. J. Murphy, J. P. Morton, T. R. Cox, M. Pajic, P. Timpson, Reshaping the tumor stroma for treatment of pancreatic cancer. Gastroenterology 154, 820–838 (2018). 29287624 10.1053/j.gastro.2017.11.280

[8] E. Elyada, M. Bolisetty, P. Laise, W. F. Flynn, E. T. Courtois, R. A. Burkhart, J. A. Teinor, P. Belleau, G. Biffi, M. S. Lucito, S. Sivajothi, T. D. Armstrong, D. D. Engle, K. H. Yu, Y. Hao, C. L. Wolfgang, Y. Park, J. Preall, E. M. Jaffee, A. Califano, P. Robson, D. A. Tuveson, Cross-species single-cell analysis of pancreatic ductal adenocarcinoma reveals antigen-presenting cancer-associated fibroblasts. Cancer Discov. 9, 1102–1123 (2019). 31197017 10.1158/2159-8290.CD-19-0094 PMC6727976

[9] G. Biffi, T. E. Oni, B. Spielman, Y. Hao, E. Elyada, Y. Park, J. Preall, D. A. Tuveson, IL1-induced JAK/STAT signaling is antagonized by TGFβ to shape CAF heterogeneity in pancreatic ductal adenocarcinoma. Cancer Discov. 9, 282–301 (2019). 30366930 10.1158/2159-8290.CD-18-0710 PMC6368881

[10] C. X. Dominguez, S. Müller, S. Keerthivasan, H. Koeppen, J. Hung, S. Gierke, B. Breart, O. Foreman, T. W. Bainbridge, A. Castiglioni, Y. Senbabaoglu, Z. Modrusan, Y. Liang, M. R. Junttila, C. Klijn, R. Bourgon, S. J. Turley, Single-cell RNA sequencing reveals stromal evolution into LRRC15^+^ myofibroblasts as a determinant of patient response to cancer immunotherapy. Cancer Discov. 10, 232–253 (2020). 31699795 10.1158/2159-8290.CD-19-0644

[11] H. Huang, Z. Wang, Y. Zhang, R. N. Pradhan, D. Ganguly, R. Chandra, G. Murimwa, S. Wright, X. Gu, R. Maddipati, S. Müller, S. J. Turley, R. A. Brekken, Mesothelial cell-derived antigen-presenting cancer-associated fibroblasts induce expansion of regulatory T cells in pancreatic cancer. Cancer Cell 40, 656–673.e7 (2022). 35523176 10.1016/j.ccell.2022.04.011 PMC9197998

[12] C. Hutton, F. Heider, A. Blanco-Gomez, A. Banyard, A. Kononov, X. Zhang, S. Karim, V. Paulus-Hock, D. Watt, N. Steele, S. Kemp, E. K. J. Hogg, J. Kelly, R. F. Jackstadt, F. Lopes, M. Menotti, L. Chisholm, A. Lamarca, J. Valle, O. J. Sansom, C. Springer, A. Malliri, R. Marais, M. Pasca di Magliano, S. Zelenay, J. P. Morton, C. Jørgensen, Single-cell analysis defines a pancreatic fibroblast lineage that supports anti-tumor immunity. Cancer Cell 39, 1227–1244.e20 (2021). 34297917 10.1016/j.ccell.2021.06.017 PMC8443274

[13] P. E. Garcia, M. Adoumie, E. C. Kim, Y. Zhang, M. K. Scales, Y. S. el-Tawil, A. Z. Shaikh, H. J. Wen, F. Bednar, B. L. Allen, D. M. Wellik, H. C. Crawford, M. Pasca di Magliano, Differential contribution of pancreatic fibroblast subsets to the pancreatic cancer stroma. Cell. Mol. Gastroenterol. Hepatol. 10, 581–599 (2020). 32454112 10.1016/j.jcmgh.2020.05.004 PMC7399194

[14] D. Kpeglo, M. D. G. Hughes, L. Dougan, M. Haddrick, M. A. Knowles, S. D. Evans, S. A. Peyman, Modeling the mechanical stiffness of pancreatic ductal adenocarcinoma. Matrix Biol. Plus 14, 100109 (2022). 35399702 10.1016/j.mbplus.2022.100109 PMC8990173

[15] C. Vennin, V. T. Chin, S. C. Warren, M. C. Lucas, D. Herrmann, A. Magenau, P. Melenec, S. N. Walters, G. del Monte-Nieto, J. R. W. Conway, M. Nobis, A. H. Allam, R. A. McCloy, N. Currey, M. Pinese, A. Boulghourjian, A. Zaratzian, A. A. S. Adam, C. Heu, A. M. Nagrial, A. Chou, A. Steinmann, A. Drury, D. Froio, M. Giry-Laterriere, N. L. E. Harris, T. Phan, R. Jain, W. Weninger, E. J. McGhee, R. Whan, A. L. Johns, J. S. Samra, L. Chantrill, A. J. Gill, M. Kohonen-Corish, R. P. Harvey, A. V. Biankin; Australian Pancreatic Cancer Genome Initiative (APGI), T. R. J. Evans, K. I. Anderson, S. T. Grey, C. J. Ormandy, D. Gallego-Ortega, Y. Wang, M. S. Samuel, O. J. Sansom, A. Burgess, T. R. Cox, J. P. Morton, M. Pajic, P. Timpson, Transient tissue priming via ROCK inhibition uncouples pancreatic cancer progression, sensitivity to chemotherapy, and metastasis. Sci. Transl. Med. 9, eaai8504 (2017). 28381539 10.1126/scitranslmed.aai8504 PMC5777504

[16] H. Laklai, Y. A. Miroshnikova, M. W. Pickup, E. A. Collisson, G. E. Kim, A. S. Barrett, R. C. Hill, J. N. Lakins, D. D. Schlaepfer, J. K. Mouw, V. S. LeBleu, N. Roy, S. V. Novitskiy, J. S. Johansen, V. Poli, R. Kalluri, C. A. Iacobuzio-Donahue, L. D. Wood, M. Hebrok, K. Hansen, H. L. Moses, V. M. Weaver, Genotype tunes pancreatic ductal adenocarcinoma tissue tension to induce matricellular fibrosis and tumor progression. Nat. Med. 22, 497–505 (2016). 27089513 10.1038/nm.4082 PMC4860133

[17] R. J. Torphy, Z. Wang, A. True-Yasaki, K. E. Volmar, N. Rashid, B. Yeh, J. S. Johansen, M. A. Hollingsworth, J. J. Yeh, E. A. Collisson, Stromal content is correlated with tissue site, contrast retention, and survival in pancreatic adenocarcinoma. JCO Precis. Oncol. 2018, 1–12 (2018). 10.1200/PO.17.00121 PMC6262879 30506016

[18] C. C. DuFort, K. E. DelGiorno, M. A. Carlson, R. J. Osgood, C. Zhao, Z. Huang, C. B. Thompson, R. J. Connor, C. D. Thanos, J. Scott Brockenbrough, P. P. Provenzano, G. I. Frost, H. Michael Shepard, S. R. Hingorani, Interstitial pressure in pancreatic ductal adenocarcinoma is dominated by a gel-fluid phase. Biophys. J. 110, 2106–2119 (2016). 27166818 10.1016/j.bpj.2016.03.040 PMC4939548

[19] P. P. Provenzano, C. Cuevas, A. E. Chang, V. K. Goel, D. D. von Hoff, S. R. Hingorani, Enzymatic targeting of the stroma ablates physical barriers to treatment of pancreatic ductal adenocarcinoma. Cancer Cell 21, 418–429 (2012). 22439937 10.1016/j.ccr.2012.01.007 PMC3371414

[20] M. A. Jacobetz, D. S. Chan, A. Neesse, T. E. Bapiro, N. Cook, K. K. Frese, C. Feig, T. Nakagawa, M. E. Caldwell, H. I. Zecchini, M. P. Lolkema, P. Jiang, A. Kultti, C. B. Thompson, D. C. Maneval, D. I. Jodrell, G. I. Frost, H. M. Shepard, J. N. Skepper, D. A. Tuveson, Hyaluronan impairs vascular function and drug delivery in a mouse model of pancreatic cancer. Gut 62, 112–120 (2013). 22466618 10.1136/gutjnl-2012-302529 PMC3551211

[21] I. Peran, S. Dakshanamurthy, M. D. McCoy, A. Mavropoulos, B. Allo, A. Sebastian, N. R. Hum, S. C. Sprague, K. A. Martin, M. J. Pishvaian, E. E. Vietsch, A. Wellstein, M. B. Atkins, L. M. Weiner, A. A. Quong, G. G. Loots, S. S. Yoo, S. Assefnia, S. W. Byers, Cadherin 11 promotes immunosuppression and extracellular matrix deposition to support growth of pancreatic tumors and resistance to gemcitabine in mice. Gastroenterology 160, 1359–1372.e13 (2021). 33307028 10.1053/j.gastro.2020.11.044 PMC7956114

[22] H. Jiang, S. Hegde, B. L. Knolhoff, Y. Zhu, J. M. Herndon, M. A. Meyer, T. M. Nywening, W. G. Hawkins, I. M. Shapiro, D. T. Weaver, J. A. Pachter, A. Wang-Gillam, D. G. DeNardo, Targeting focal adhesion kinase renders pancreatic cancers responsive to checkpoint immunotherapy. Nat. Med. 22, 851–860 (2016). 27376576 10.1038/nm.4123 PMC4935930

[23] C. J. Garcia Garcia, Y. Huang, N. R. Fuentes, M. C. Turner, M. E. Monberg, D. Lin, N. D. Nguyen, T. N. Fujimoto, J. Zhao, J. J. Lee, V. Bernard, M. Yu, A. M. Delahoussaye, I. Jimenez Sacarello, E. G. Caggiano, J. L. Phan, A. Deorukhkar, J. M. Molkentine, D. Saur, A. Maitra, C. M. Taniguchi, Stromal HIF2 regulates immune suppression in the pancreatic cancer microenvironment. Gastroenterology 162, 2018–2031 (2022). 35216965 10.1053/j.gastro.2022.02.024 PMC9278556

[24] A. D. Rhim, P. E. Oberstein, D. H. Thomas, E. T. Mirek, C. F. Palermo, S. A. Sastra, E. N. Dekleva, T. Saunders, C. P. Becerra, I. W. Tattersall, C. B. Westphalen, J. Kitajewski, M. G. Fernandez-Barrena, M. E. Fernandez-Zapico, C. Iacobuzio-Donahue, K. P. Olive, B. Z. Stanger, Stromal elements act to restrain, rather than support, pancreatic ductal adenocarcinoma. Cancer Cell 25, 735–747 (2014). 24856585 10.1016/j.ccr.2014.04.021 PMC4096698

[25] B. C. Özdemir, T. Pentcheva-Hoang, J. L. Carstens, X. Zheng, C. C. Wu, T. R. Simpson, H. Laklai, H. Sugimoto, C. Kahlert, S. V. Novitskiy, A. de Jesus-Acosta, P. Sharma, P. Heidari, U. Mahmood, L. Chin, H. L. Moses, V. M. Weaver, A. Maitra, J. P. Allison, V. S. LeBleu, R. Kalluri, Depletion of carcinoma-associated fibroblasts and fibrosis induces immunosuppression and accelerates pancreas cancer with reduced survival. Cancer Cell 25, 719–734 (2014). 24856586 10.1016/j.ccr.2014.04.005 PMC4180632

[26] Y. Chen, J. Kim, S. Yang, H. Wang, C. J. Wu, H. Sugimoto, V. S. LeBleu, R. Kalluri, Type I collagen deletion in αSMA^+^ myofibroblasts augments immune suppression and accelerates progression of pancreatic cancer. Cancer Cell 39, 548–565.e6 (2021). 33667385 10.1016/j.ccell.2021.02.007 PMC8423173

[27] H. Jiang, R. J. Torphy, K. Steiger, H. Hongo, A. J. Ritchie, M. Kriegsmann, D. Horst, S. E. Umetsu, N. M. Joseph, K. McGregor, M. J. Pishvaian, E. M. Blais, B. Lu, M. Li, M. Hollingsworth, C. Stashko, K. Volmar, J. J. Yeh, V. M. Weaver, Z. J. Wang, M. A. Tempero, W. Weichert, E. A. Collisson, Pancreatic ductal adenocarcinoma progression is restrained by stromal matrix. J. Clin. Invest. 130, 4704–4709 (2020). 32749238 10.1172/JCI136760 PMC7456216

[28] C. Vennin, P. Mélénec, R. Rouet, M. Nobis, A. S. Cazet, K. J. Murphy, D. Herrmann, D. A. Reed, M. C. Lucas, S. C. Warren, Z. Elgundi, M. Pinese, G. Kalna, D. Roden, M. Samuel, A. Zaratzian, S. T. Grey, A. D. Silva, W. Leung; Australian Pancreatic Genome Initiative (APGI), S. Mathivanan, Y. Wang, A. W. Braithwaite, D. Christ, A. Benda, A. Parkin, P. A. Phillips, J. M. Whitelock, A. J. Gill, O. J. Sansom, D. R. Croucher, B. L. Parker, M. Pajic, J. P. Morton, T. R. Cox, P. Timpson, CAF hierarchy driven by pancreatic cancer cell p53-status creates a pro-metastatic and chemoresistant environment via perlecan. Nat. Commun. 10, 3637 (2019). 31406163 10.1038/s41467-019-10968-6 PMC6691013

[29] K. J. Murphy, D. A. Reed, C. Vennin, J. R. W. Conway, M. Nobis, J. X. Yin, C. R. Chambers, B. A. Pereira, V. Lee, E. C. Filipe, M. Trpceski, S. Ritchie, M. C. Lucas, S. C. Warren, J. N. Skhinas, A. Magenau, X. L. Metcalf, J. Stoehr, G. Major, A. Parkin, R. Bidanel, R. J. Lyons, A. Zaratzian, M. Tayao, A. da Silva, L. Abdulkhalek; Australian Pancreatic Genome Initiative (APGI); Australian Pancreatic Cancer Matrix Atlas (APMA), A. J. Gill, A. L. Johns, A. V. Biankin, J. Samra, S. M. Grimmond, A. Chou, J. G. Goetz, M. S. Samuel, J. G. Lyons, A. Burgess, C. E. Caldon, L. G. Horvath, R. J. Daly, N. Gadegaard, Y. Wang, O. J. Sansom, J. P. Morton, T. R. Cox, M. Pajic, D. Herrmann, P. Timpson, Intravital imaging technology guides FAK-mediated priming in pancreatic cancer precision medicine according to Merlin status. Sci. Adv. 7, eabh0363 (2021). 34586840 10.1126/sciadv.abh0363 PMC8480933

[30] J. Datta, X. Dai, A. Bianchi, I. de Castro Silva, S. Mehra, V. T. Garrido, P. Lamichhane, S. P. Singh, Z. Zhou, A. R. Dosch, F. Messaggio, Y. Ban, O. Umland, P. J. Hosein, N. S. Nagathihalli, N. B. Merchant, Combined MEK and STAT3 inhibition uncovers stromal plasticity by enriching for cancer-associated fibroblasts with mesenchymal stem cell-like features to overcome immunotherapy resistance in pancreatic cancer. Gastroenterology 163, 1593–1612 (2022). 35948109 10.1053/j.gastro.2022.07.076 PMC10257389

[31] P. R. Kuninty, R. Bansal, S. W. L. de Geus, D. F. Mardhian, J. Schnittert, J. van Baarlen, G. Storm, M. F. Bijlsma, H. W. van Laarhoven, J. M. Metselaar, P. J. K. Kuppen, A. L. Vahrmeijer, A. Östman, C. F. M. Sier, J. Prakash, ITGA5 inhibition in pancreatic stellate cells attenuates desmoplasia and potentiates efficacy of chemotherapy in pancreatic cancer. Sci. Adv. 5, eaax2770 (2019). 31517053 10.1126/sciadv.aax2770 PMC6726450

[32] J. L. Chitty, M. Yam, L. Perryman, A. L. Parker, J. N. Skhinas, Y. F. I. Setargew, E. T. Y. Mok, E. Tran, R. D. Grant, S. L. Latham, B. A. Pereira, S. C. Ritchie, K. J. Murphy, M. Trpceski, A. D. Findlay, P. Melenec, E. C. Filipe, A. Nadalini, S. Velayuthar, G. Major, K. Wyllie, M. Papanicolaou, S. Ratnaseelan, P. A. Phillips, G. Sharbeen, J. Youkhana, A. Russo, A. Blackwell, J. F. Hastings, M. C. Lucas, C. R. Chambers, D. A. Reed, J. Stoehr, C. Vennin, R. Pidsley, A. Zaratzian, A. M. Da Silva, M. Tayao, B. Charlton, D. Herrmann, M. Nobis, S. J. Clark, A. V. Biankin, A. L. Johns, D. R. Croucher, A. Nagrial, A. J. Gill, S. M. Grimmond; Australian Pancreatic Cancer Genome Initiative (APGI), Australian Pancreatic Cancer Matrix Atlas (APMA), M. Pajic, P. Timpson, W. Jarolimek, T. R. Cox, A first-in-class pan-lysyl oxidase inhibitor impairs stromal remodeling and enhances gemcitabine response and survival in pancreatic cancer. Nat. Cancer 4, 1326–1344 (2023). 37640930 10.1038/s43018-023-00614-y PMC10518255

[33] A. Naba, K. R. Clauser, S. Hoersch, H. Liu, S. A. Carr, R. O. Hynes, The matrisome: In silico definition and in vivo characterization by proteomics of normal and tumor extracellular matrices. Mol. Cell. Proteomics 11, M111.014647 (2012). 10.1074/mcp.M111.014647 PMC3322572 22159717

[34] A. E. Mayorca-Guiliani, C. D. Madsen, T. R. Cox, E. R. Horton, F. A. Venning, J. T. Erler, ISDoT: In situ decellularization of tissues for high-resolution imaging and proteomic analysis of native extracellular matrix. Nat. Med. 23, 890–898 (2017). 28604702 10.1038/nm.4352

[35] A. E. Mayorca-Guiliani, O. Willacy, C. D. Madsen, M. Rafaeva, S. Elisabeth Heumüller, F. Bock, G. Sengle, M. Koch, T. Imhof, F. Zaucke, R. Wagener, T. Sasaki, J. T. Erler, R. Reuten, Decellularization and antibody staining of mouse tissues to map native extracellular matrix structures in 3D. Nat. Protoc. 14, 3395–3425 (2019). 31705125 10.1038/s41596-019-0225-8

[36] S. R. Hingorani, L. Wang, A. S. Multani, C. Combs, T. B. Deramaudt, R. H. Hruban, A. K. Rustgi, S. Chang, D. A. Tuveson, *Trp53R172H* and *KrasG12D* cooperate to promote chromosomal instability and widely metastatic pancreatic ductal adenocarcinoma in mice. Cancer Cell 7, 469–483 (2005). 15894267 10.1016/j.ccr.2005.04.023

[37] J. P. Morton, P. Timpson, S. A. Karim, R. A. Ridgway, D. Athineos, B. Doyle, N. B. Jamieson, K. A. Oien, A. M. Lowy, V. G. Brunton, M. C. Frame, T. R. J. Evans, O. J. Sansom, Mutant p53 drives metastasis and overcomes growth arrest/senescence in pancreatic cancer. Proc. Natl. Acad. Sci. U.S.A. 107, 246–251 (2010). 20018721 10.1073/pnas.0908428107 PMC2806749

[38] E. Kohfeldt, T. Sasaki, W. Göhring, R. Timpl, Nidogen-2: A new basement membrane protein with diverse binding properties. J. Mol. Biol. 282, 99–109 (1998). 9733643 10.1006/jmbi.1998.2004

[39] K. Salmivirta, J. F. Talts, M. Olsson, T. Sasaki, R. Timpl, P. Ekblom, Binding of mouse nidogen-2 to basement membrane components and cells and its expression in embryonic and adult tissues suggest complementary functions of the two nidogens. Exp. Cell Res. 279, 188–201 (2002). 12243745 10.1006/excr.2002.5611

[40] Z. H. Yu, Y. M. Wang, Y. Z. Jiang, S. J. Ma, Q. Zhong, Y. Y. Wan, X. W. Wang, NID2 can serve as a potential prognosis prediction biomarker and promotes the invasion and migration of gastric cancer. Pathol. Res. Pract. 215, 152553 (2019). 31362888 10.1016/j.prp.2019.152553

[41] A. W. Y. Chai, A. K. L. Cheung, W. Dai, J. M. Y. Ko, N. P. Y. Lee, K. T. Chan, S. Y. K. Law, M. L. Lung, Elevated levels of serum nidogen-2 in esophageal squamous cell carcinoma. Cancer Biomark. 21, 583–590 (2018). 29278876 10.3233/CBM-170484 PMC13078288

[42] H. A. Torky, A. Sherif, A. Abo-Louz, M. Ali, A. Ahmed, A. Ali, Evaluation of serum nidogen-2 as a screening and diagnostic tool for ovarian cancer. Gynecol. Obstet. Invest. 83, 461–465 (2018). 29131023 10.1159/000481798

[43] Y. Sha, A. Q. Mao, Y. J. Liu, J. P. Li, Y. T. Gong, D. Xiao, J. Huang, Y. W. Gao, M. Y. Wu, H. Shen, Nidogen-2 (NID2) is a key factor in collagen causing poor response to immunotherapy in melanoma. Pharmgenomics Pers. Med. 16, 153–172 (2023). 36908806 10.2147/PGPM.S399886 PMC9994630

[44] M. Bartoschek, N. Oskolkov, M. Bocci, J. Lövrot, C. Larsson, M. Sommarin, C. D. Madsen, D. Lindgren, G. Pekar, G. Karlsson, M. Ringnér, J. Bergh, Å. Björklund, K. Pietras, Spatially and functionally distinct subclasses of breast cancer-associated fibroblasts revealed by single cell RNA sequencing. Nat. Commun. 9, 5150 (2018). 30514914 10.1038/s41467-018-07582-3 PMC6279758

[45] R. Reuten, S. Zendehroud, M. Nicolau, L. Fleischhauer, A. Laitala, S. Kiderlen, D. Nikodemus, L. Wullkopf, S. R. Nielsen, S. McNeilly, C. Prein, M. Rafaeva, E. M. Schoof, B. Furtwängler, B. T. Porse, H. Kim, K. J. Won, S. Sudhop, K. W. Zornhagen, F. Suhr, E. Maniati, O. M. T. Pearce, M. Koch, L. B. Oddershede, T. van Agtmael, C. D. Madsen, A. E. Mayorca-Guiliani, W. Bloch, R. R. Netz, H. Clausen-Schaumann, J. T. Erler, Basement membrane stiffness determines metastases formation. Nat. Mater. 20, 892–903 (2021). 33495631 10.1038/s41563-020-00894-0

[46] D. Novo, N. Heath, L. Mitchell, G. Caligiuri, A. MacFarlane, D. Reijmer, L. Charlton, J. Knight, M. Calka, E. McGhee, E. Dornier, D. Sumpton, S. Mason, A. Echard, K. Klinkert, J. Secklehner, F. Kruiswijk, K. Vousden, I. R. Macpherson, K. Blyth, P. Bailey, H. Yin, L. M. Carlin, J. Morton, S. Zanivan, J. C. Norman, Mutant p53s generate pro-invasive niches by influencing exosome podocalyxin levels. Nat. Commun. 9, 5069 (2018). 30498210 10.1038/s41467-018-07339-y PMC6265295

[47] M. Papanicolaou, A. L. Parker, M. Yam, E. C. Filipe, S. Z. Wu, J. L. Chitty, K. Wyllie, E. Tran, E. Mok, A. Nadalini, J. N. Skhinas, M. C. Lucas, D. Herrmann, M. Nobis, B. A. Pereira, A. M. K. Law, L. Castillo, K. J. Murphy, A. Zaratzian, J. F. Hastings, D. R. Croucher, E. Lim, B. G. Oliver, F. V. Mora, B. L. Parker, D. Gallego-Ortega, A. Swarbrick, S. O’Toole, P. Timpson, T. R. Cox, Temporal profiling of the breast tumour microenvironment reveals collagen XII as a driver of metastasis. Nat. Commun. 13, 4587 (2022). 35933466 10.1038/s41467-022-32255-7 PMC9357007

[48] A. S. Barrett, O. Maller, M. W. Pickup, V. M. Weaver, K. C. Hansen, Compartment resolved proteomics reveals a dynamic matrisome in a biomechanically driven model of pancreatic ductal adenocarcinoma. J Immunol Regen Med 1, 67–75 (2018). 36908331 10.1016/j.regen.2018.03.002 PMC10003644

[49] C. Tian, K. R. Clauser, D. Öhlund, S. Rickelt, Y. Huang, M. Gupta, D. R. Mani, S. A. Carr, D. A. Tuveson, R. O. Hynes, Proteomic analyses of ECM during pancreatic ductal adenocarcinoma progression reveal different contributions by tumor and stromal cells. Proc. Natl. Acad. Sci. U.S.A. 116, 19609–19618 (2019). 31484774 10.1073/pnas.1908626116 PMC6765243

[50] B. Erdogan, M. Ao, L. M. White, A. L. Means, B. M. Brewer, L. Yang, M. K. Washington, C. Shi, O. E. Franco, A. M. Weaver, S. W. Hayward, D. Li, D. J. Webb, Cancer-associated fibroblasts promote directional cancer cell migration by aligning fibronectin. J. Cell Biol. 216, 3799–3816 (2017). 29021221 10.1083/jcb.201704053 PMC5674895

[51] K. Foley, S. Muth, E. Jaffee, L. Zheng, Hedgehog signaling stimulates Tenascin C to promote invasion of pancreatic ductal adenocarcinoma cells through Annexin A2. Cell Adh. Migr. 11, 514–523 (2017). 28152318 10.1080/19336918.2016.1259057 PMC5810754

[52] B. W. Miller, J. P. Morton, M. Pinese, G. Saturno, N. B. Jamieson, E. McGhee, P. Timpson, J. Leach, L. McGarry, E. Shanks, P. Bailey, D. Chang, K. Oien, S. Karim, A. Au, C. Steele, C. R. Carter, C. McKay, K. Anderson, T. R. J. Evans, R. Marais, C. Springer, A. Biankin, J. T. Erler, O. J. Sansom, Targeting the LOX/hypoxia axis reverses many of the features that make pancreatic cancer deadly: Inhibition of LOX abrogates metastasis and enhances drug efficacy. EMBO Mol. Med. 7, 1063–1076 (2015). 26077591 10.15252/emmm.201404827 PMC4551344

[53] C. Neuzillet, R. Nicolle, J. Raffenne, A. Tijeras-Raballand, A. Brunel, L. Astorgues-Xerri, S. Vacher, F. Arbateraz, M. Fanjul, M. Hilmi, R. Samain, C. Klein, A. Perraud, V. Rebours, M. Mathonnet, I. Bièche, H. Kocher, J. Cros, C. Bousquet, Periostin- and podoplanin-positive cancer-associated fibroblast subtypes cooperate to shape the inflamed tumor microenvironment in aggressive pancreatic adenocarcinoma. J. Pathol. 258, 408–425 (2022). 36102377 10.1002/path.6011 PMC9828775

[54] A. L. Parker, E. Bowman, A. Zingone, B. M. Ryan, W. A. Cooper, M. Kohonen-Corish, C. C. Harris, T. R. Cox, Extracellular matrix profiles determine risk and prognosis of the squamous cell carcinoma subtype of non-small cell lung carcinoma. Genome Med. 14, 126 (2022). 36404344 10.1186/s13073-022-01127-6 PMC9677915

[55] M. B. Buechler, R. N. Pradhan, A. T. Krishnamurty, C. Cox, A. K. Calviello, A. W. Wang, Y. A. Yang, L. Tam, R. Caothien, M. Roose-Girma, Z. Modrusan, J. R. Arron, R. Bourgon, S. Müller, S. J. Turley, Cross-tissue organization of the fibroblast lineage. Nature 593, 575–579 (2021). 33981032 10.1038/s41586-021-03549-5

[56] J. L. Chitty, Y. F. I. Setargew, T. R. Cox, Targeting the lysyl oxidases in tumour desmoplasia. Biochem. Soc. Trans. 47, 1661–1678 (2019). 31754702 10.1042/BST20190098

[57] G. Tang, M. Cho, X. Wang, OncoDB: An interactive online database for analysis of gene expression and viral infection in cancer. Nucleic Acids Res. 50, D1334–d1339 (2022). 34718715 10.1093/nar/gkab970 PMC8728272

[58] R. A. Moffitt, R. Marayati, E. L. Flate, K. E. Volmar, S. G. H. Loeza, K. A. Hoadley, N. U. Rashid, L. A. Williams, S. C. Eaton, A. H. Chung, J. K. Smyla, J. M. Anderson, H. J. Kim, D. J. Bentrem, M. S. Talamonti, C. A. Iacobuzio-Donahue, M. A. Hollingsworth, J. J. Yeh, Virtual microdissection identifies distinct tumor- and stroma-specific subtypes of pancreatic ductal adenocarcinoma. Nat. Genet. 47, 1168–1178 (2015). 26343385 10.1038/ng.3398 PMC4912058

[59] J. Peng, B. F. Sun, C. Y. Chen, J. Y. Zhou, Y. S. Chen, H. Chen, L. Liu, D. Huang, J. Jiang, G. S. Cui, Y. Yang, W. Wang, D. Guo, M. Dai, J. Guo, T. Zhang, Q. Liao, Y. Liu, Y. L. Zhao, D. L. Han, Y. Zhao, Y. G. Yang, W. Wu, Single-cell RNA-seq highlights intra-tumoral heterogeneity and malignant progression in pancreatic ductal adenocarcinoma. Cell Res. 29, 725–738 (2019). 31273297 10.1038/s41422-019-0195-y PMC6796938

[60] S. Z. Wu, D. L. Roden, C. Wang, H. Holliday, K. Harvey, A. S. Cazet, K. J. Murphy, B. Pereira, G. al-Eryani, N. Bartonicek, R. Hou, J. R. Torpy, S. Junankar, C. L. Chan, C. E. Lam, M. N. Hui, L. Gluch, J. Beith, A. Parker, E. Robbins, D. Segara, C. Mak, C. Cooper, S. Warrier, A. Forrest, J. Powell, S. O'Toole, T. R. Cox, P. Timpson, E. Lim, X. S. Liu, A. Swarbrick, Stromal cell diversity associated with immune evasion in human triple-negative breast cancer. EMBO J. 39, e104063 (2020). 32790115 10.15252/embj.2019104063 PMC7527929

[61] J. Franco-Barraza, D. A. Beacham, M. D. Amatangelo, E. Cukierman, Preparation of extracellular matrices produced by cultured and primary fibroblasts. Curr. Protoc. Cell Biol. 71, 10.19.11–10.19.34 (2016). 10.1002/cpcb.2 PMC5058441 27245425

[62] K. J. Murphy, D. A. Reed, C. R. Chambers, J. Zhu, A. Magenau, B. A. Pereira, P. Timpson, D. Herrmann, Cell-derived matrix assays to assess extracellular matrix architecture and track cell movement. Bio. Protoc. 12, e4570 (2022). 10.21769/BioProtoc.4570 PMC9797363 36618089

[63] J. R. W. Conway, S. C. Warren, D. Herrmann, K. J. Murphy, A. S. Cazet, C. Vennin, R. F. Shearer, M. J. Killen, A. Magenau, P. Mélénec, M. Pinese, M. Nobis, A. Zaratzian, A. Boulghourjian, A. M. da Silva, G. del Monte-Nieto, A. S. A. Adam, R. P. Harvey, J. J. Haigh, Y. Wang, D. R. Croucher, O. J. Sansom, M. Pajic, C. E. Caldon, J. P. Morton, P. Timpson, Intravital imaging to monitor therapeutic response in moving hypoxic regions resistant to PI3K pathway targeting in pancreatic cancer. Cell Rep. 23, 3312–3326 (2018). 29898401 10.1016/j.celrep.2018.05.038 PMC6019737

[64] A. Chou, D. Froio, A. M. Nagrial, A. Parkin, K. J. Murphy, V. T. Chin, D. Wohl, A. Steinmann, R. Stark, A. Drury, S. N. Walters, C. Vennin, A. Burgess, M. Pinese, L. A. Chantrill, M. J. Cowley, T. J. Molloy; Australian Pancreatic Cancer Genome Initiative (APGI), N. Waddell, A. Johns, S. M. Grimmond, D. K. Chang, A. V. Biankin, O. J. Sansom, J. P. Morton, S. T. Grey, T. R. Cox, J. Turchini, J. Samra, S. J. Clarke, P. Timpson, A. J. Gill, M. Pajic, Tailored first-line and second-line CDK4-targeting treatment combinations in mouse models of pancreatic cancer. Gut 67, 2142–2155 (2018). 29080858 10.1136/gutjnl-2017-315144 PMC6241608

[65] F. Kai, A. P. Drain, V. M. Weaver, The extracellular matrix modulates the metastatic journey. Dev. Cell 49, 332–346 (2019). 31063753 10.1016/j.devcel.2019.03.026 PMC6527347

[66] R. Malik, P. I. Lelkes, E. Cukierman, Biomechanical and biochemical remodeling of stromal extracellular matrix in cancer. Trends Biotechnol. 33, 230–236 (2015). 25708906 10.1016/j.tibtech.2015.01.004 PMC4380578

[67] T. R. Cox, The matrix in cancer. Nat. Rev. Cancer 21, 217–238 (2021). 33589810 10.1038/s41568-020-00329-7

[68] A. R. Cortazar, J. A. Oguiza, A. M. Aransay, J. L. Lavín, VerSeDa: Vertebrate secretome database. Database 2017, baw171 (2017). 28365718 10.1093/database/baw171 PMC5467544

[69] D. Gallego-Ortega, A. Ledger, D. L. Roden, A. M. K. Law, A. Magenau, Z. Kikhtyak, C. Cho, S. L. Allerdice, H. J. Lee, F. Valdes-Mora, D. Herrmann, R. Salomon, A. I. J. Young, B. Y. Lee, C. M. Sergio, W. Kaplan, C. Piggin, J. R. W. Conway, B. Rabinovich, E. K. A. Millar, S. R. Oakes, T. Chtanova, A. Swarbrick, M. J. Naylor, S. O’Toole, A. R. Green, P. Timpson, J. M. W. Gee, I. O. Ellis, S. J. Clark, C. J. Ormandy, ELF5 drives lung metastasis in luminal breast cancer through recruitment of Gr1+ CD11b+ myeloid-derived suppressor cells. PLOS Biol. 13, e1002330 (2016). 10.1371/journal.pbio.1002330 PMC4696735 26717410

[70] M. Nobis, E. J. McGhee, D. Herrmann, A. Magenau, J. P. Morton, K. I. Anderson, P. Timpson, Monitoring the dynamics of Src activity in response to anti-invasive dasatinib treatment at a subcellular level using dual intravital imaging. Cell Adh. Migr. 8, 478–486 (2014). 25482620 10.4161/19336918.2014.970004 PMC4594577

[71] A. Floerchinger, K. J. Murphy, S. L. Latham, S. C. Warren, A. T. McCulloch, Y. K. Lee, J. Stoehr, P. Mélénec, C. S. Guaman, X. L. Metcalf, V. Lee, A. Zaratzian, A. da Silva, M. Tayao, S. Rolo, M. Phimmachanh, G. Sultani, L. McDonald, S. M. Mason, N. Ferrari, L. M. Ooms, A. K. E. Johnsson, H. J. Spence, M. F. Olson, L. M. Machesky, O. J. Sansom, J. P. Morton, C. A. Mitchell, M. S. Samuel, D. R. Croucher, H. C. E. Welch, K. Blyth, C. E. Caldon, D. Herrmann, K. I. Anderson, P. Timpson, M. Nobis, Optimizing metastatic-cascade-dependent Rac1 targeting in breast cancer: Guidance using optical window intravital FRET imaging. Cell Rep. 36, 109689 (2021). 34525350 10.1016/j.celrep.2021.109689

[72] C. L. G. J. Scheele, D. Herrmann, E. Yamashita, C. Lo Celso, C. N. Jenne, M. H. Oktay, D. Entenberg, P. Friedl, R. Weigert, F. L. B. Meijboom, M. Ishii, P. Timpson, J. van Rheenen, Multiphoton intravital microscopy of rodents. Nat. Rev. Methods Primers 2, 89 (2022). 37621948 10.1038/s43586-022-00168-w PMC10449057

[73] J. R. Bumgarner, R. J. Nelson, Open-source analysis and visualization of segmented vasculature datasets with VesselVio. Cell Rep. Methods 2, 100189 (2022). 35497491 10.1016/j.crmeth.2022.100189 PMC9046271

[74] N. Miosge, T. Sasaki, R. Timpl, Evidence of nidogen-2 compensation for nidogen-1 deficiency in transgenic mice. Matrix Biol. 21, 611–621 (2002). 12475645 10.1016/s0945-053x(02)00070-7

[75] B. L. Bader, N. Smyth, S. Nedbal, N. Miosge, A. Baranowsky, S. Mokkapati, M. Murshed, R. Nischt, Compound genetic ablation of nidogen 1 and 2 causes basement membrane defects and perinatal lethality in mice. Mol. Cell. Biol. 25, 6846–6856 (2005). 16024816 10.1128/MCB.25.15.6846-6856.2005 PMC1190363

[76] C. Mao, Z. Ma, Y. Jia, W. Li, N. Xie, G. Zhao, B. Ma, F. Yu, J. Sun, Y. Zhou, Q. Cui, Y. Fu, W. Kong, Nidogen-2 maintains the contractile phenotype of vascular smooth muscle cells and prevents neointima formation via bridging Jagged1-Notch3 signaling. Circulation 144, 1244–1261 (2021). 34315224 10.1161/CIRCULATIONAHA.120.053361

[77] G. Mucciolo, J. Araos Henríquez, M. Jihad, S. Pinto Teles, J. S. Manansala, W. Li, S. Ashworth, E. G. Lloyd, P. S. W. Cheng, W. Luo, A. Anand, A. Sawle, A. Piskorz, G. Biffi, EGFR-activated myofibroblasts promote metastasis of pancreatic cancer. Cancer Cell 42, 101–118.e11 (2024). 38157863 10.1016/j.ccell.2023.12.002

[78] M. R. Farren, L. Sayegh, M. B. Ware, H. R. Chen, J. Gong, Y. Liang, A. Krasinskas, S. K. Maithel, M. Zaidi, J. M. Sarmiento, D. Kooby, P. Patel, B. el-Rayes, W. Shaib, G. B. Lesinski, Immunologic alterations in the pancreatic cancer microenvironment of patients treated with neoadjuvant chemotherapy and radiotherapy. JCI Insight 5, e130362 (2020). 31830001 10.1172/jci.insight.130362 PMC7030821

[79] K. C. D. Roet, E. H. P. Franssen, F. M. de Bree, A. H. W. Essing, S.-J. J. Zijlstra, N. D. Fagoe, H. M. Eggink, R. Eggers, A. B. Smit, R. E. van Kesteren, J. Verhaagen, A multilevel screening strategy defines a molecular fingerprint of proregenerative olfactory ensheathing cells and identifies SCARB2, a protein that improves regenerative sprouting of injured sensory spinal axons. J. Neurosci. 33, 11116–11135 (2013). 23825416 10.1523/JNEUROSCI.1002-13.2013 PMC6618611

[80] A. V. Pinho, M. van Bulck, L. Chantrill, M. Arshi, T. Sklyarova, D. Herrmann, C. Vennin, D. Gallego-Ortega, A. Mawson, M. Giry-Laterriere, A. Magenau, G. Leuckx, L. Baeyens, A. J. Gill, P. Phillips, P. Timpson, A. V. Biankin, J. Wu, I. Rooman, ROBO2 is a stroma suppressor gene in the pancreas and acts via TGF-β signalling. Nat. Commun. 9, 5083 (2018). 30504844 10.1038/s41467-018-07497-z PMC6269509

[81] K. Koikawa, S. Kibe, F. Suizu, N. Sekino, N. Kim, T. D. Manz, B. J. Pinch, D. Akshinthala, A. Verma, G. Gaglia, Y. Nezu, S. Ke, C. Qiu, K. Ohuchida, Y. Oda, T. H. Lee, B. Wegiel, J. G. Clohessy, N. London, S. Santagata, G. M. Wulf, M. Hidalgo, S. K. Muthuswamy, M. Nakamura, N. S. Gray, X. Z. Zhou, K. P. Lu, Targeting Pin1 renders pancreatic cancer eradicable by synergizing with immunochemotherapy. Cell 184, 4753–4771.e27 (2021). 34388391 10.1016/j.cell.2021.07.020 PMC8557351

[82] T. L. Yeung, C. S. Leung, K. P. Yip, J. Sheng, L. Vien, L. C. Bover, M. J. Birrer, S. T. C. Wong, S. C. Mok, Anticancer immunotherapy by MFAP5 blockade inhibits fibrosis and enhances chemosensitivity in ovarian and pancreatic cancer. Clin. Cancer Res. 25, 6417–6428 (2019). 31332047 10.1158/1078-0432.CCR-19-0187 PMC6825539

[83] Z. Xiao, L. Todd, L. Huang, E. Noguera-Ortega, Z. Lu, L. Huang, M. Kopp, Y. Li, N. Pattada, W. Zhong, W. Guo, J. Scholler, M. Liousia, C. A. Assenmacher, C. H. June, S. M. Albelda, E. Puré, Desmoplastic stroma restricts T cell extravasation and mediates immune exclusion and immunosuppression in solid tumors. Nat. Commun. 14, 5110 (2023). 37607999 10.1038/s41467-023-40850-5 PMC10444764

[84] A. B. Blair, J. Wang, J. Davelaar, A. Baker, K. Li, N. Niu, J. Wang, Y. Shao, V. Funes, P. Li, J. A. Pachter, D. C. Maneval, F. Dezem, J. Plummer, K. S. Chan, J. Gong, A. E. Hendifar, S. J. Pandol, R. Burkhart, Y. Zhang, L. Zheng, A. Osipov, Dual stromal targeting sensitizes pancreatic adenocarcinoma for anti-programmed cell death protein 1 therapy. Gastroenterology 163, 1267–1280.e7 (2022). 35718227 10.1053/j.gastro.2022.06.027 PMC9613523

[85] C. S. Hughes, S. Moggridge, T. Müller, P. H. Sorensen, G. B. Morin, J. Krijgsveld, Single-pot, solid-phase-enhanced sample preparation for proteomics experiments. Nat. Protoc. 14, 68–85 (2019). 30464214 10.1038/s41596-018-0082-x

[86] R. Bruderer, O. M. Bernhardt, T. Gandhi, Y. Xuan, J. Sondermann, M. Schmidt, D. Gomez-Varela, L. Reiter, Optimization of experimental parameters in data-independent mass spectrometry significantly increases depth and reproducibility of results. Mol. Cell. Proteomics 16, 2296–2309 (2017). 29070702 10.1074/mcp.RA117.000314 PMC5724188

[87] I. de Santiago, C. Yau, L. Heij, M. R. Middleton, F. Markowetz, H. I. Grabsch, M. L. Dustin, S. Sivakumar, Immunophenotypes of pancreatic ductal adenocarcinoma: Meta-analysis of transcriptional subtypes. Int. J. Cancer 145, 1125–1137 (2019). 30720864 10.1002/ijc.32186 PMC6767191

