# Supplementary material for: Temporally resolved proteomics identifies nidogen-2 as a cotarget in pancreatic cancer that modulates fibrosis and therapy response
Source: Sci Adv. 2024 Jul 3;10(27):eadl1197. doi: 10.1126/sciadv.adl1197 (PMC11221519; doi:10.1126/sciadv.adl1197)
Supplement: Supplementary file 1 — Figs. S1 to S15 Legends for tables S1 to S7 Legends for movies S1 to S4 Consortium members of the Australian Pancreatic Cancer Genome Initiative (APGI) Consortium members of the Avner Australian Pancreatic Cancer Matrix Atlas (APMA) [file sciadv.adl1197_sm.pdf]

Science Advances
AAAS

Supplementary Materials for

Temporally resolved proteomics identifies nidogen-2 as a cotarget in pancreatic cancer that modulates fibrosis and therapy response

Brooke A. Pereira *et al.*

Corresponding author: Paul Timpson, p.timpson@garvan.org.au; Thomas R. Cox, t.cox@garvan.org.au; Brooke A. Pereira, b.pereira@garvan.org.au

Sci. Adv. **10**, eadl1197 (2024)
DOI: 10.1126/sciadv.adl1197

The PDF file includes:

Figs. S1 to S15
Legends for tables S1 to S7
Legends for movies S1 to S4
Consortium members of the Australian Pancreatic Cancer Genome Initiative (APGI)
Consortium members of the Avner Australian Pancreatic Cancer Matrix Atlas (APMA)

Other Supplementary Material for this manuscript includes the following:

Tables S1 to S7
Movies S1 to S4

Supplementary Figure 1.

A

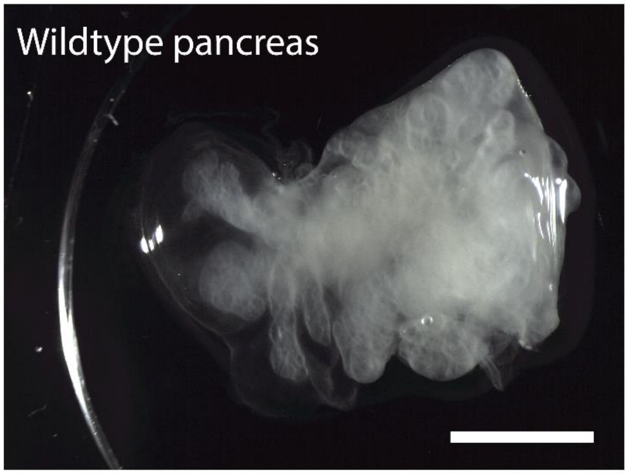

B

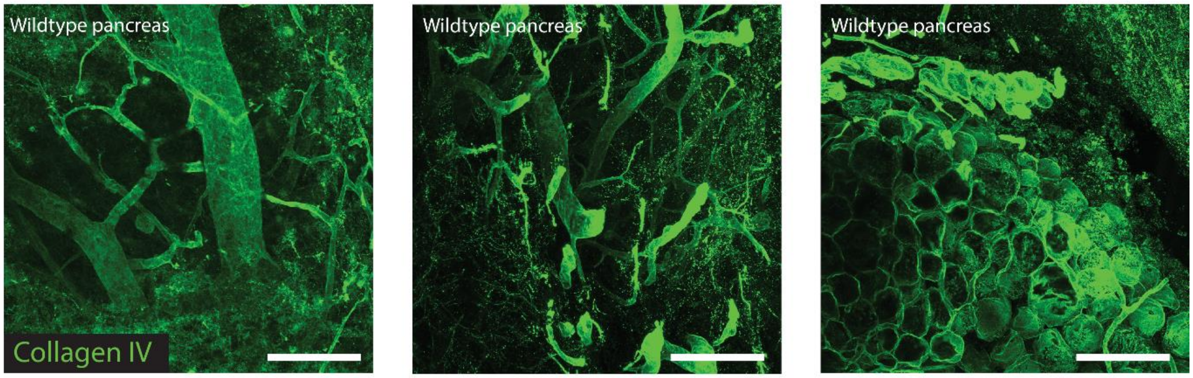

C

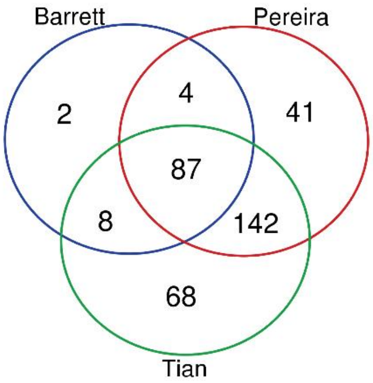

Fig. S1.

A. Representative darkfield image of ISDoT decellularized wildtype pancreas. Scale bar 500 µm. **B.** Representative images of collagen IV immunofluorescence staining (green) of ISDoT decellularized wildtype pancreas. Scale bar 100 µm. **C.** Venn diagram of common and uniquely detected matrisomal pancreatic cancer proteins across the Barrett *et al*. (2018), Tian *et al.* (2019) and Pereira *et al.* dataset.

Supplementary Figure 2.

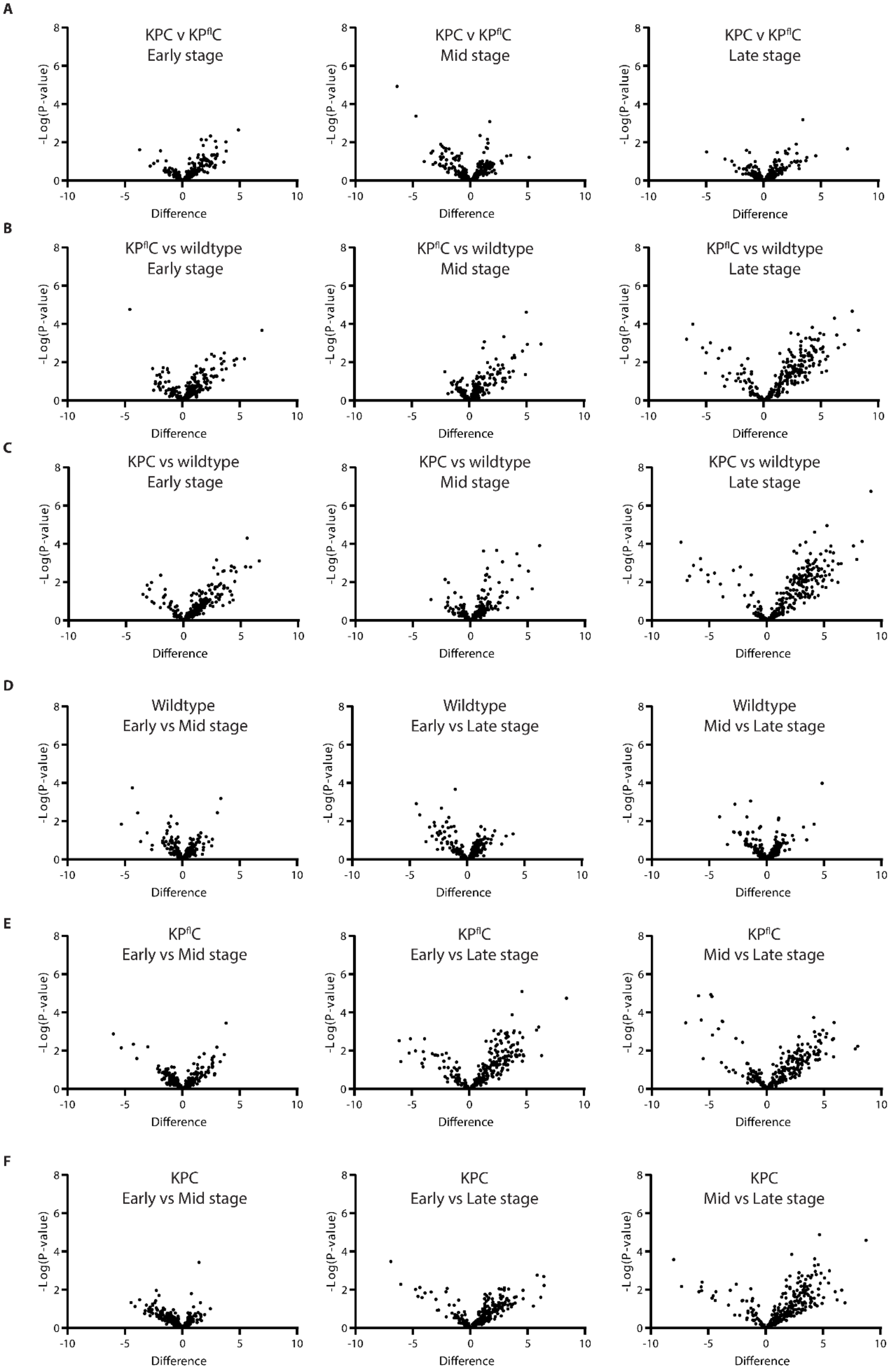

Fig. S2.

A. Volcano plots for matrisomal proteins compared between KPC and $KP^{fl}C$ tumors at early- (left), mid- (center) and late-stage (right). **B.** Volcano plots for matrisomal proteins compared between $KP^{fl}C$ tumors and wildtype pancreas at early- (left), mid- (center) and late-stage (right). **C.** Volcano plots for matrisomal proteins compared between KPC tumors and wildtype pancreas at early- (left), mid- (center) and late-stage (right). **D.** Volcano plots for matrisomal proteins compared between wildtype pancreas at early- *versus* mid-stage (left), early- *versus* late-stage (center) and mid- *versus* late-stage (right). **E.** Volcano plots for matrisomal proteins compared between $KP^{fl}C$ tumors at early- *versus* mid-stage (left), early- *versus* late-stage (center) and mid- *versus* late-stage (right). **F.** Volcano plots for matrisomal proteins compared between KPC tumors at early- *versus* mid-stage (left), early- *versus* late-stage (center) and mid- *versus* late-stage (right).

Supplementary Figure 3.

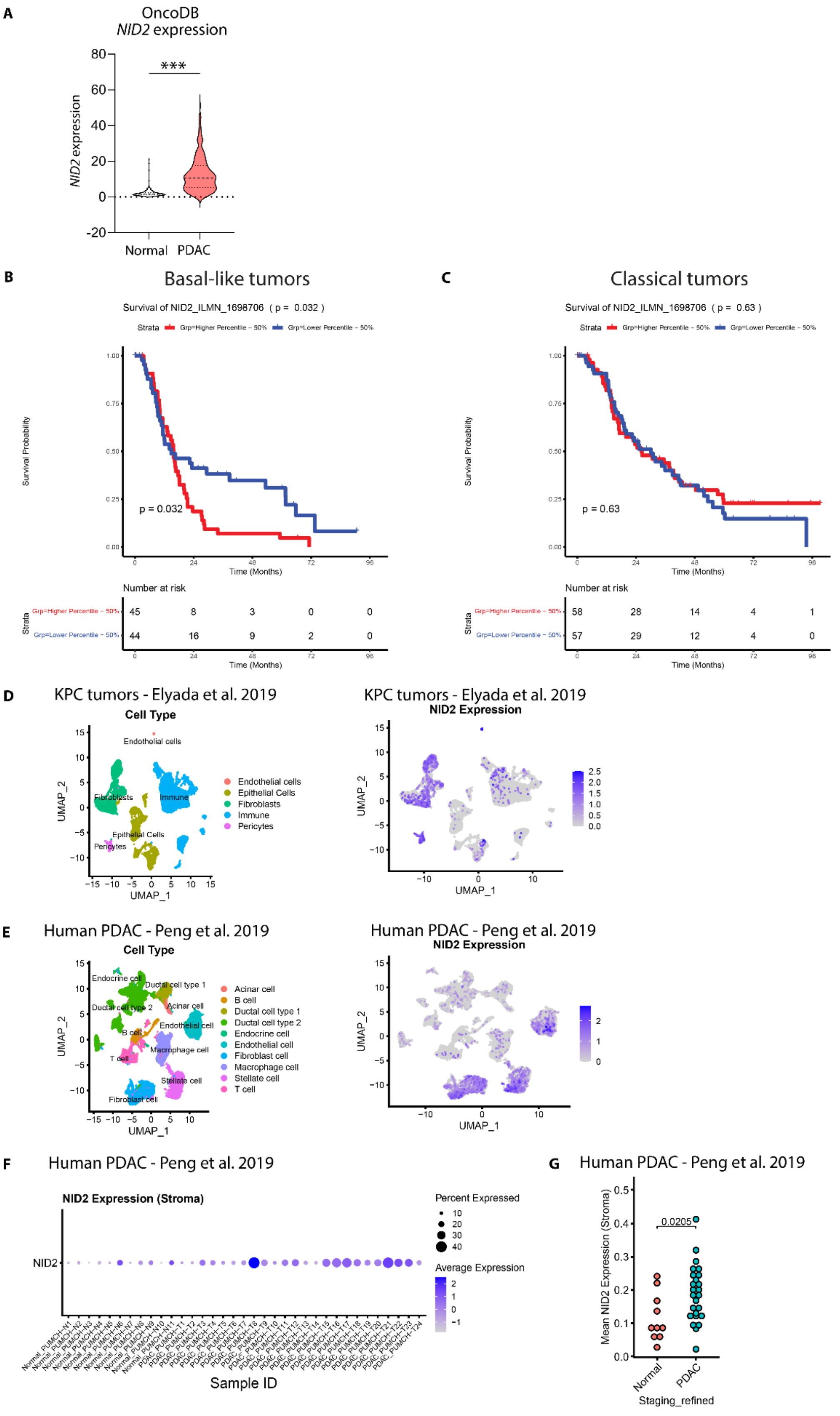

Fig. S3.

A. *NID2* mRNA expression in PDAC tumors (red) compared to normal pancreas (white) from the OncoDB database. Welch's t-test. *** $P < 0.001$. Data represented as a violin plot (minimum, first quartile, median, third quartile, and maximum). **B.** Kaplan-Meier analysis of human PDAC disease survival based on *NID2* mRNA expression, with high *NID2* expression (red; n = 45) *versus* low *NID2* expression (blue; n = 44) in the ICGC cohort for patients that have been further categorized into a basal-like subtype (n = 89). Significance was compared with a log-rank test. P = 0.032. **C.** Kaplan-Meier analysis of human PDAC disease survival based on *NID2* mRNA expression, with high *NID2* expression (red; n = 58) *versus* low *NID2* expression (blue; n = 57) in the ICGC cohort for patients that have been further categorized into a classical subtype (n = 115). Significance was compared with a log-rank test. P = 0.63. **D.** UMAP visualization and clustering of cells analyzed by single-cell RNA-sequencing from Elyada *et al.* (2019) of KPC tumors. Feature plot showing the expression of *NID2* over the UMAP structure and clusters. **E.** UMAP visualization and clustering of cells analyzed by single-cell RNA-Sequencing from Peng *et al.* (2019) of human PDAC specimens. Feature plot showing the expression of *NID2* over the UMAP structure and clusters. **F.** *NID2* mRNA expression across the stromal compartment of each sample in the Peng *et al.* dataset (11 normal pancreas and 24 PDAC). **G.** *NID2* mRNA expression for the stromal compartment of each sample in the Peng *et al.* dataset (11 normal pancreas and 24 PDAC) compared by a student's t-test. P value = 0.0205.

Supplementary Figure 4.

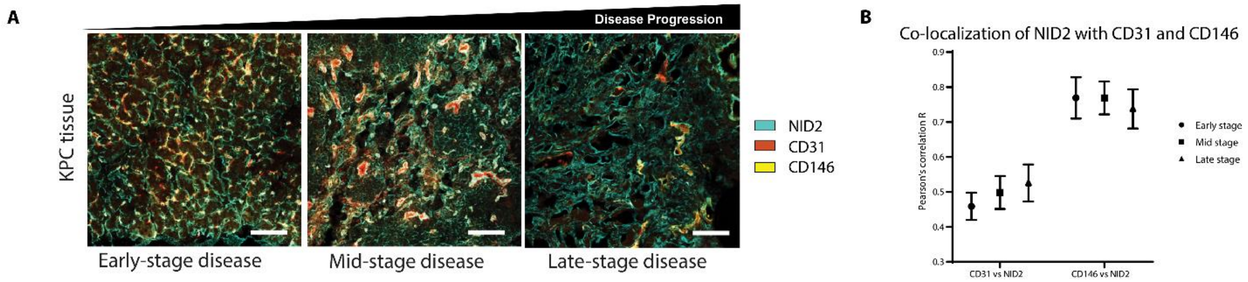

Fig S4.

A. Representative images of KPC tissue at early-, mid- and late-stage disease stained for NID2 (cyan), CD31 (red) and CD146 (yellow) via immunofluorescence. Scale bar 100 μm. **B.** Quantification of co-localization of NID2 *versus* CD31 and NID2 *versus* CD146 at early-, mid- and late-stage disease.

Supplementary Figure 5.

A

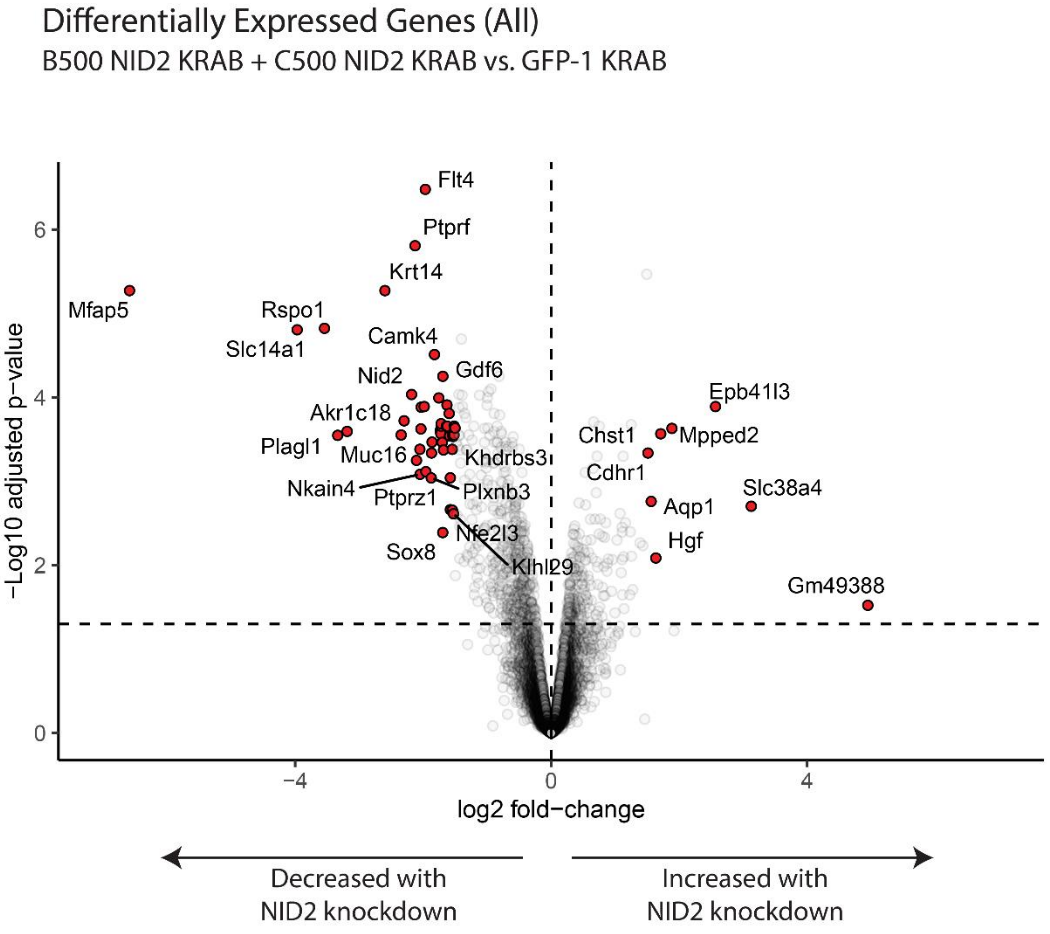

Fig S5.
A. Volcano plot of all differentially expressed genes for B500 NID2 KRAB + C500 NID2 KRAB *versus* GFP-1 KRAB CAF, assessed by RNAseq (adjusted p-value < 0.05, log2 fold change > 1.5). n = 3 biological repeats for each CAF line. P-values adjusted using Benjamini-Hochberg procedure.

Supplementary Figure 6.

A K-means clustering of matrisomal-associated genes

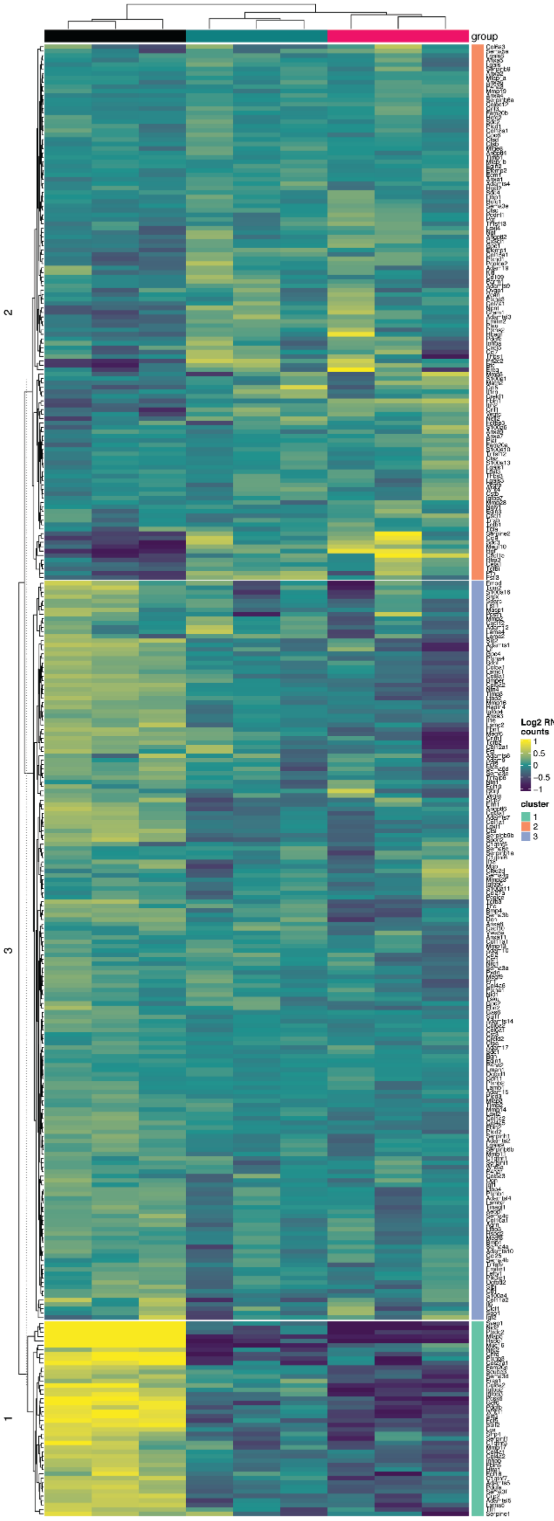

B Matrisomal-associated differentially expressed genes

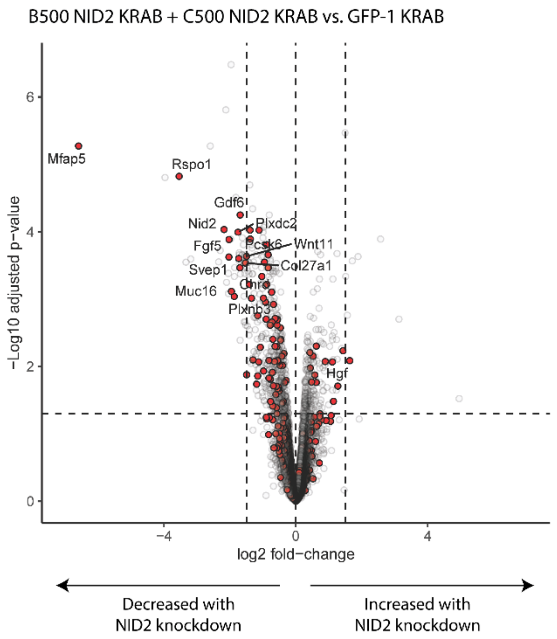

C K-means clustering of basement membrane-associated genes

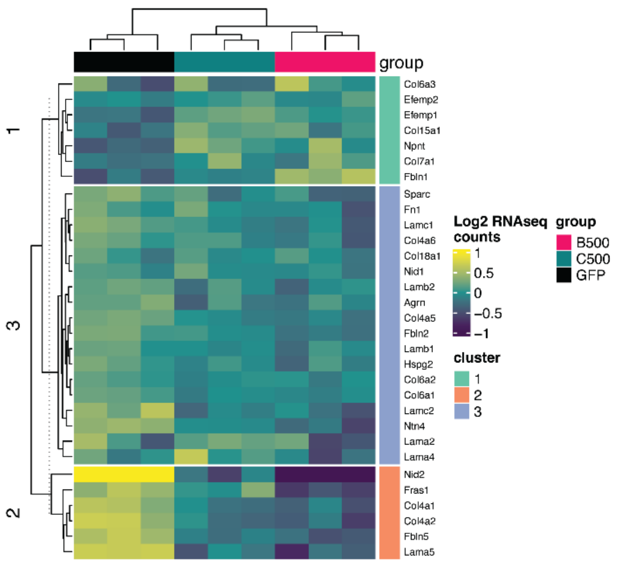

Fig S6.

A. Heatmap representing RNAseq analysis of matrisomal genes for B500 NID2 KRAB (red), C500 NID2 KRAB (green) *versus* GFP-1 KRAB (black) CAFs. Groups identified using k-means clustering analysis of log2 RNAseq counts. **B.** Volcano plot of differentially expressed matrisomal genes (adjusted p-value < 0.05, log2 fold change > 1.5). **C.** Heatmap representing RNAseq analysis of basement membrane genes for B500 NID2 KRAB (red), C500 NID2 KRAB (green) *versus* GFP-1 KRAB (black) CAFs. Groups identified using k-means clustering analysis of log2 RNAseq counts. n = 3 independent biological repeats per CAF line.

Supplementary Figure 7.

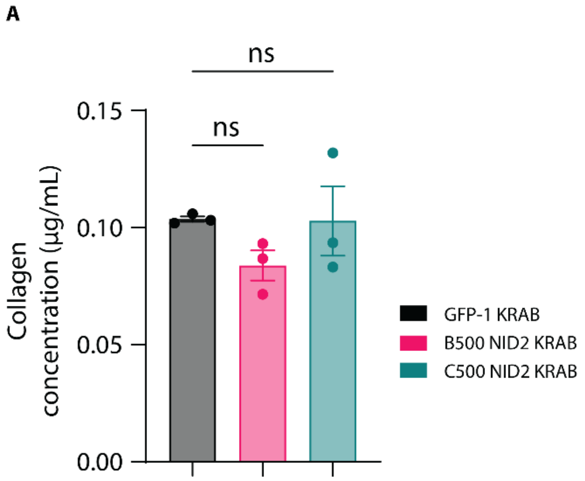

Fig S7.
A. Soluble collagen concentration (µg/mL) extracted from cell-derived matrices (CDMs) at Day 7, deposited by GFP-1 KRAB, B500 NID2 KRAB and C500 NID2 KRAB CAFs. One-way ANOVA with Dunnett's test. ns > 0.05, n = 3 experimental repeats for each line. **B.** *COL1A1* mRNA expression in GFP-1 KRAB, B500 NID2 KRAB, and C500 NID2 KRAB CAFs, assessed via RT-qPCR, relative to *GAPDH* mRNA expression. Kruskal-Wallis test with Dunn's test. ns > 0.05, n = 3 experimental repeats for each line. All data represented as Mean ± SEM.

Supplementary Figure 8.

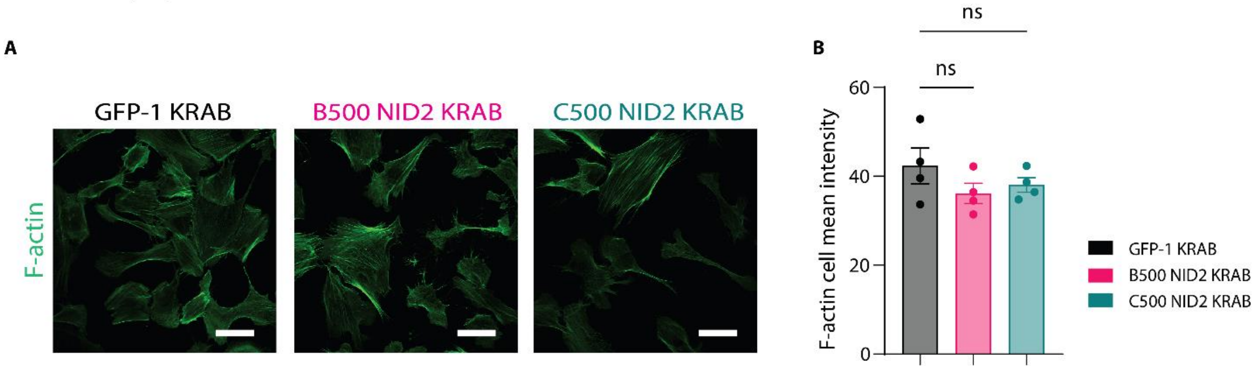

Fig S8.

A. Representative regions of interest (ROIs) of GFP-1 KRAB CAF, B500 NID2 KRAB CAF and C500 NID2 KRAB CAF stained for F-actin. The CAFs stained for F-actin, pMLC2 and DAPI were used for this analysis. Scale bar 50 µm. **B.** Quantification of F-actin cell mean intensity. One-way ANOVA with Dunnett's test. ns > 0.05. n = 3 experimental repeats for each CAF line. All data represented as Mean ± SEM.

Supplementary Figure 9.

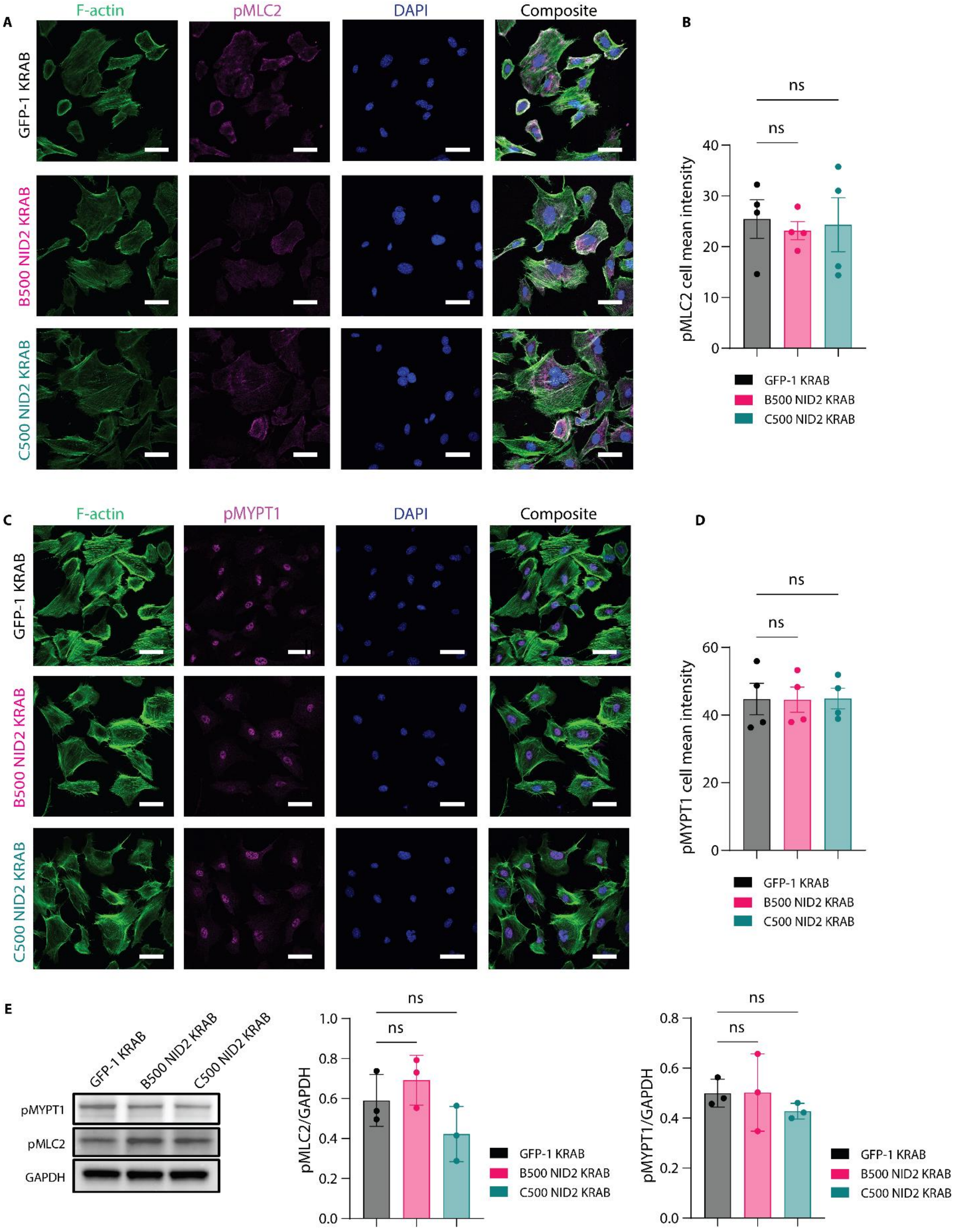

Fig S9.
A. Representative regions of interest (ROIs) of GFP-1 KRAB, B500 NID2 KRAB and C500 NID2 KRAB CAFs stained for F-actin, pMLC2 and DAPI. Scale bar 50 µm. **B.** Quantification of pMLC2 cell mean intensity. One-way ANOVA with Dunnett's. ns > 0.05. n = 3 experimental repeats for each line. **C.** Representative ROIs of GFP-1 KRAB, B500 NID2 KRAB and C500 NID2 KRAB CAFs stained for F-actin, pMYPT1 and DAPI. Scale bar 50 µm. **D.** Quantification of pMYPT1 cell mean intensity. One-way ANOVA with Dunnett's. ns > 0.05. n = 3 experimental repeats for each line. **E.** pMYPT1 and pMLC2 protein expression in GFP-1 KRAB, B500 NID2 KRAB and C500 NID2 KRAB CAFs assessed via western blotting. Quantification of pMLC2 protein expression. Kruskal-Wallis test with Dunn's test (densitometry normalized to GAPDH loading control). ns > 0.05. n = 3 experimental repeats for each line. Quantification of pMYPT1 protein expression. Kruskal-Wallis test with Dunn's test (densitometry normalized to GAPDH loading control). ns > 0.05. n = 3 experimental repeats for each line. All data represented as Mean ± SEM.

Supplementary Figure 10.

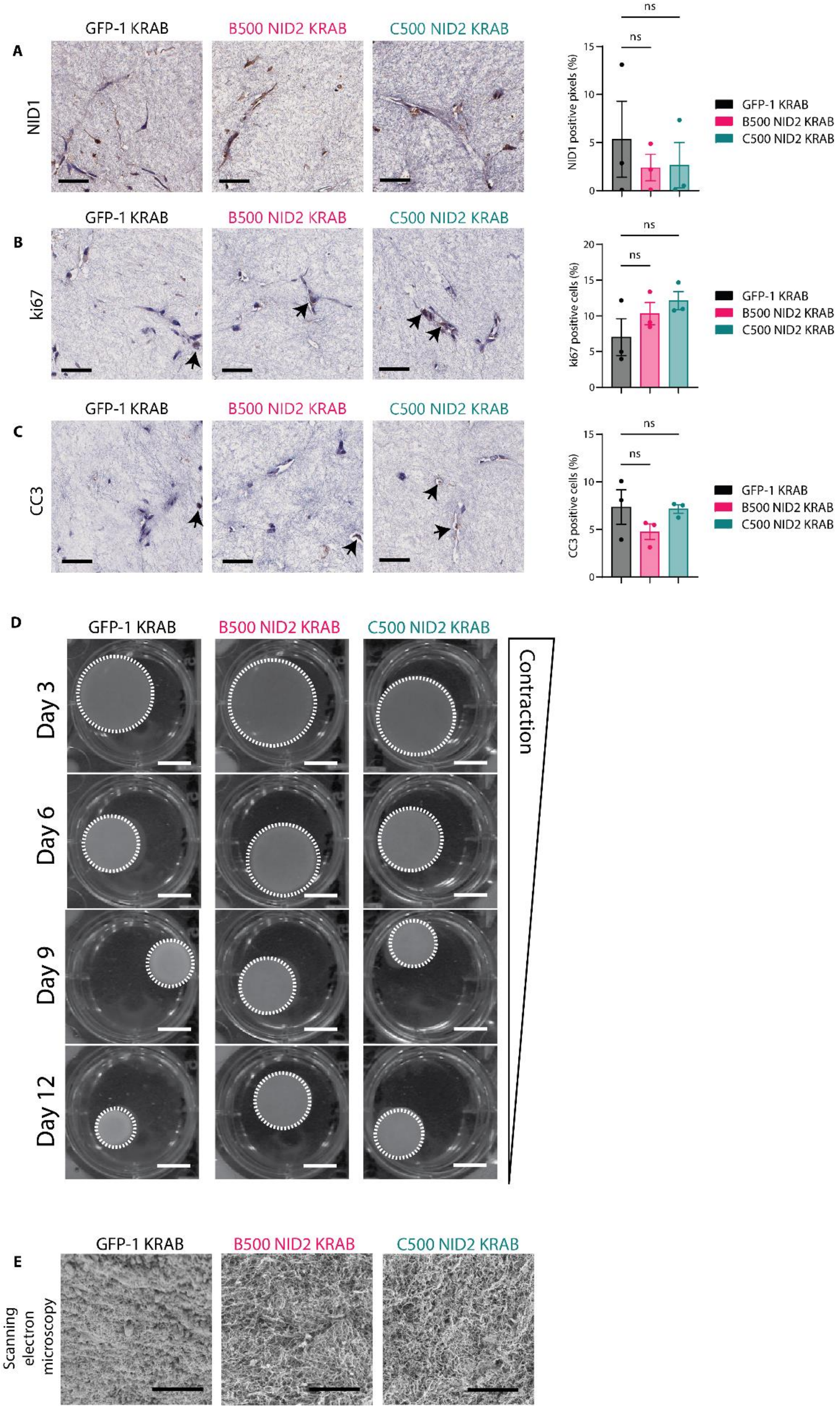

Fig. S10.
A. Representative images of NID1 stained CAF-contracted matrices. Scale bar 50 μm. Quantification of NID1 positive pixels (%) in organotypic matrices. One-way ANOVA with Dunnett's test. ns $P > 0.05$. $n = 3$ experimental repeats in triplicate. **B.** Representative images of ki67 stained CAF-contracted matrices. Arrows indicate positive cells. Scale bar 50 μm. Quantification of ki67 positive cells (%) in organotypic matrices. One-way ANOVA with Dunnett's test. ns $P > 0.05$. $n = 3$ experimental repeats in triplicate. **C.** Representative images of cleaved caspase 3 (CC3) stained CAF-contracted matrices. Arrows indicate positive cells. Scale bar 50 μm. Quantification of CC3 positive cells (%) in organotypic matrices. One-way ANOVA with Dunnett's test. ns $P > 0.05$. $n = 3$ experimental repeats in triplicate. **D.** Representative images of GFP-1 KRAB CAF, B500 NID2 KRAB CAF and C500 NID2 KRAB CAF organotypic matrix contraction size at Day 3, Day 6, Day 9, and Day 12. Scale bar 1 cm. **E.** Scanning electron micrographs (SEM) of GFP-1 KRAB CAF, B500 NID2 KRAB CAF and C500 NID2 KRAB CAF organotypic matrices on Day 12. Scale bar 20 μm.
All data represented as Mean ± SEM.

Supplementary Figure 11.

A

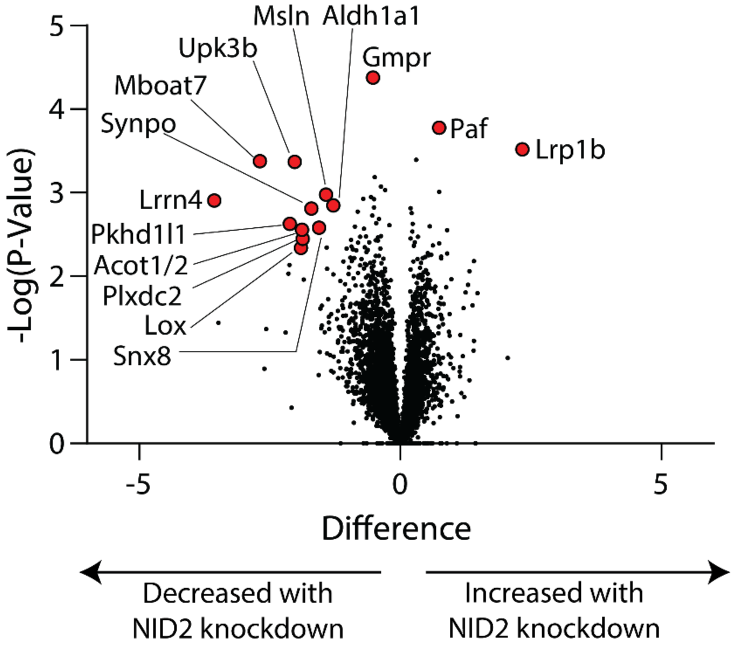

Fig S11.

A. Volcano plot of differentially abundant proteins comparing B500 NID2 KRAB + C500 NID2 KRAB *versus* GFP-1 KRAB CAF lines. Two-sample t test with FDR = 0.05. Y-axis = -log(P-value) and x-axis = difference (fold change). Data derived from n = 3 biological repeats per cell line.

Supplementary Figure 12.

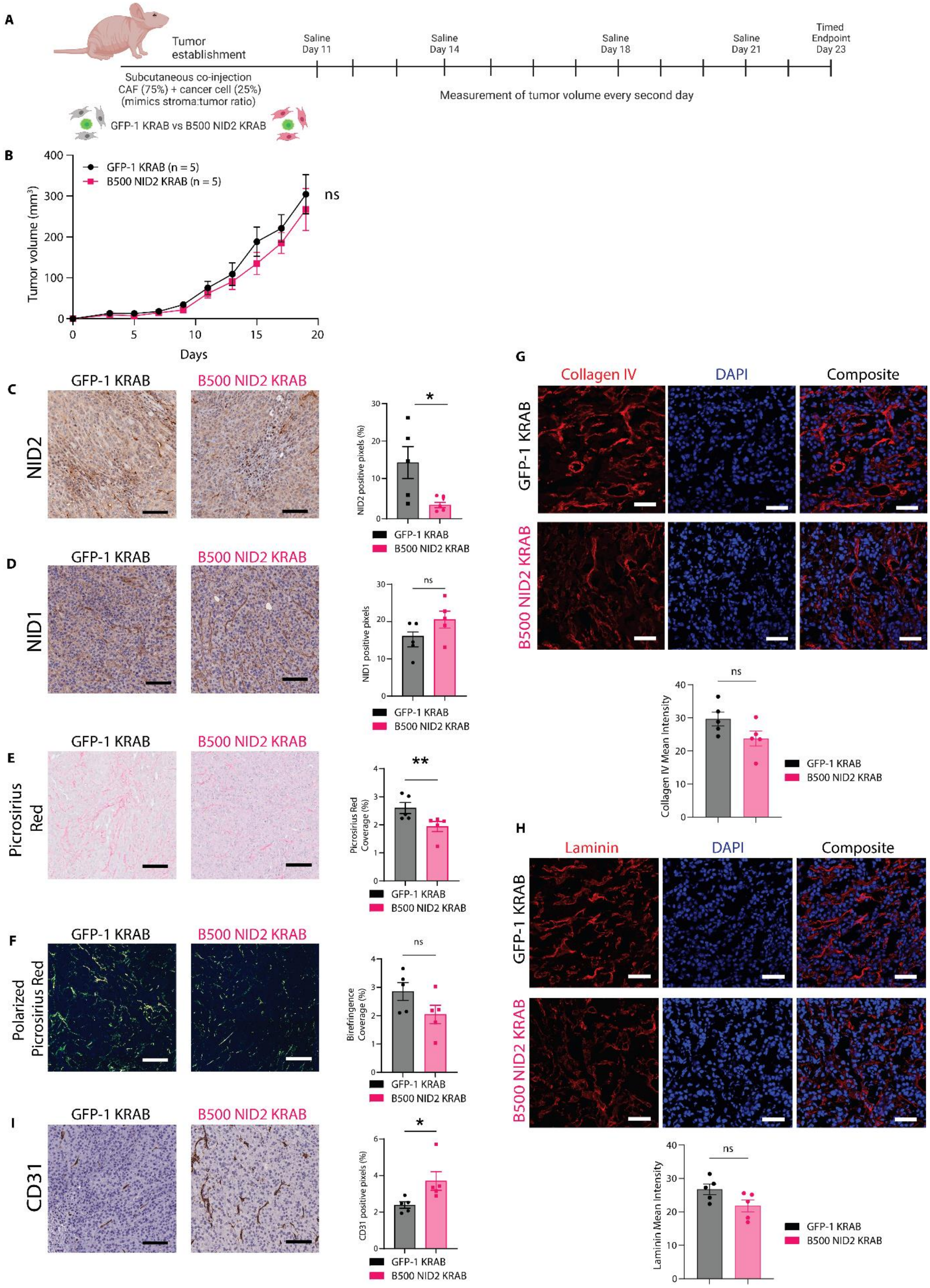

Fig. S12.

A. Timeline for subcutaneous co-injection experiment using GFP-1 KRAB or B500 NID2 KRAB CAFs (75%) with cancer cells (25%). Mice were treated twice weekly with vehicle (saline) beginning Day 11. **B.** Quantification of GFP-1 KRAB CAF (black) and B500 NID2 KRAB CAF (pink) tumor growth over time. Welch's t-test on Day 23. ns $P > 0.05$. n = 5 GFP-1 KRAB CAF mice and n = 5 B500 NID2 KRAB CAF mice. **C.** Representative regions of interest (ROIs) of GFP-1 KRAB CAF and B500 NID2 KRAB CAF tumor sections stained for NID2. Scale bar 100 µm. Quantification of NID2 positive pixels (%). Welch's t-test. * $P < 0.05$. **D.** Representative ROIs of GFP-1 KRAB CAF and B500 NID2 KRAB CAF tumor sections stained for NID1. Scale bar 100 µm. Quantification of NID1 positive pixels (%). Welch's t-test. ns $P > 0.05$. **E.** Representative ROIs of GFP-1 KRAB CAF and B500 NID2 KRAB CAF tumor sections stained with Picrosirius Red. Scale bar 100 µm. Quantification of Picrosirius Red coverage (%). Welch's t-test. ** $P < 0.01$. **F.** Representative ROIs of GFP-1 KRAB CAF and B500 NID2 KRAB CAF tumor sections stained with Picrosirius Red and imaged using polarized light. Scale bar 100 µm. Quantification of birefringence coverage (%). Welch's t-test. ns $P > 0.05$. **G.** Representative ROIs of GFP-1 KRAB CAF and B500 NID2 KRAB CAF tumor sections stained for collagen IV and DAPI. Scale bar 50 µm. Quantification of collagen IV mean intensity. Welch's t-test. ns > 0.05. **H.** Representative ROIs of GFP-1 KRAB CAF and B500 NID2 KRAB CAF tumor sections stained for laminin and DAPI. Scale bar 50 µm. Quantification of laminin mean intensity. Welch's t-test. ns > 0.05. **I.** Representative ROIs of GFP-1 KRAB CAF and B500 NID2 KRAB CAF tumor sections stained for CD31. Scale bar 100 µm. Quantification of CD31 positive pixels (%). Welch's t-test. * $P < 0.05$. All data represented as Mean ± SEM. Schematics created with Biorender.com.

‘

Supplementary Figure 13.

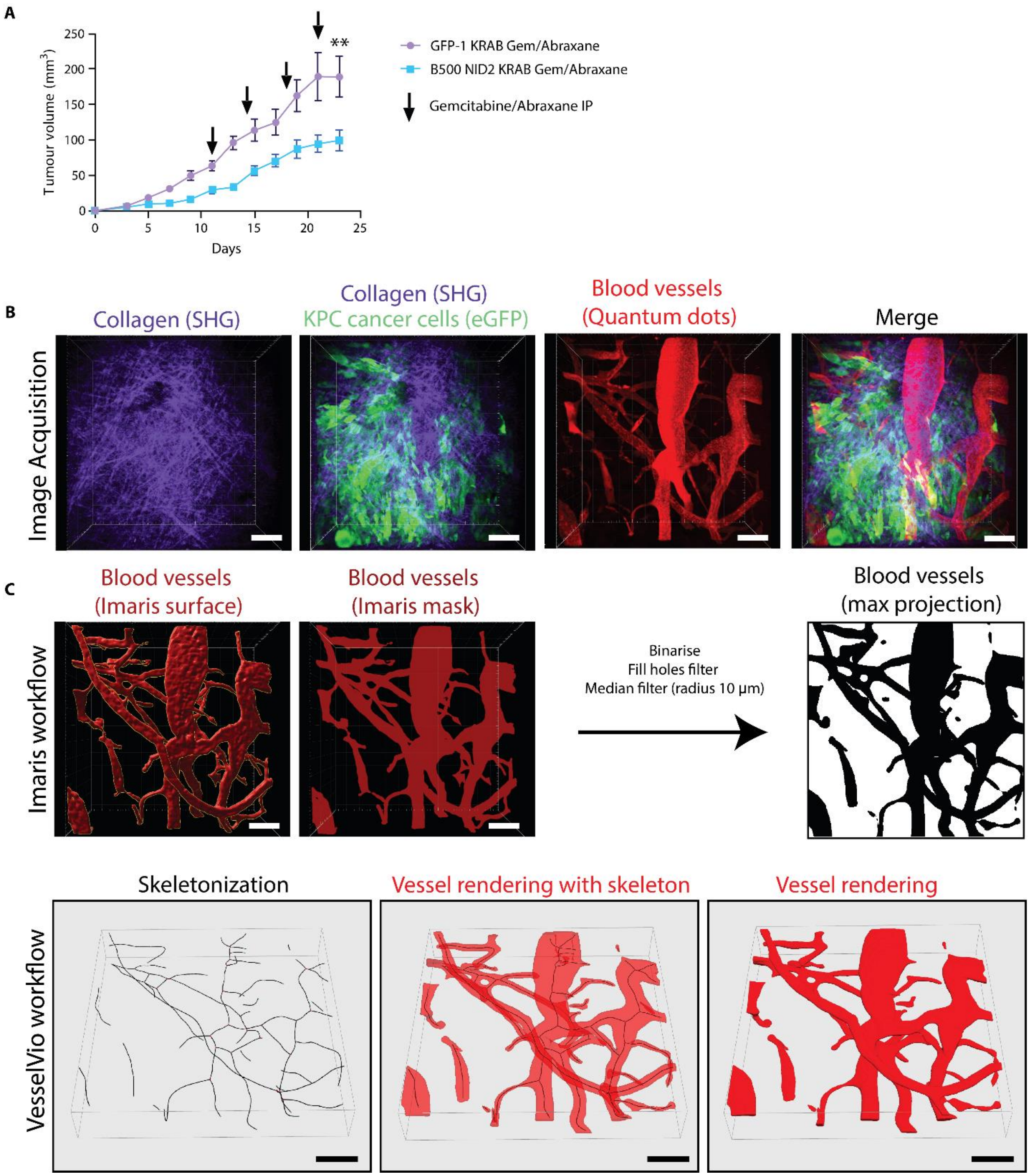

Fig. S13.
A. Quantification of GFP-1 KRAB CAF (purple) and B500 NID2 KRAB CAF (blue) tumor growth over time upon treatment with gemcitabine/Abraxane. Mann-Whitney test on Day 23. ** $P < 0.01$. n = 7 GFP-1 KRAB CAF mice and n = 8 B500 NID2 KRAB CAF mice. **B.** Representative images for B500 NID2 KRAB CAF live tumor image imaging via multiphoton intravital microscopy. Second harmonic generation (SHG) of fibrillar collagens (purple; first panel), fibrillar collagen and eGFP cancer cells (purple and green; second panel), vasculature (Quantum dots, red; third panel) and merged image (fourth panel). Scale bar 100 μm. **C.** Workflow for analyzing the vessels of live subcutaneous tumors using Imaris and VesselVio. A 3D Imaris surface object was generated from the Quantum dots signal (red, first panel). From here, a 3D mask of the Imaris surface was generated (red, second panel). 3D masks were then subjected to a binarize, fill holes and median filter on ImageJ, resulting in a 3D binary z-stack of the vessel structure (black and white; third panel). Binary z-stacks were then analyzed using VesselVio. Vessel skeletonization (black skeleton; first panel), skeletonization with vessel rendering (black skeleton with red rendering; second panel) and vessel rendering alone (red rendering; third panel). Scale bar 100 μm.

Supplementary Figure 14.

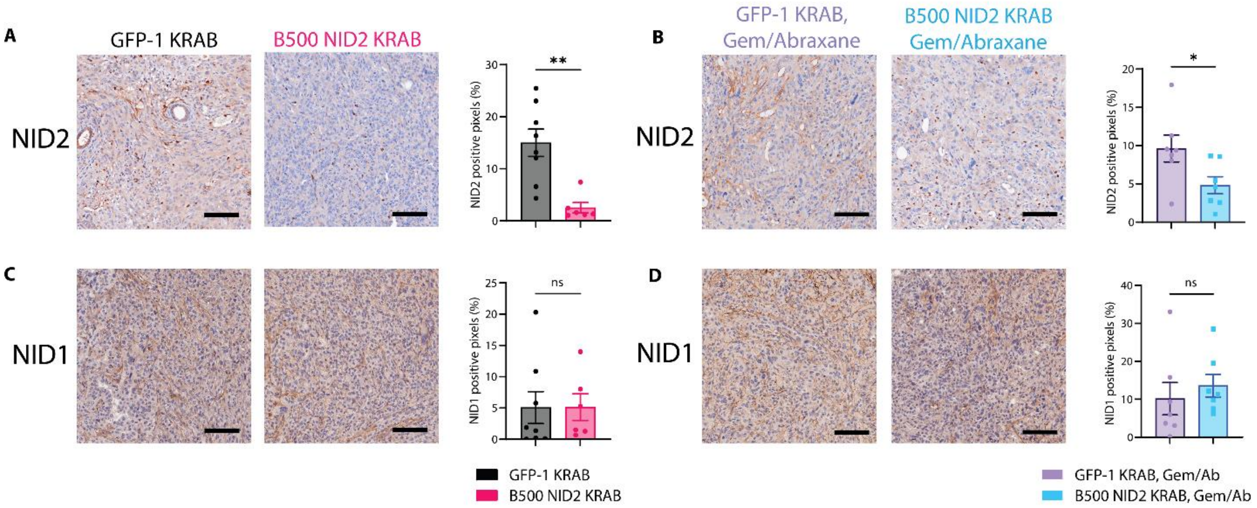

Fig. S14.
A. Representative regions of interest (ROIs) of GFP-1 KRAB CAF (black) and B500 NID2 KRAB CAF (pink) vehicle tumor sections stained for NID2. Scale bar 100 µm. Quantification of NID2 positive pixels (%). Welch's t-test. ** $P < 0.01$. **B.** Representative ROIs of GFP-1 KRAB CAF (purple) and B500 NID2 KRAB CAF (blue) gemcitabine/Abraxane tumor sections stained for NID2. Scale bar 100 µm. Quantification of NID2 positive pixels (%). Mann-Whitney test. * $P < 0.05$. **C.** Representative ROIs of GFP-1 KRAB CAF (black) and B500 NID2 KRAB CAF (pink) vehicle tumor sections stained for NID1. Scale bar 100 µm Quantification of NID1 positive pixels (%). Welch's t-test. ns $P > 0.05$. **D.** Representative ROIs of GFP-1 KRAB CAF (purple) and B500 NID2 KRAB CAF (blue) gemcitabine/Abraxane tumor sections stained for NID1. Scale bar 100 µm. Quantification of NID1 positive pixels (%). Mann-Whitney test. ns $P > 0.05$. All data represented as Mean ± SEM.

Supplementary Figure 15.

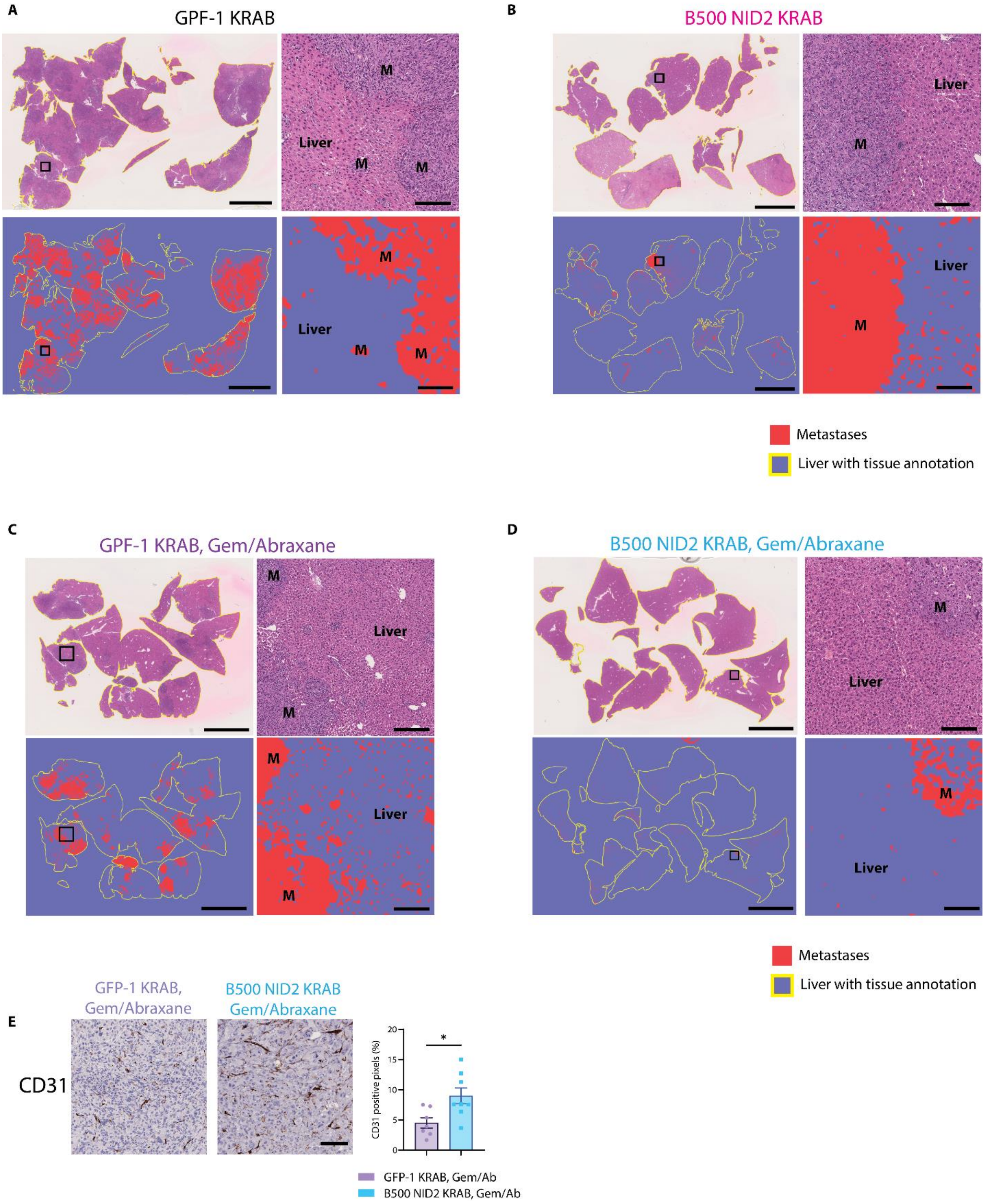

Fig. S15.
A. Representative images of H&E stained liver sections from GFP-1 KRAB CAF vehicle mouse with zoomed region of interest (ROI; black square) (top panel). Qupath metastases detection for GFP-1 CAF vehicle tumors, with zoomed ROI (black square) (bottom panel). Metastases (red, labeled “M”) with liver tissue annotation (yellow outline, labeled “liver”). Scale bar 2 cm for whole tissue section. Scale bar 200 µm for zoomed ROI. **B.** Representative images of H&E stained liver sections from B500 NID2 KRAB CAF vehicle mouse with zoomed ROI (black square) (top panel). Qupath metastases detection for B500 NID2 CAF vehicle tumors, with zoomed ROI (black square) (bottom panel). Metastases (red, labeled “M”) with liver tissue annotation (yellow outline, labeled “liver”). Scale bar 2 cm for whole tissue section. Scale bar 200 µm for zoomed ROI. **C.** Representative images of H&E stained liver sections from GFP-1 KRAB CAF gemcitabine/Abraxane mouse with zoomed ROI black square) (top panel). Qupath metastases detection for GFP-1 CAF gemcitabine/Abraxane tumors, with zoomed ROI (black square) (bottom panel). Metastases (red, labeled “M”) with liver tissue annotation (yellow outline, labeled “liver”). Scale bar 2 cm for whole tissue section. Scale bar 200 µm for zoomed ROI. D. Representative images of H&E stained liver sections from B500 NID2 KRAB CAF gemcitabine/Abraxane mouse with zoomed ROI (black square) (top panel). Qupath metastases detection for B500 NID2 CAF gemcitabine/Abraxane tumors, with zoomed ROI (black square) (bottom panel). Metastases (red, labeled “M”) with liver tissue annotation (yellow outline, labeled “liver”). Scale bar 2 cm for whole tissue section. Scale bar 200 µm for zoomed ROI. **E.** Representative ROIs of GFP-1 KRAB CAF (purple) and B500 NID2 KRAB CAF (blue) gemcitabine/Abraxane tumor sections stained for CD31. Scale bar 100 µm. Quantification of CD31 positive pixels (%). Welch’s t-test. * $P < 0.05$. All data represented as Mean ± SEM.

Table S1.

Mouse genotypes and ages used in mass spectrometry proteomics dataset.

Table S2.

Matrisomal proteins detected in the mass spectrometry proteomics dataset with their matrisomal categories and comparison to the Barrett *et al.* (2018) and Tian *et al.* (2019) datasets.

Table S3.

Differentially abundant proteins detected in the mass spectrometry dataset, compared with a two-sample t-test volcano plot (FDR of 0.05 and a S0 value of 0.1).

Table S4.

Survival data based on *NID2* mRNA expression in PDAC patients within the ICGC cohort according to their molecular subtype classification (basal-like or classical).

Table S5.

All differentially expressed genes determined by RNAseq in B500 NID2 KRAB + C500 NID2 KRAB *versus* GFP-1 KRAB CAFs. Adjusted p-value < 0.05, log2 fold change > 1.5.

Table S6.

Differential gene expression and log CPM of B500 NID2 KRAB + C500 NID2 KRAB *versus* GFP-1 KRAB CAFs for matrisomal and basement membrane genes.

Table S7.

Differentially abundant proteins detected by mass spectrometry proteomic analysis of B500 NID2 KRAB + C500 NID2 KRAB *versus* GFP-1 KRAB CAFs, compared with a two-sample t-test volcano plot (FDR of 0.05 and a S0 value of 0.1).

Movie S1.
Z-stack of ISDoT decellularized wildtype pancreas stained with Collagen IV antibody (green) with maximum projection at the end. Scale bar 100 µm.

Movie S2.
Time-lapse video of a representative GFP-1 KRAB CAF subcutaneous tumor under gemcitabine/Abraxane treatment at Day 23 with fibrillar collagens shown via SHG (magenta), eGFP-tagged KPC cancer cells (green) and Quantum dots flowing through blood vessels (red). Imaged with a 1 second 279 millisecond frame length. Scale bar 100 µm.

Movie S3.
Time-lapse video of a representative B500 NID2 KRAB CAF subcutaneous tumor under gemcitabine/Abraxane treatment at Day 23 with fibrillar collagens shown via SHG (magenta), eGFP-tagged KPC cancer cells (green) and Quantum dots flowing through blood vessels (red). Imaged with a 1 second 279 millisecond frame length. Scale bar 100 µm.

Movie S4.
3D reconstruction of a representative region of interest (ROI) from a B500 NID2 KRAB CAF subcutaneous tumor under gemcitabine/Abraxane treatment at Day 23 with fibrillar collagens shown via SHG (magenta), eGFP-tagged KPC cancer cells (green) and blood vessels shown based on Quantum dot signal (red). Scale bar 70 µm.

Australian Pancreatic Cancer Genome Initiative (APGI) consortium members:

Garvan Institute of Medical Research Amber L. Johns[1], Anthony J. Gill[1,5], Lorraine A. Chantrill[1,22], Paul Timpson[1,] Angela Chou[1,5], Marina Pajic[1], Tanya Dwarte[1], David Herrmann[1], Claire Vennin[1], Thomas R. Cox[1], Brooke A. Pereira[1], Shona Ritchie[1], Daniel A. Reed[1], Cecilia R. Chambers[1], Max Nobis[1], Gloria Jeong[1], Ruth J. Lyons[1], Nicola Blackburn[1], Adnan Nagrial[1], Sean Porazinski[1], Diego Chacon Fajardo[1], Alice Russo[1]. **QIMR Berghofer Medical Research Institute** Nicola Waddell[2], John V. Pearson[2], Katia Nones[2], Felicity Newell[2], Venkateswar Addala[2], Oliver Holmes[2], Conrad Leonard[2], Scott Wood[2]. **University of Melbourne, Centre for Cancer Research** Sean M. Grimmond[3], Oliver Hofmann[3]. **Royal North Shore Hospital** Jaswinder S. Samra[5], Nick Pavlakis[5,] Jennifer Arena[5], Hilda A. High[5], Anubhav Mittal[5]. **Bankstown Hospital** Ray Asghari[6], Neil D. Merrett[6], Amitabha Das[6]. **Liverpool Hospital** Peter H. Cosman[7], Kasim Ismail[7]. **St Vincent's Hospital** Alina Stoita[8], David Williams[8], Allan Spigellman[8]**. Westmead Hospital** Duncan McLeod[9], Judy Kirk[9]. **Royal Prince Alfred Hospital, Chris O'Brien Lifehouse** James G. Kench[10], Peter Grimison[10], Charbel Sandroussi[10], Annabel Goodwin[7,10]. **Prince of Wales Hospital** R. Scott Mead[1,11], Katherine Tucker[11], Lesley Andrews[11]. **Fiona Stanley Hospital** Michael Texler[12], Cindy Forrest[12], Mo Ballal[12,13], David Fletcher[12]**. St John of God Healthcare** Maria Beilin[13], Kynan Feeney[13] Krishna Epari[13] Sanjay Mukhedkar[13]. **Epworth HealthCare** Nikolajs Zeps[23]. **Royal Adelaide Hospital** Nan Q Nguyen[14], Andrew R. Ruszkiewicz[14], Chris Worthley[14]. **Flinders Medical Centre** John Chen[15], Mark E. Brooke-Smith[15], Virginia Papangelis[15]. **Envoi Pathology** Andrew D. Clouston[16]. **Princess Alexandra Hospital** Andrew P. Barbour[17], Thomas J. O'Rourke[17], Jonathan W. Fawcett[17], Kellee Slater[17], Michael Hatzifotis[17], Peter Hodgkinson[17]. **Austin Hospital** Mehrdad Nikfarjam[18]. **Johns Hopkins Medical Institutes** James R. Eshleman[19], Ralph H. Hruban[19], Christopher L. Wolfgang[19]. **ARC-Net Centre for Applied Research on Cancer** Aldo Scarpa[20], Rita T. Lawlor[20], Vincenzo Corbo[20], Claudio Bassi[20]. **University of Glasgow** Andrew V Biankin [21], Nigel B. Jamieson[21] David K. Chang[1, 21,] Stephan B. Dreyer[21].

[1]The Kinghorn Cancer Centre, Garvan Institute of Medical Research, 370 Victoria Street, Darlinghurst, Sydney, New South Wales 2010, Australia.
[2]QIMR Berghofer Medical Research Institute, 300 Herston Rd, Herston, Queensland 4006, Australia.
[3]University of Melbourne, Centre for Cancer Research, Victorian Comprehensive Cancer Centre, 305 Grattan Street, Melbourne, Victoria 3000, Australia.
[4] Institute for Molecular Bioscience, University of QLD, St Lucia, Queensland 4072, Australia.
[5]Royal North Shore Hospital, Westbourne Street, St Leonards, New South Wales 2065, Australia.
[6]Bankstown Hospital, Eldridge Road, Bankstown, New South Wales 2200, Australia.
[7]Liverpool Hospital, Elizabeth Street, Liverpool, New South Wales 2170, Australia.
[8] St Vincent's Hospital, 390 Victoria Street, Darlinghurst, New South Wales, 2010 Australia.
[9]Westmead Hospital, Hawkesbury and Darcy Roads, Westmead, New South Wales 2145, Australia.
[10]Royal Prince Alfred Hospital, Missenden Road, Camperdown, New South Wales 2050, Australia.
[11]Prince of Wales Hospital, Barker Street, Randwick, New South Wales 2031, Australia.
[12]Fiona Stanley Hospital, 11 Robin Warren Dr, Murdoch WA 6150

[13] St John of God Healthcare, 12 Salvado Road, Subiaco, Western Australia 6008, Australia.
[14] Royal Adelaide Hospital, North Terrace, Adelaide, South Australia 5000, Australia.
[15] Flinders Medical Centre, Flinders Drive, Bedford Park, South Australia 5042, Australia.
[16] Envoi Pathology, 1/49 Butterfield Street, Herston, Queensland 4006, Australia.
[17] Princess Alexandra Hospital, 199 Ipswich Rd, Woolloongabba QLD 4102
[18] Austin Hospital, 145 Studley Road, Heidelberg, Victoria 3084, Australia.
[19] Johns Hopkins Medical Institute, 600 North Wolfe Street, Baltimore, Maryland 21287, USA.
[20] ARC-NET Center for Applied Research on Cancer, University of Verona, Via dell'Artigliere, 19 37129 Verona, Province of Verona, Italy.
[21] Wolfson Wohl Cancer Research Centre, Institute of Cancer Sciences, University of Glasgow, Garscube Estate, Switchback Road, Bearsden, Glasgow, Scotland G61 1BD, United Kingdom.
[22] Wollongong Hospital, Illawarra and Shoalhaven Local Health District, Loftus Street, Wollongong NSW 2500.
[23] Epworth HealthCare, 89 Bridge Rd, Richmond VIC 3121, Australia

Avner Australian Pancreatic Cancer Matrix Atlas (APMA) consortium members:

Paul Timpson[1], Thomas R. Cox[1], Marina Pajic[1], Anthony J. Gill[1,2], Jaswinder S. Samra[1,2], Brooke A. Pereira[1], David Herrmann[1], Amber L. Johns[1], Gloria Jeong[1], Shona Ritchie[1], Daniel A. Reed[1], Cecilia R. Chambers[1], Janett Stoehr[1], Morghan C. Lucas[1], Joanna N. Skhinas[1], Lea Abdulkhalek[1], Max Nobis[1], Tatjana Schmitz[1], Victoria Lee[1], Xanthe L. Metcalf[1], Sean M Grimmond[3], Kym Pham Stewart[3], Mehreen Arshi[1], Angela M Steinmann[1], Nicola Blackburn[1], Ruth J. Lyons[1]

[1]The Kinghorn Cancer Centre, Garvan Institute of Medical Research, 370 Victoria Street, Darlinghurst, Sydney, New South Wales 2010, Australia.
[2]Royal North Shore Hospital, Westbourne Street, St Leonards, New South Wales 2065, Australia.
[3]University of Melbourne Centre for Cancer Research, Victorian Comprehensive Cancer Centre, 305 Grattan Street, Melbourne, Victoria, 3000, Australia
